# Design, Synthesis, Biological Evaluation and Molecular Docking Studies of a New Series of Maleimide Derivatives

**DOI:** 10.1002/open.202400058

**Published:** 2024-09-23

**Authors:** Öznur Eyilcim, Fulya Günay, Yuk Yin Ng, Özlem Ulucan Açan, Zuhal Turgut, Ömer Tahir Günkara

**Affiliations:** ^1^ Department of Chemistry Faculty of Arts & Science Yıldız Technical University Davutpaşa Campus 34220 Esenler Istanbul Türkiye; ^2^ Food Technology Programme Vocational School of Health Services Üsküdar University Carsi Campus Üsküdar Istanbul Türkiye; ^3^ Department of Genetics and Bioengineering Faculty of Engineering and Natural Sciences Istanbul Bilgi University Istanbul Türkiye; ^4^ Hogeshooldocent Life Science Instıtue for Life Science & Chemistry HU University of Applied Sciences Utrecht Utrecht Netherlands

**Keywords:** Breast cancer, GSK-3β inhibitors, Maleimide derivatives, MCF-7, MDA-MB-231

## Abstract

A series of novel maleimide derivatives were synthesized, with various heterocyclic compounds serving as side chains in the synthesis process. The structural characteristics of these compounds were elucidated through the application of ^1^H‐NMR spectroscopy, ^13^C‐NMR (APT) spectroscopy, and high‐resolution mass spectrometry (HRMS). The anti‐cancer potential of these compounds was subsequently assessed in vitro, utilizing two distinct breast cancer cell lines, namely MDA‐MB‐231 and MCF‐7, via MTT assay. Among the 12 newly synthesized compounds, **4 a**, **4 b**, **4 c**, **4 d**, **5 a**, **5 b**, **5 c** and **5 d** were determined to show the most promising anti‐cancer activity against both breast cancer cell lines. Moreover, the morphological changes induced in the cells following a 24‐hour incubation period with these compounds were observed using light microscopy. Additionally, molecular dynamics simulations were conducted to assess the stability of the bound conformations of the compounds to the target protein GSK‐3β as obtained through molecular docking calculations.

## Graphical abstract

Twelve novel maleimide derivatives (**4 a**–**e** and **5 a**–**e**) were synthesized. Their anticancer activities were evaluated against two different breast cancer cell lines (MCF‐7 and MDA‐MB‐231). Molecular dynamics simulations in complex with GSK‐3β were carried out to evaluate the stability of the interactions between the protein and the promising compounds. **5 a** exhibits the most robust interactions with GSK‐3β among the compounds.

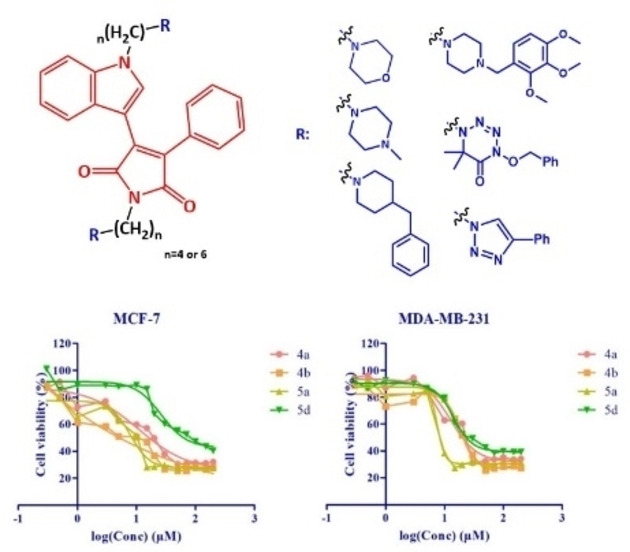

## Introduction

Cancer is a health problem that millions of people suffer from in the world and many cancer cells in the body grow uncontrollably and spread to other parts of the body.^[1]^ According to the World Health Organization (WHO) estimation in 2018, cancer is responsible for 9.6 million deaths (one in six deaths) and is the second leading cause of death worldwide. Lung, prostate, colorectal, stomach and liver cancer are the most common types of cancer in men, while breast, colorectal, lung, cervical and thyroid cancer are the most common among women.^[2]^ In addition, according to the statistics of the WHO, 685,000 of the 2.3 million women diagnosed with breast cancer worldwide died in 2020. Between 2015 and 2020, 7.8 million women who were diagnosed with breast cancer survived, these statistics show breast cancer as the most common cancer in the world.^[3]^ Despite great advances in the early detection and surgical treatment of breast cancer, breast cancer is still defined as an incurable disease with survival times ranging from one to four years depending on the subtype, suggesting a significant unfulfilled medical need.[^4^, ^5^] Consequently, there is an urgent need to identify new targeted therapeutic agents.

Glycogen synthase kinase‐3 (GSK‐3) is a multifunctional serine/threonine kinase. Aberrant expression of GSK3 is associated with many human diseases, including cancer. GSK‐3 plays a role or is involved in the regulation of protein synthesis, glycogen metabolism, cell motility, proliferation, protein translation, apoptosis, cell cycle progression and survival.[^6^, ^7^, ^8^, ^9^] There are two isoforms of GSK‐3, GSK‐3α and GSK‐3β, which are encoded by different genes and are 85 % identical.^[6]^ Despite having a high degree of similarity, the biochemical functions of these isoforms are different. It has also been reported that the isoform, GSK‐3β dysregulation plays a role in the development of a various human diseases such as diabetes mellitus, cardiovascular diseases, some neurodegenerative diseases, and bipolar disorder. This therapeutic potential of GSK‐3β inhibitors has made them a significant research area.^[10]^ In addition, several studies have demonstrated that inhibition of GSK‐3β, which has a therapeutic effect against 25 different cancer cells, protects normal cells and tissues from the harmful effects of cancer treatments.^[11]^ Several studies have reported overexpression of various protein kinases in primary and metastatic breast cancers. In the last decade research GSK‐3β is overexpressed in breast cancer as it plays an important role in cancer cell proliferation and survival.[^12^, ^13^, ^14^, ^15^, ^16^, ^17^] GSK‐3β inhibition also overcomes chemoresistance in human breast cancer.^[18]^ Therefore, GSK‐3β inhibition is a potential approach to target uncontrolled breast cancer cell proliferation.

**Figure 1:**
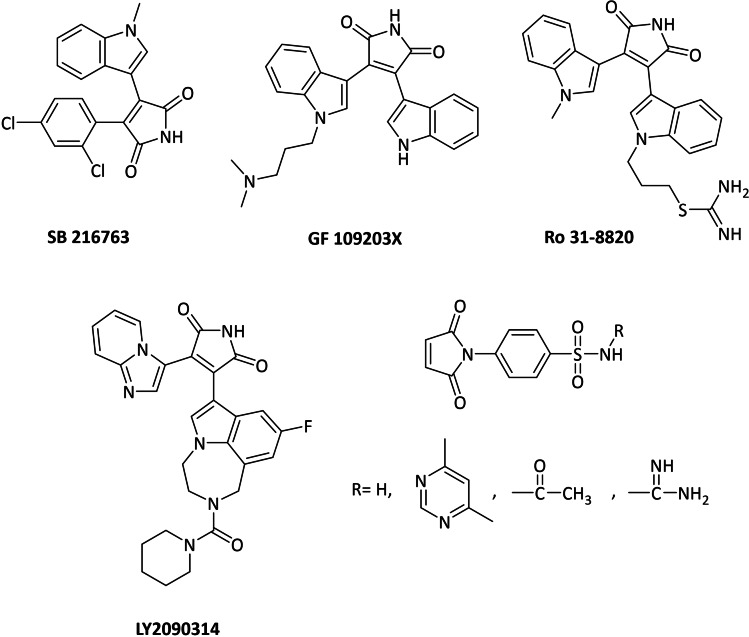
SB 216763, GF 109203X, Ro 31‐8820, LY2090314, maleimide benzenesulfonamide derivatives (GSK‐3β inhibitors).

A variety of chemical inhibitors from both synthetic either natural source has been reported as GSK‐3β inhibitors, such as maleimides,^[19]^ pyrimidines,^[20]^ pyrimidin‐4‐ones,^[21]^ purines^[22]^ and indirubins.^[23]^ Maleimide motifs are widely present in natural products and pharmaceutical compounds, such as GSK‐3β inhibitors, antibacterial agents, antifungal agents.[^24^, ^25^] For example, SB 216763, which is potent, selective, and ATP‐competitive for both GSK‐3α and GSK‐3β, has been reported to be a new target for the prevention of heart and kidney damage by suppressing aldosterone‐induced heart and kidney injury (Figure 1).^[26]^ GF 109203X and Ro 31‐8820 compounds, another maleimide derivatives, have also been developed as potent GSK‐3β inhibitors. These bisindolylmaleimides are based on staurosporine, a cell‐permeable alkaloid exhibiting anti‐cancer activity (Figure 1).[^27^, ^28^, ^29^, ^30^] LY2090314, an imidazo[1,2‐a]pyridinyl‐indolyl‐maleimide derivative, plays an important role in multiple pathways, including protein synthesis initiation, cell proliferation, cell differentiation and apoptosis, and has reached preclinical trials for the treatment of diabetes (Figure 1).^[31]^ It has been reported that 4‐azaindolyl‐indolyl‐maleimide derivatives also show GSK‐3β inhibition activity and the addition of a nitrogen atom to the 4‐position of the Indole ring can significantly increase the selectivity for GSK‐3β.^[32]^ Recent studies show that maleimide benzenesulfonamide derivatives can also be used as preventive agents against breast cancer by giving important interactions with aromatase protein such as hydrophobic interactions and Van der Waals forces (Figure 1).^[33]^ Therefore, it would be beneficial to synthesize indole‐linked maleimide derivatives and to screen for their inhibition potential.

When comparing the inhibitory activity, it was revealed that adding suitable hydrophilic side chains to the ^1^N position of the indole ring provided a significant increase in GSK‐3β inhibitory activity. It has also been observed that different substitutions on the indole ring may affect GSK‐3β inhibitory activity.^[34]^ Additionally, interestingly, *N*‐substituted maleimide derivatives evaluated as GSK‐3β inhibitors have not been encountered. Our aim is the synthesis of new compounds containing both four or six‐chain *N*‐substituted maleimide and *N*‐substituted indole derivatives, and evaluation of their GSK‐3β inhibitor properties. These facts prompted us to design and synthesis a of twenty series of novel compounds. All synthesized compounds were evaluated for GSK‐3β inhibition effect to proliferation/survival on breast cancer cells MDA‐MB‐231 and MCF‐7. Docking studies of the all compounds has also been carried out against GSK‐3β site to understand the possible modes of molecular interactions.

## Results and Discussion

### Chemistry

**Scheme 1:**
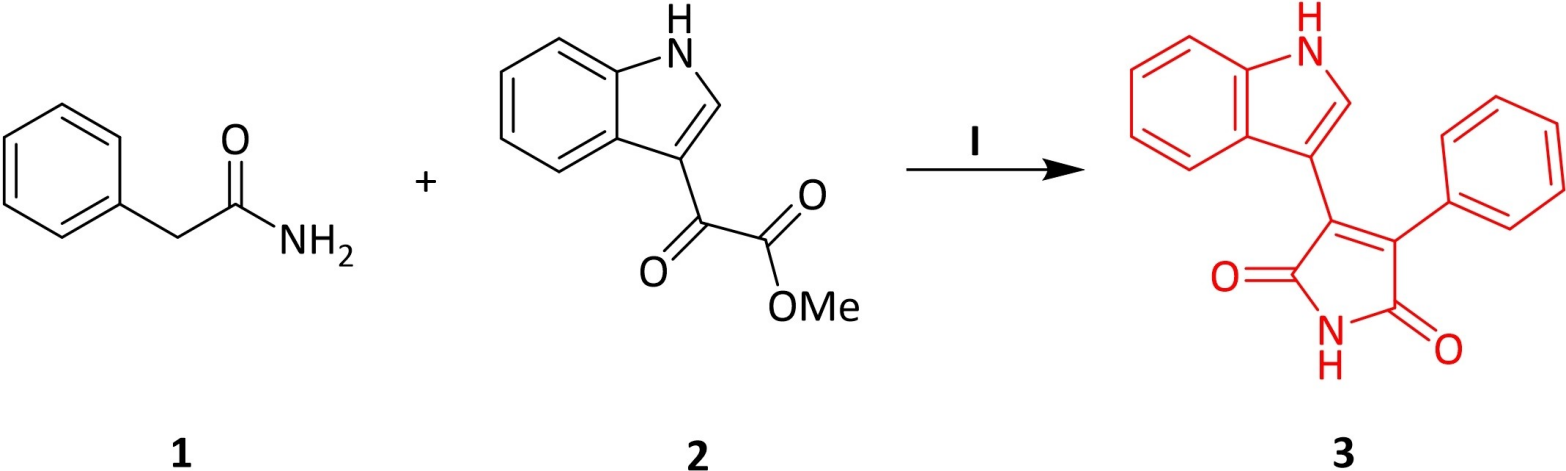
Synthesis of **3** ^a^. ^a^ Reagents and conditions: (I) (i) *t*‐BuOK, dry THF, 0 °C to rt; (ii) NH_4_Cl.^[37]^

**Scheme 2:**
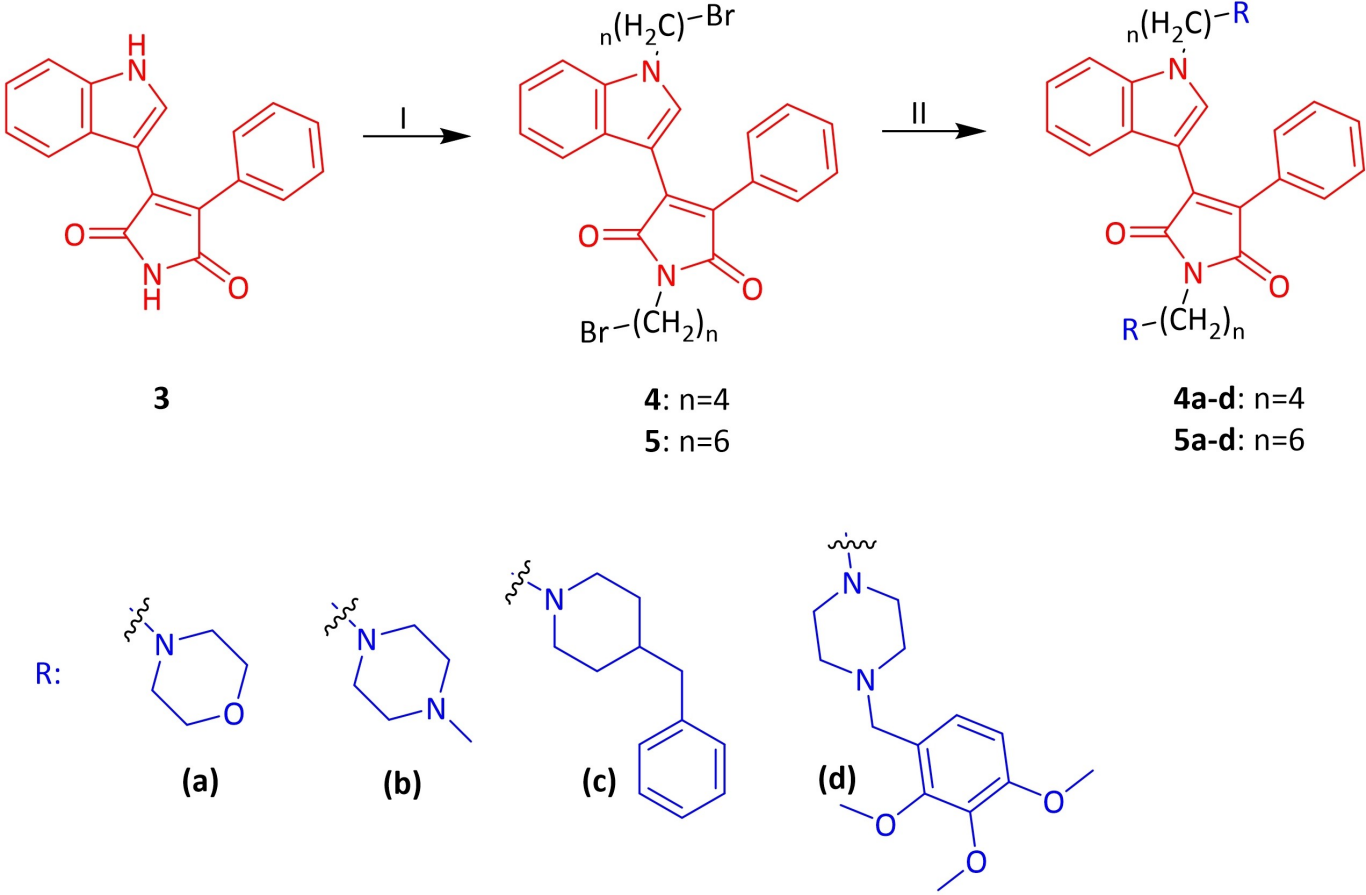
Synthesis of target maleimide compounds **4 a**–**d** ^a^ and **5 a**–**d** ^b^. ^a^ Reagents and conditions: (I) 1,4‐Dibromobutane, dry acetone, K_2_CO_3_, 55 °C, reflux; 51 %. (II) Cycloalkyl amines, dry acetone, K_2_CO_3_, 55 °C, reflux, N_2_; 48–86 %. ^b^ Reagents and conditions: (I) 1,6‐Dibromohexane, dry acetone, K_2_CO_3_, 55 °C, reflux; 88 %. (II) Cycloalkyl amines, dry acetone, K_2_CO_3_, 55 °C, reflux, N_2_; 75–84 %.

**Scheme 3:**
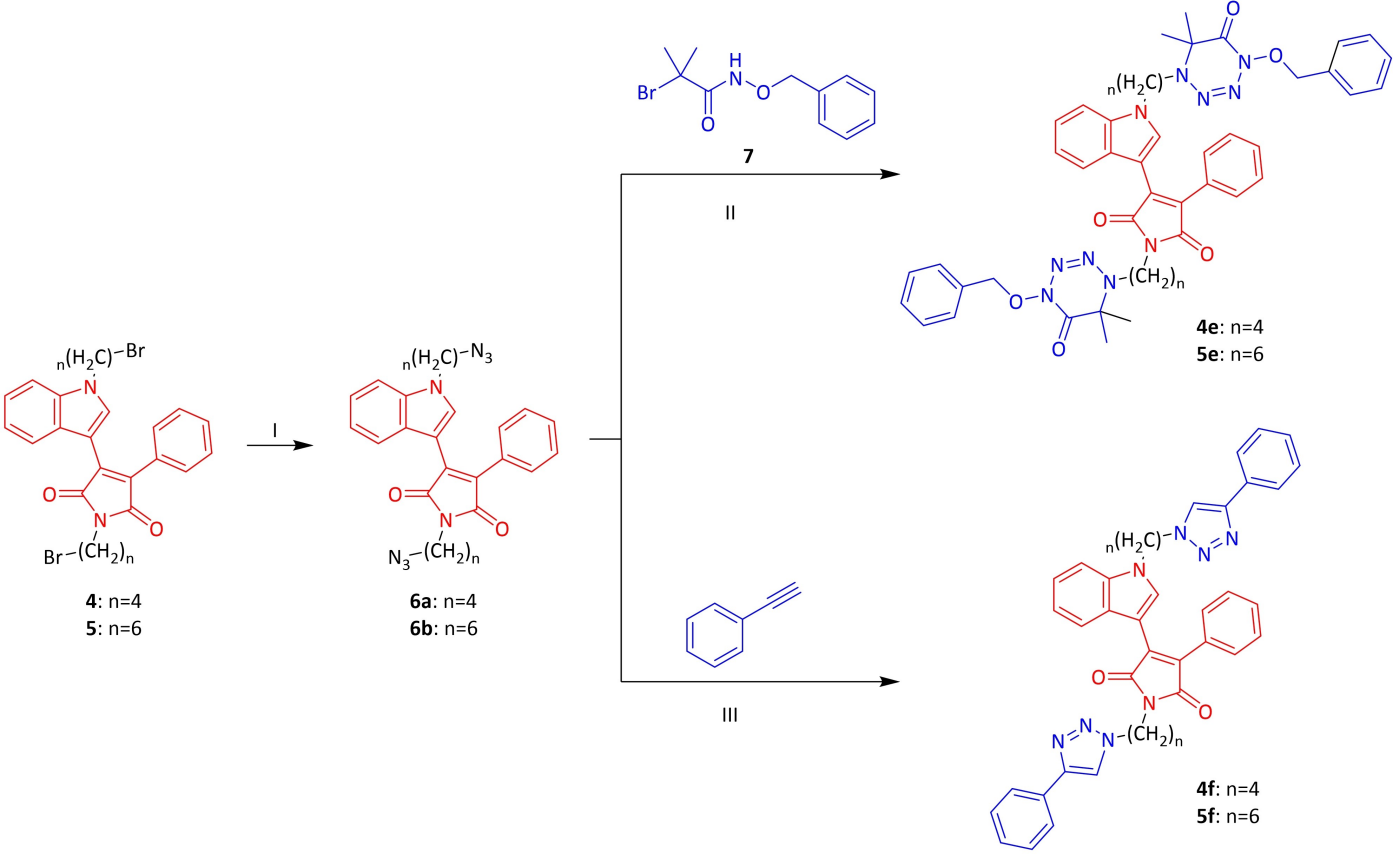
Synthesis of target maleimide compounds **4 e**–**f** and **5 e**–**f**.

2‐Phenylacetamide **1**, one of the starting materials, was synthesized by the known method in the presence of phenylacetic acid, thionyl chloride and ammonia solution under suitable conditions.^[35]^ The other starting compound, methyl 2‐(1*H*‐indol‐3‐yl)‐2‐oxoacetate **2** was obtained by the reaction of indole with oxalyl chloride and methanol in dry diethyl ether.^[36]^ Compounds **1** and **2** were reacted by conventional method at room temperature under a nitrogen atmosphere in dry THF using *t*‐BuOK as the base to form phenyl indolyl maleimide **3** (Scheme 1).^[37]^ In this study, the synthesis of four and six chain maleimide derivatives were aimed. For this purpose, the maleimide and indole −NH groups of **3** were first refluxed to give **4** by reacting with 1,4‐dibromobutane in the presence of dry acetone and K_2_CO_3_ base. Moreover, under the same reaction conditions, the reaction was carried out using 1,6‐dibromohexane to form **5** in 88 % yield. The bromine groups of **4** and **5** were substituted with cycloalkyl amines such as morpholine, *N*‐methyl piperazine, 4‐benzyl piperidine, trimetazidine dihydrochloride to obtain the corresponding *N*‐substituted **4 a**–**d** and **5 a**–**d** maleimide compounds (Scheme 2). Different synthesis methods were used in the synthesis of **4 e**–**f** and **5 e**–**f** compounds, which are another *N*‐substituted indole and maleimide derivatives. In the first step, compounds **6 a**–**b** obtained from the nucleophilic substitution with **4** and **5** were directly used in the next step without purification. Furthermore, compounds **4 f** and **5 f** were formed by giving cycloaddition reaction with azide derivatives and phenyl acetylene in a benzoic acid‐promoted and copper (I)‐catalyzed medium (Scheme 3).

The crude products were purified by column chromatography on silica gel to afford the pure compounds. All the structures of these derivatives were confirmed by spectral data (FT‐IR, ^1^H‐NMR, ^13^C‐NMR (APT) and HRMS).

## Biological Activity

### MTT Assay

Anti‐proliferative inhibitory effects of newly synthesized compounds against breast cancer cells were determined by MTT test. The MTT test showed that both MCF‐7 and MDA‐MB‐231 cells were sensitive to 8 out of 12 newly synthesized compounds in‐vitro. Among them, **4 a**, **4 b**, and **5 d** have IC_50_ values below 30 μM (16.94 μM, 16.80 μM, and 29. 28 μM respectively, for MCF‐7; and 12.95 μM, 18.71 μM and 14.35 μM, respectively, for MDA‐MB‐231). The IC_50_ values of **4 c**, **4 d**, **5 a**, **5 b**, and **5 c** were found below 10 μM (3.21 μM, 6.215 μM, 7.418 μM, 5.331 μM and 4.000 μM, respectively, for MCF‐7; and 1.651 μM, 3.055 μM, 4.367 μM, 6. 571 μM and 7.579 μM, respectively, for MDA‐MD‐231).

**Figure 2:**
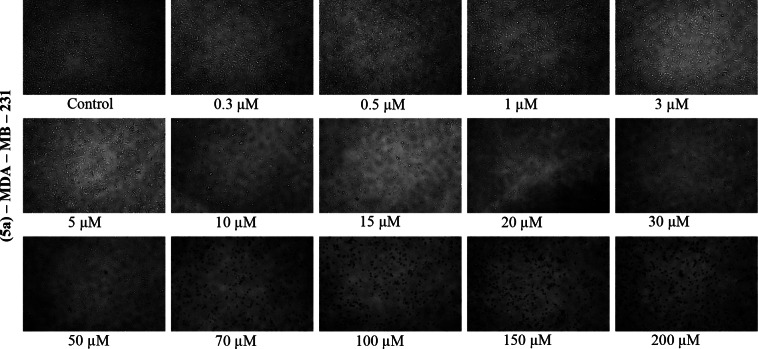
Microscope images of Compound **5 a** in MDA‐MB‐231 cell line prepared at various concentrations.

**Figure 3:**
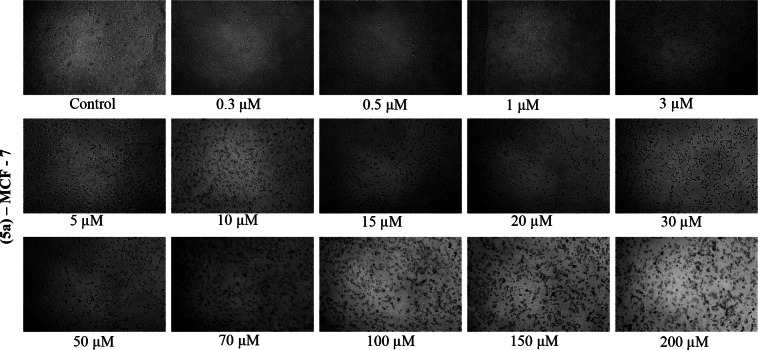
Microscope images of Compound **5 a** in MCF‐7 cell line prepared at various concentrations.

Microscope images for each concentration of each cell in the working range were given in the supporting information (see Figure S68–S91). Also Figure 2 and Figure 3 showed that microscope images of Compound **5 a** in MDA‐MB‐231 and MCF‐7 cell lines prepared at various concentrations. Under the microscope, the living cells (MDA‐MB‐231 and MCF‐7) appear branched around a nucleus, while cells that die after drug application and 24 hours of incubation appear as dots. It has been observed that as the drug concentration increases, the dying cells come together in clusters.

**Figure 4:**
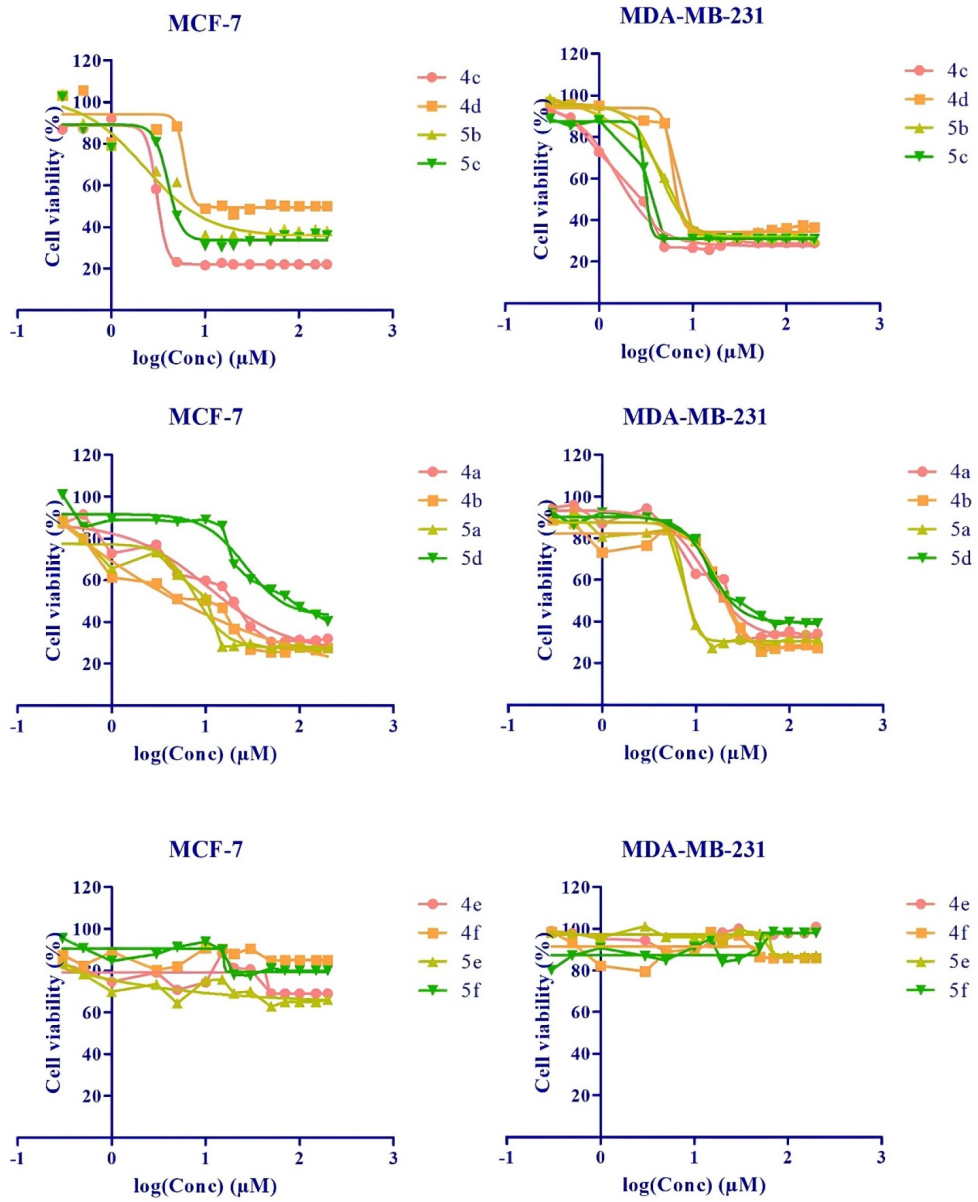
Effect of new maleimid derivatives **4 a**–**f** and **5 a**–**f** on MCF‐7 and MDA‐MB‐231 cell lines.

Substituents with different alkyl chains in the maleimide and indole ring could affect the inhibitory potency. Nitrogen‐containing heterocycles may be used as possible chemotherapy agents for cancer.^[38]^ In this study, the responses of organic compounds carrying different numbers of nitrogen atoms in their side chains that bind to maleimide moiety were compared by the MTT test. Figure 4 showed that effect of new maleimide derivatives **4 a**–**f** and **5 a**–**f** after 24 h of incubation on MCF‐7 and MDA‐MB‐231 cell lines. According to the IC_50_ value (Table 1), the potential of these aromatic groups to inhibit the proliferation of breast cancer cells can be listed as follows, from the most effective to the least effective. 1‐Benzyl piperidine (**4 c**, **5 c**), methyl piperazine (**4 b**, **5 b**), 2‐(3,4,5‐trimethoxyphenyl)methyl piperazine (**4 d**, **5 d**) and morpholine (**4 a**, **5 a**) side chains. It was determined that tetrazine (**4 e**, **5 e**) and phenyl triazole (**4 f**, **5 f**) side chains did not show any effect against the examined breast cancer cells.

**Table 1:** The IC_50_ values of 12 novel maleimide derivatives **4 a**–**f** and **5 a**–**f** on breast cancer cell lines (MDA‐MB‐231 and MCF‐7).

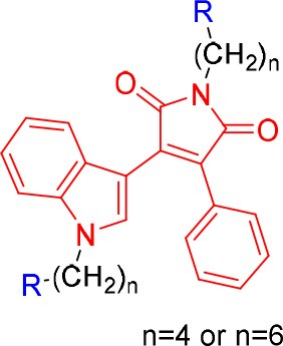						
R	No	MCF‐7 IC_50_ (μM)	MDA‐MB‐231 IC_50_ (μM)	No	MCF‐7 IC_50_ (μM)	MDA‐MB‐231 IC_50_ (μM)
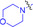	**4 a**	16.94±0.3088	12.95±0.1637	**5 a**	7.418±0.2127	7.579±0.1143
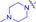	**4 b**	16.80±0.0958	18.71±0.0793	**5 b**	5.331±0.3882	4.367±0.0568
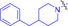	**4 c**	3.209±0.0535	1.651±0.3973	**5 c**	4.000±0.1065	3.055±0.0811
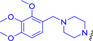	**4 d**	6.215±0.5805	6.571±0.1727	**5 d**	29.28±0.2464	14.35±0.0592
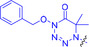	**4 e**	>200	>200	**5 e**	>200	>200
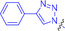	**4 f**	>200	>200	**5 f**	>200	>200

* The data are presented as mean values from experiments conducted in 6 replicates **4 a**–**f** and **5 a**–**f**.

Among the synthesized compounds carrying morpholine (**4 a**, **5 a**) and methyl piperazine (**4 b**, **5 b**), groups, as the length of the intermediate hydrocarbon chain increases, the effect of inhibiting breast cancer cells increases at lower concentrations. For the benzyl piperidine (**4 c**, **5 c**) group, the variation of the intermediate hydrocarbon chain length did not affect the results. For compounds carrying 2‐(3,4,5‐trimethoxyphenyl)methyl piperazine (**4 d**, **5 d**) group, the ability to inhibit breast cancer decreases as the length of the intermediate hydrocarbon chain increases. It is thought that due to the increase in the number of rotatable bonds, the interaction of the molecule with the cell decreases.

To predict possible binding of the compounds to toxicity targets using a collection of protein‐ligand‐based pharmacophores, https://tox‐new.charite.de/protox_II/ website was used. Among these calculations, the parameters (hepatotoxicity, cytotoxicity, carcinogenicity, mutagenicity, immunotoxicity, aromatase, Estrogen Receptor Alpha (ER), Estrogen Receptor Ligand Binding Domain (ER‐LBD), Phosphoprotein (Tumor Supressor) p53) that may be related to breast cancer were shown in Table S4. Accordingly, among the synthesized compounds, **4 a**, **4 c**, **5 a** and **5 c** were calculated as inactive in all parameters. Also, the predicted toxicity classes of the compounds were shown in Table S3.

### Molecular Docking Studies

**Figure 5:**
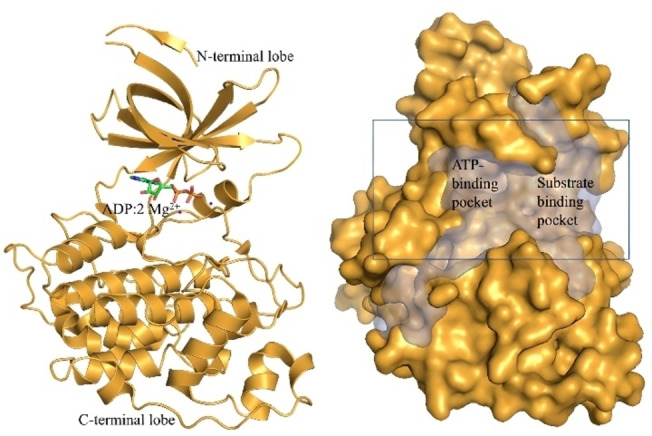
Overall structure of the catalytic domain of GSK‐3β. The pockets were detected using the crystal structure (PDB ID: 4NMO) after removing the ADP and 2 Mg^2+^ ions, using PROPORES.^[40]^

The ATP‐bound form of GSK‐3β, coordinated with the help of 2 Mg^2+^ ions, is the dominant form under physiological conditions.^[39]^ Unfortunately, there is no available crystal structure for this form in the Protein Data Bank. Therefore, we utilized the closest available structure of GSK‐3β, 4NMO (GSK‐3β+ADP+2Mg^2+^) in our calculations where we docked the ligands to the catalytic cleft of the enzyme (Figure 5, region indicated by a rectangle in the right panel). This catalytic cleft, situated between the N‐ and C‐terminal lobes of GSK‐3β, comprises two crucial pockets, namely the ATP binding pocket and the substrate binding pocket, as illustrated in Figure 5. The program PROPORES^[40]^ was utilized to calculate the volume of the large cleft, yielding a value of 3195 Å^3^.

The large size of the cleft, combined with the relatively high number of torsion angles, requires conducting molecular dynamic simulations in addition to molecular docking calculations. We conducted molecular dynamics simulations of **4 c**, **4 d**, **5 a**, **5 b** and **5 c** in complex with GSK‐3β. We chose those compounds based on their IC_50_ values obtained from the MTT assay (IC_50_<10 μM). During the simulations, all five compounds remained bound to the protein. To determine the stability of the docking conformations, we evaluated the RMSF values of the compounds after aligning only the protein conformations.

Table 2 depicts the RMSF values along with their standard deviations. By examining the RMSF value (see Table 2), it becomes evident that compounds **5 a**, **5 b**, and **4 c** exhibit lower RMSF values (<2.0 Å) when compared to compounds **4 d** and **5 c**, which display relatively moderate RMSF values (>2.0 Å).

**Table 2:** Molecular docking results and RMSF values computed from molecular dynamic simulations. ▵G corresponds to the highest‐ranking conformation. RMSF values were computed using heavy atoms of the ligands. Standard deviations are given in the parenthesis.

Compound	ΔG (kcal/mol)	Number of torsions	RMSF (Å)
**4 c**	−8.6	16	1.60 (0.70)
**4 d**	−7.2	16	2.10 (0.90)
**5 a**	−8.7	16	1.10 (0.06)
**5 b**	−7.5	18	1.50 (0.80)
**5 c**	−8.7	20	2.50 (0.90)

A stable conformation for a protein‐ligand complex relies on the interactions between protein residues and a ligand, and this stability can be assessed by employing root mean square fluctuations (RMSF) as a measure. The fluctuations observed in a ligand indicate the extent of flexibility it exhibits. Therefore, lower RMSF fluctuations for a ligand imply lesser flexibility which indicates stable interactions with the protein partner. Taking this into consideration, we can infer that compound **5a** demonstrates the most stable interactions with GSK‐3β among the compounds that have a IC_50_ value smaller than 10 μM.

## Conclusions

In this study, 12 different indole‐substituted maleimide derivatives were designed, synthesized, and MTT tests were performed in two different breast cancer cell lines (MCF‐7 and MDA‐MB‐231) to determine their GSK‐3β inhibitor activities. It was determined that the 8 molecules (**4 a**, **4 b**, **4 c**, **4 d**, **5 a**, **5 b**, **5 c** and **5 d**) for which MTT testing was performed had the best activity and IC_50_ value on both cancer cell lines. Moreover, molecular dynamics simulations of 5 compounds in complex with GSK‐3β were carried out to evaluate the stability of the interactions between the protein and the promising compounds. Compound **5 a** exhibits the most robust interactions with GSK‐3β among the compounds with an IC_50_ value below 10 μM. In summary, our findings make a valuable contribution to forthcoming research in the design and synthesis of GSK‐3β inhibitors.

## Supporting Information

The Supporting Information for this article contains experimental section including chemistry, chemistry datas, FTIR, ^1^H‐NMR ^13^C NMR (APT), HRMS spectra for synthesized compounds, MTT assay studies and datas, cell‐based assay datas, microscope images of compounds, computational methods, Molecular docking and dynamic simulations.

## Conflict of Interests

The authors declare no conflict of interest.

1

## Supporting information

As a service to our authors and readers, this journal provides supporting information supplied by the authors. Such materials are peer reviewed and may be re‐organized for online delivery, but are not copy‐edited or typeset. Technical support issues arising from supporting information (other than missing files) should be addressed to the authors.

Supporting Information

## Data Availability

The data that support the findings of this study are available in the supplementary material of this article.
