# Supplementary material for: Design, Synthesis, Biological Evaluation and Molecular Docking Studies of a New Series of Maleimide Derivatives
Source: ChemistryOpen. 2024 Sep 23;13(12):e202400058. doi: 10.1002/open.202400058 (PMC11625963; doi:10.1002/open.202400058)
Supplement: Supplementary file 1 — Supporting Information [file OPEN-13-e202400058-s001.pdf]

ChemistryOpen

Supporting Information

Design, Synthesis, Biological Evaluation and Molecular Docking Studies of a New Series of Maleimide Derivatives

Öznur Eyilcim, Fulya Günay, Yuk Yin Ng, Özlem Ulucan Açan, Zuhal Turgut, and Ömer Tahir Günkara*

Supporting Information

DESIGN, SYNTHESIS, BIOLOGICAL EVALUATION AND MOLECULAR DOCKING STUDIES OF A NEW SERIES OF MALEIMIDE DERIVATIVES

Öznur Eyilcim [a],[b], Fulya Günay [c], Yuk Yin Ng [d], Özlem Ulucan Açan [c], Zuhal Turgut [a], Ömer Tahir Günkara *[a]

[a] Dr. Ö. Eyilcim, Dr. Z. Turgut, Dr. Ö. T. Günkara
Department of Chemistry, Faculty of Arts & Science
Yıldız Technical University
Davutpaşa Campus, 34220, Esenler, Istanbul, Türkiye
E-mail: gunkara@yildiz.edu.tr

[b] Dr. Ö. Eyilcim
Vocational School of Health Services, Food Technology
Üskudar University
Carsi Campus, Üsküdar, Istanbul, Türkiye

[c] Dr. F. Günay, Dr. Ö. Ulucan Açan
Department of Genetics and Bioengineering, Faculty of Engineering and Natural Sciences
Istanbul Bilgi University
Istanbul, Türkiye

[d] Dr. Y. Y. Ng
Hogeshooldocent Life Science, Instıtue for Life Science & Chemistry,
HU University of Applied Sciences Utrecht,
Utrecht, Netherlands

Content

Experimental Section……………………………………………………………………………………5
Figure S1. FTIR spectrum of compound 1 (ATR)....................12
Figure S2. FTIR spectrum of compound 2 (ATR)....................13
Figure S3. FTIR spectrum of compound 3 (ATR)....................13
Figure S4. FTIR spectrum of compound 4 (ATR)....................14
Figure S5. ^{1}H-NMR spectrum of compound 4 ($CDCl_3$)....................14
Figure S6. APT NMR spectrum of compound 4 ($CDCl_3$)....................15
Figure S7. HR-MS (QTOF) spectrum of compound 4....................15
Figure S8. FTIR spectrum of compound 5 (ATR)....................16
Figure S9. ^{1}H-NMR spectrum of compound 5 ($CDCl_3$)....................16
Figure S10. APT NMR spectrum of compound 5 ($CDCl_3$)....................17
Figure S11. HR-MS (QTOF) spectrum of compound 5....................17
Figure S12. FTIR spectrum of compound 4a (ATR)....................18
Figure S13. ^{1}H-NMR spectrum of compound 4a ($CDCl_3$)....................18
Figure S.14 APT NMR spectrum of compound 4a ($CDCl_3$)....................19
Figure S15. HR-MS (QTOF) spectrum of compound 4a....................19
Figure S16. FTIR spectrum of compound 4b (ATR)....................20
Figure S17. ^{1}H-NMR spectrum of compound 4b ($CDCl_3$)....................20
Figure S18. APT NMR spectrum of compound 4b ($CDCl_3$)....................21
Figure S19. HR-MS (QTOF) spectrum of compound 4b....................21
Figure S20. FTIR spectrum of compound 4c (ATR)....................22
Figure S21. ^{1}H-NMR spectrum of compound 4c ($CDCl_3$)....................22
Figure S22. APT NMR spectrum of compound 4c ($CDCl_3$)....................23
Figure S23. HR-MS (QTOF) spectrum of compound 4c....................23
Figure S24. FTIR spectrum of compound 4d (ATR)....................24
Figure S25. ^{1}H-NMR spectrum of compound 4d ($CDCl_3$)....................24
Figure S26. APT NMR spectrum of compound 4d ($CDCl_3$)....................25
Figure S27. HR-MS (QTOF) spectrum of compound 4d....................25
Figure S28. FTIR spectrum of compound 4e (ATR)....................26
Figure S29. ^{1}H-NMR spectrum of compound 4e ($CDCl_3$)....................26
Figure S30. APT NMR spectrum of compound 4e ($CDCl_3$)....................27
Figure S31. HR-MS (QTOF) spectrum of compound 4e....................27
Figure S32. FTIR spectrum of compound 4f (ATR)....................28
Figure S33. ^{1}H-NMR spectrum of compound 4f ($CDCl_3$)....................28
Figure S34. APT NMR spectrum of compound 4f ($CDCl_3$)....................29
Figure S35. HR-MS (QTOF) spectrum of compound 4f....................29
Figure S36. FTIR spectrum of compound 5a (ATR)....................30
Figure S37. ^{1}H-NMR spectrum of compound 5a ($CDCl_3$)....................30
Figure S38. APT NMR spectrum of compound 5a ($CDCl_3$)....................31
Figure S39. HR-MS (QTOF) spectrum of compound 5a....................31

Figure S40. FTIR spectrum of compound 5b (ATR) 32
Figure S41. ^{1}H-NMR spectrum of compound 5b ($CDCl_3$)....32
Figure S42. APT NMR spectrum of compound 5b ($CDCl_3$) 33
Figure S43. HR-MS (QTOF) spectrum of compound 5b 33
Figure S44. FTIR spectrum of compound 5c (ATR) 34
Figure S45. ^{1}H-NMR spectrum of compound 5c ($CDCl_3$)....34
Figure S46. APT NMR spectrum of compound 5c ($CDCl_3$)....35
Figure S47. HR-MS (QTOF) spectrum of compound 5c 35
Figure S48. FTIR spectrum of compound 5d (ATR) 36
Figure S49. ^{1}H-NMR spectrum of compound 5d ($CDCl_3$)....36
Figure S50. ^{13}C-NMR spectrum of compound 5d ($CDCl_3$) 37
Figure S51. HR-MS (QTOF) spectrum of compound 5d 37
Figure S52. FTIR spectrum of compound 5e (ATR) 38
Figure S53. ^{1}H-NMR spectrum of compound 5e ($CDCl_3$)....38
Figure S54. APT NMR spectrum of compound 5e ($CDCl_3$)....39
Figure S55. HR-MS (QTOF) spectrum of compound 5e 39
Figure S56. FTIR spectrum of compound 5f (ATR) 40
Figure S57. ^{1}H-NMR spectrum of compound 5f ($CDCl_3$) 40
Figure S58. APT NMR spectrum of compound 5f ($CDCl_3$) 41
Figure S59. HR-MS (QTOF) spectrum of compound 5f....41
Figure S60. FTIR spectrum of compound 6a (ATR) 42
Figure S61. HR-MS (QTOF) spectrum of compound 6a 42
Figure S62. FTIR spectrum of compound 6b (ATR) 43
Figure S63. HR-MS (QTOF) spectrum of compound 6b 43
Figure S64. FTIR spectrum of compound 7 (ATR) 44
Figure S65. ^{1}H-NMR spectrum of compound 7 ($CDCl_3$)....44
MTT Assay Studies....45
Figure S66. Bioavailability radar diagram of the synthesized compounds, some of the GSK-3B inhibitor drugs and some of the breast cancer drugs 49
Figure S67. Prediction of permeation of the synthesized compounds by BOILED-Egg (The Brain Or IntestinaL EstimateD) method from http://www.swissadme.ch/ website. (BBB: Blood-Brain Barrier (yellow zone)., HIA: Human Intestinal Absorption (white zone), PGP: Permeability glycoprotein, PGP substrate (PGP+) (blue dot) and PGP non-substrate (PGP-) (red dot)). 50
Figure S68. Microscope images of Compound 4a in MDA-MB-231 cell line prepared at various concentrations....60
Figure S69. Microscope images of Compound 4b in MDA-MB-231 cell line prepared at various concentrations 60
Figure S70. Microscope images of Compound 4c in MDA-MB-231 cell line prepared at various concentration 61
Figure S71. Microscope images of Compound 4d in MDA-MB-231 cell line prepared at various concentrations 61
Figure S72. Microscope images of Compound 4e in MDA-MB-231 cell line prepared at various concentrations....62
Figure S73. Microscope images of Compound 4f in MDA-MB-231 cell line prepared at various concentrations....62
Figure S74. Microscope images of Compound 5a in MDA-MB-231 cell line prepared at various concentrations....63
Figure S75. Microscope images of Compound 5b in MDA-MB-231 cell line prepared at various concentrations 63
Figure S76. Microscope images of Compound 5c in MDA-MB-231 cell line prepared at various concentrations....64
Figure S77. Microscope images of Compound 5d in MDA-MB-231 cell line prepared at various concentrations 64
Figure S78. Microscope images of Compound 5e in MDA-MB-231 cell line prepared at various concentrations....65
Figure S79. Microscope images of Compound 5f in MDA-MB-231 cell line prepared at various concentrations....65
Figure S80. Microscope images of Compound 4a in MCF-7 cell line prepared at various concentrations 66
Figure S81. Microscope images of Compound 4b in MCF-7 cell line prepared at various concentrations 66
Figure S82. Microscope images of Compound 4c in MCF-7 cell line prepared at various concentrations 67
Figure S83. Microscope images of Compound 4d in MCF-7 cell line prepared at various concentrations 67

Figure S84. Microscope images of Compound 4e in MCF-7 cell line prepared at various concentrations 68
Figure S85. Microscope images of Compound 4f in MCF-7 cell line prepared at various concentrations 68
Figure S86. Microscope images of Compound 5a in MCF-7 cell line prepared at various concentrations 69
Figure S87. Microscope images of Compound 5b in MCF-7 cell line prepared at various concentrations 69
Figure S88. Microscope images of Compound 5c in MCF-7 cell line prepared at various concentrations 70
Figure S89. Microscope images of Compound 5d in MCF-7 cell line prepared at various concentrations 70
Figure S90. Microscope images of Compound 5e in MCF-7 cell line prepared at various concentrations 71
Figure S91. Microscope images of Compound 5f in MCF-7 cell line prepared at various concentrations 71
Computational Methods..Fehler! Textmarke nicht definiert.

1. Experimental Section

1.1 General methods of chemistry

All reactions were carried out under inert atmosphere unless otherwise specified. HFIP were purchased from Sigma Aldrich. Other reactant or reagents were purchased from Sigma Aldrich or Merck and used without further purification. The solvents were dried by known techniques. Thin-layer chromatography (TLC) on silica gel GF 254 was used to monitor reaction progress and plates were visualized by exposure to UV light, KMnO4 or Vanillin stain. IR spectra were recorded on a Perkin Elmer FT-IR spectrometer. ^{1}H and ^{13}C NMR spectra were obtained with a Bruker Avance III-500 MHz NMR system. Chemical shifts were reported in parts per million (ppm) with respect to internal standard TMS. Data are reported as follows: chemical shift, multiplicity (s; singlet, d; doublet, t; triplet, q; quartet, m; multiplet, dd; doublet of doublet, dt; doublet of triplet), coupling constants (Hz), number of protons. Mass spectral studies were performed on an Agilent 6890N/5973 GC/IMSD system and high-resolution mass spectra were recorded on an Agilent G6530B TOF/QTOF Mass spectrometer. All crude products were purified with Teledyne Isco CombiFlash Rf 200 system and RediSep Rf Gold Silica columns.

Synthesis of 2-phenylacetamide, (1)[1]

The solution of phenylacetic acid (3.00 g, 22.0 mmol) in dry dichloromethane (CH_2Cl_2) (70.0 mL, 0.314 M) was cooled to 0°C and thionyl chloride (19.2 mL, 264 mmol) was added. After the addition was complete, the reaction mixture was boiled under nitrogen atmosphere for 2 hours. Then the solvent was removed, azeotrope was formed with toluene (2×5 mL) and the solvent was removed again. The mixture was dissolved in tetrahydrofuran (THF) (30.0 mL) and cooled to 0°C. Ammonia solution ($NH_3{\cdot}H_2O$) (40.0 mL, 28-30%) was added dropwise to the reaction medium. The reaction temperature was allowed to come to room temperature and stirred at room temperature for 2 hours. The reaction solvent was evaporated, water (20.0 mL) was added, and stirring was allowed for another 10 minutes while heating. The suspension was cooled to 0°C and filtered. The solid was washed with cold water (20.0 mL). The product was dried in vacuum overnight. White solid (2.66 g, 19.6 mmol); mp.156-158 °C; R_f: 0.51 (1:1 ethyl acetate/n-hexane); yield = 89%.

Synthesis of methyl 2-(1*H*-indol-3-yl)-2-oxaloacetate, (2)[2]

7 g (60 mmol) of indole and 300 mL of dry diethyl ether were added to the reaction flask, respectively, and the temperature of the reaction mixture was brought to 0°C. 16.1 mL of oxalyl chloride was added dropwise to the reaction medium. The reaction was stirred at room temperature for 6 hours until it formed a yellow suspension. The reaction mixture was returned to 0°C, 12 mL of methanol was added dropwise to the mixture and stirred for another 30 minutes. The resulting solid was filtered and washed with cold water to give a yellow solid (10.93 g, 90%).

Synthesis of 3-(1*H*-indol-3-yl)-4-phenyl-1*H*-pyrrole-2,5-dione, (3)[3]

Add compound **1** (226 mg, 1.1 mmol) and **2** (135 mg, 1 mmol) to a double neck flask and dissolve in 4.33 mL dry THF under nitrogen atmosphere to bring the reaction temperature to 0°C. *t*-BuOK (3.03 mL in 1M THF) is then added dropwise to the reaction medium. After the addition is complete, the reaction is allowed to come to room temperature and allowed to stir at room temperature for 15 hours. After 15 hours, 10 mL of saturated NH_4Cl is added to the reaction and stirred for about 30 minutes. The reaction is extracted with ethyl acetate (3x12 mL). The organic phases are combined, washed with saturated brine, dried with Na_2SO_4 and the solvent is removed. The product is dried in vacuum overnight to give an orange solid (285 mg, 19.6 mmol, 99%).

General procedure for the synthesis of compounds 4 and 5

0.6 mmol of **3** was weighed into the reaction flask and dissolved in 8 mL of anhydrous acetone. Anhydrous 6 mmol K_2CO_3 and 2.4 mmol dibromoalkanes (1,4-dibromobutane or 1,6-dibromohexane) were added dropwise. The reaction mixture was allowed to stir under a nitrogen atmosphere at 55 °C overnight under reflux. After a night, the reaction was terminated, and the solvent was removed. The reaction mixture was dissolved in ethyl acetate and extracted with brine, dried over Na_2SO_4, and the organic phase solvent was removed under vacuum. Column chromatography was performed on a 1:3 (ethyl acetate/n-hexane) system.

1-(4-Bromobutyl)-3-(1-(4-bromobutyl)-1*H*-indol-3-yl)-4-phenyl-1*H*-pyrrole-2,5-dione, (4). Orange oily substance; R_f: 0.42 (1:3 ethyl acetate/n-hexane); yield= 51%. FTIR (ATR): v: 3052, 2940, 2867, 1690, 1597, 1514, 1391, 1373, 1360, 1250 cm^{-1}. ^{1}H-NMR ($CDCl_3$, 500 MHz): δ: 1.58-1.36 (m, *J*= 14.8, 7.5 Hz, 2H, CH_2), 1.89–1.83 (m, 2H, CH_2), 1.96–1.90 (m, 2H, CH_2), 2.13–2.05 (m, 2H, CH_2), 3.42 (d, *J*= 12.9 Hz, 2H, CH_2), 3.47 (t, *J*= 6.4 Hz, 2H, CH_2), 3.70 (t, *J*= 6.8 Hz, 2H, CH_2), 4.23 (t, *J*= 7.0 Hz, 2H, CH_2), 6.43 (d, *J*= 8.1 Hz, 1H, ArH), 6.79 (t, *J*= 8.0 Hz, 1H, ArH), 7.16 (t, *J*= 7.7 Hz, 1H, ArH), 7.34–7.29 (m, 4H, ArH), 7.51 (d, *J*= 7.9 Hz, 2H, ArH), 7.96 (s, 1H, ArH) ppm. APT (125 MHz, $CDCl_3$): δ: 26.33 (CH_2), 27.51 (CH_2), 28.83 (CH_2), 28.87 (CH_2), 31.69 (CH_2), 31.98 (CH_2), 36.22 (*N*-CH_2), 45.11 (*N*-CH_2), 103.79 (Cq), 108.69 (CAr), 119.59 (CAr), 121.57 (CAr), 121.75 (CAr), 123.99 (Cq), 127.21 (2xCAr), 127.42 (Cq), 127.96 (CAr), 128.90 (2xCAr), 129.50 (Cq), 130.49 (Cq), 132.27 (CAr), 135.55 (Cq), 170.79 (C=O), 170.82 (C=O) ppm. HR-MS (ESI-QTOF): m/z $[M+H]^+$, calculated: ($[C_{26}H_{26}Br_2N_2O_2]$+H)+: 559.3220, found: 559.0410.

Synthesis of 1-(6-bromohexyl)-3-(1-(6-bromohexyl)-1*H*-indol-3-yl)-4-phenyl-1*H*-pyrrole-2,5-dione, (5). Orange oily substance; R_f: 0.48 (1:3 ethyl acetate/n-hexane); yield = 88%. FTIR (ATR): v: 3052, 2933, 2856, 1692, 1619, 1514, 1491, 1436, 1392, 1370, 1298 cm^{-1}. ^{1}H-NMR ($CDCl_3$, 500 MHz): δ: 1.43 (dt, J=8.5, 6.6 Hz, 4H, 2xCH_2), 1.56–1.49 (m, 4H, 2xCH_2), 1.73 (dt, J=14.9, 7.4 Hz, 2H, CH_2), 1.92–1.85 (m, 4H, 2xCH_2), 1.95 (dt, J=14.8, 7.3 Hz, 2H, CH_2), 3.41 (td, *J*=6.7, 4.7 Hz, 4H, 2xBrCH_2), 3.67 (t, *J*=7.2 Hz, 2H, *N*-CH_2), 4.21 (t, *J*=7.1 Hz, 2H, *N*-CH_2), 6.44 (d, *J*=8.1 Hz, 1H, ArH), 6.79 (t, *J*=7.6 Hz, 1H, ArH), 7.16 (t, *J*=7.6 Hz, 1H, ArH), 7.35–7.31 (m, 4H, ArH), 7.53 (dd, *J*=7.2, 2.4 Hz, 2H, ArH), 7.99 (s, 1H, ArH) ppm. APT (125 MHz, $CDCl_3$): δ: 26.0 (CH_2), 26.1 (CH_2), 27.7 (CH_2), 27.8 (CH_2), 28.5 (CH_2), 29.7 (CH_2), 32.4 (CH_2), 32.6 (CH_2), 33.6 (CH_2), 33.8 (CH_2), 38.1 (*N*-CH_2), 46.8 (*N*-CH_2), 104.6 (Cq), 109.7 (CAr), 120.4 (CAr), 122.4 (CAr), 122.7 (CAr), 125.0 (Cq), 128.2 (2xCAr), 128.8 (CAr), 129.9 (2xCAr), 130.7 (2xCq), 131.6 (Cq), 133.4 (CAr), 136.6 (Cq), 171.9 (C=O), 172.0 (C=O) ppm. HR-MS (ESI-QTOF): m/z $[M+H]^+$, calculated: ($[C_{30}H_{34}Br_2N_2O_2]+H)^+$: 615,4300 found: 615.1037.

Synthesis of 1-(4-morpholinebutyl)-3-(1-(4-morpholinbutyl)-1*H*-indol-3-yl)-4-phenyl-1*H*-pyrrole-2,5-dione, (4a)

0.33 mmol (183 mg) of **4** was weighed into a single neck balloon. After it was dissolved in 15 mL of dry acetone under a nitrogen atmosphere, 1.71 mmol (149 mg) of morpholine and 3.8 mmol (526 mg) of anhydrous K_2CO_3 powdered by heating were added. The reaction was refluxed overnight at 55 °C under a nitrogen atmosphere. At the end of the night, TLC control was performed, and it was observed that the starting materials were finished. The reaction was extracted with an ethyl acetate-brine mixture. After drying with Na_2SO_4, the solvent was removed. Purification was carried out by column chromatography in a 1:1 ethyl acetate/methanol system. Orange oily substance; R_f: 0.33 (1:1 ethyl acetate/methanol); yield = 86%. FTIR (ATR): v: 3052, 2940, 2853, 2807, 1693, 1619, 1514, 1491, 1442, 1360, 1298, 1115 cm^{-1}. ^{1}H-NMR ($CDCl_3$, 500 MHz): δ: 1.41–1.37 (m, 2H, CH_2), 1.74 (dt, *J*=14.9, 7.4 Hz, 2H, CH_2), 1.97 (dd, *J*=15.0, 7.4 Hz, 2H, CH_2), 2.49 – 2.36 (m, 10H, CH_2 ve 4x*N*-CH_2), 3.49 (t, *J*=6.7 Hz, 2H, *O*-CH_2), 3.55–3.53 (m, 2H, *O*-CH_2), 3.71–3.69 (m, 10H, 3x*N*-CH_2 ve 2x*O*-CH_2), 4.23 (t, *J*=7.1 Hz, 2H, *N*-CH_2), 6.42 (d, *J*=8.1 Hz, 1H, ArH), 6.78 (t, *J*=7.6 Hz, 1H, ArH), 7.15 (t, *J*=7.6 Hz, 1H, ArH), 7.36–7.31 (m, 4H, ArH), 7.52–7.50 (m, 2H, ArH), 7.98 (s, 1H, ArH) ppm. APT (125 MHz, $CDCl_3$): δ = 23.7 (CH_2), 23.8 (CH_2), 26.6 (CH_2), 27.7 (CH_2), 31.7 (*N*-CH_2), 38.0 (*N*-CH_2),46.8 (*N*-CH_2), 53.68 (2x*N*-CH_2), 58.1 (*N*-CH_2), 58.4 (*N*-CH_2), 61.7 (*N*-CH_2), 66.8 (2x*O*-CH_2), 71.0 (*O*-CH_2), 71.7 (*O*-CH_2), 104.6 (Cq), 109.8 (CAr), 120.4 (CAr), 122.4 (CAr), 122.7 (CAr), 125.0 (Cq), 128.1 (Cq), 128.2 (2xCAr), 128.8 (CAr), 129.9 (2xCAr), 130.6 (Cq), 131.5 (Cq), 133.4 (CAr), 136.6 (Cq), 171.8 (C=O), 171.9 (C=O) ppm. HR-MS (ESI-QTOF): m/z: calculated: ($[C_{34}H_{42}N_4O_4]+H)^+$: 571.3284, found: 571.3272.

Synthesis of 1-(4-(4-methylpiperazin-1-yl)butyl)-3-(1-(4-(4-methylpiperazin-1-yl)butyl)-1*H*-indol-3-yl)-4-phenyl-1*H*-pyrrole-2,5-dione, (4b)

0.47 mmol (262 mg) of **4** was weighed into a single neck balloon. After it was dissolved in 21 mL of dry acetone under a nitrogen atmosphere, 2.17 mmol (217.53 mg) of *N*-methyl piperazine and 5.4 mmol (746 mg) of anhydrous K_2CO_3 powdered by heating were added. The reaction was refluxed overnight at 55 °C under a nitrogen atmosphere. At the end of the night, TLC control was performed, and it was observed that the starting materials were finished. The reaction was extracted with an ethyl acetate-brine mixture. After drying with Na_2SO_4, the solvent was removed. Purification was carried out by column chromatography in a 3:1 chloroform/methanol system. Orange oily substance; R_f: 0.22 (1:1 chloroform/methanol); yield = 70%. FTIR (ATR): v= 3052, 2935, 2875, 2795, 1693, 1619, 1514, 1491, 1443, 1392, 1370, 1298, 1283 cm^{-1}. ^{1}H-NMR ($CDCl_3$, 500 MHz): δ: 1.60–1.54 (m, 4H, 2xCH_2), 1.71 (dd, J = 14.9, 7.4 Hz, 2H, CH_2), 1.94 (dd, *J*= 15.0, 7.4 Hz, 2H, CH_2), 2.29 (s, 6H, 2xCH_3), 2.61–2.35 (m, 20H, 10x *N*-CH_2), 3.68 (t, *J*= 7.0 Hz, 2H, *N*-CH_2), 4.20 (t, *J*= 7.2 Hz, 2H, *N*-CH_2), 6.41 (d, *J*= 8.1 Hz, 1H, ArH), 6.77 (t, *J*= 7.6 Hz, 1H, ArH), 7.14 (t, J= 7.6 Hz, 1H, ArH), 7.33–7.30 (m, 4H, ArH), 7.52–7.48 (m, 2H, ArH), 7.97 (s, 1H, ArH) ppm. APT (125 MHz, $CDCl_3$): δ = 24.2 (CH_2), 26.7 (CH_2), 27.8 (CH_2), 29.6 (CH_2), 38.12 (*N*-CH_2), 45.9 (2x *N*-CH_3), 46.8 (*N*-CH_2), 53.0 (4x*N*-CH_2), 54.9 (4x*N*-CH_2), 57.7 (*N*-CH_2), 58.0 (*N*-CH_2), 104.6 (Cq), 109.8 (CAr), 120.4 (CAr), 122.3 (CAr), 122.7 (CAr), 125.0 (Cq), 128.1 (Cq), 128.2 (2xCAr), 128.8 (CAr), 129.9 (2xCAr), 130.6 (Cq), 131.5 (Cq), 133.4 (CAr), 136.6 (Cq), 171.8 (C=O), 171.9 (C=O) ppm. HR-MS (ESI-QTOF): m/z: calculated: ([$C_{36}H_{48}N_6O_2$]+H)$^+$: 597.3917, found: 597.3905.

Synthesis of 1-(4-(4-benzylpiperidin-1-yl)butyl)-3-(1-(4-(4-benzylpiperidin-1-yl)butyl)-1*H*-indol-3-yl)-4-phenyl-1*H*-pyrrole-2,5-dione, (4c)

0.28 mmol (157 mg) of **4** was weighed into a single neck balloon. After it was dissolved in 12 mL of dry acetone under a nitrogen atmosphere, 1.32 mmol (231 mg) of 4-benzyl piperidine and 3.3 mmol (455,4 mg) of anhydrous K_2CO_3 powdered by heating were added. The reaction was refluxed overnight at 55 °C under a nitrogen atmosphere. At the end of the night, TLC control was performed, and it was observed that the starting materials were finished. The reaction was extracted with an ethyl acetate-brine mixture. After drying with Na_2SO_4, the solvent was removed. Purification was carried out by column chromatography in a 1:1:1 ethyl acetate/n-hexane/methanol system. Orange oily substance; R_f: 0.18 (1:1:1 ethyl acetate/n-hexane/methanol); yield = 72%. FTIR (ATR): v: 3024, 2915, 2847, 2803, 1693, 1619, 1514, 1492, 1443, 1392, 1369, 1298 cm^{-1}. ^{1}H-NMR ($CDCl_3$, 500 MHz): δ: 1.25–1.16 (m, 4H, 2xCH_2), 1.55–1.46 (m, 10H, 5xCH_2), 1.60 (m, 2H, CH_2), 1.78 – 1.72 (m, 4H, 2xCH_2), 1.84–1.80 (m, 2H, 2xCH), 2.28–2.21 (m, 4H, 2x*N*-CH_2), 2.42 (dd, *J*=7.1, 4.0 Hz, 4H, 2xN-CH_2), 2.77 (d, *J*=11.5 Hz, 2H, *N*-CH_2), 2.83 (d, *J*=11.5 Hz, 2H, *N*-CH_2), 3.57 (t, *J*=7.0 Hz, 2H, *N*-CH_2), 4.08 (t, *J*=7.2 Hz, 2H, *N*-CH_2), 6.32 (d, J=8.1 Hz, 1H, ArH), 6.67 (t, *J*=7.3 Hz, 1H, ArH), 7.04–7.01 (m, 6H, ArH), 7.08 (dd, *J*=7.4, 3.7 Hz, 2H, ArH), 7.16 (td, *J*=7.4, 3.3 Hz, 5H, ArH), 7.21 (dd, *J*=6.0, 4.2 Hz, 2H, ArH), 7.43–7.40 (m, 2H, ArH), 7.87 (s, 1H) ppm. APT (125 MHz, $CDCl_3$): δ: 23.3 (CH_2), 23.4 (CH_2), 25.9 (CH_2), 27.0 (CH_2), 31.0 (2xCH_2), 31.1 (2xCH_2), 36.9 (2xCH), 37.1 (CH_2), 42.2 (CH_2), 45.9 (CH_2), 52.9 (2x*N*-CH_2), 53.0 (2x*N*-CH_2), 57.2 (*N*-CH_2), 57.4 (*N*-CH_2), 60.8 (*N*-CH_2), 103.6 (Cq), 108.9 (CAr), 119.5 (CAr), 121.4 (CAr), 121.8 (CAr), 124.0 (Cq), 124.8 (2xCAr), 124.9 (2xCAr), 127.1 (Cq), 127.2 (4xCAr), 127.8 (CAr),128.1 (4xCAr), 129.0 (2xCAr), 129.7 (Cq), 130.7 (Cq), 132.5 (CAr), 135.3 (Cq), 139.6 (Cq), 139.7 (Cq), 170.9 (C=O), 171.0 (C=O) ppm. HR-MS (ESI-QTOF): m/z: calculated: ([$C_{50}H_{58}N_4O_2$]+H)$^+$: 747.4633, found: 747.4625.

Synthesis of 3-phenyl-1-(4-(4-(2,3,4-trimethoxybenzyl)piperazin-1-yl)butyl)-4-(1-(4-(4-(2,3,4-trimethoxybenzyl)) piperazin-1-yl)butyl)-1*H*-indol-3-yl)-1*H*-pyrrole-2,5-dione, (4d)

0.32 mmol (181 mg) of **4** was weighed into a single neck balloon. After dissolving in 15 mL of dry acetone under a nitrogen atmosphere, 1.5 mmol (510 mg) of trimetazidine dihydrochloride and 3.8 mmol (525 mg) of anhydrous K_2CO_3 powdered by heating were added. The reaction was refluxed overnight at 55 °C under a nitrogen atmosphere. At the end of the night, TLC control was performed, and it was observed that the starting materials were finished. The reaction was extracted with an ethyl acetate-brine mixture. After drying with Na_2SO_4, the solvent was removed. Purification was carried out by column chromatography in a 1:1:1 ethyl acetate/n-hexane/methanol system. Orange oily substance; R_f: 0.24 (1:1:1 ethyl acetate/n-hexane/methanol); yield = 48%. FTIR (ATR): v: 3052, 2935, 2873, 2807, 2768, 1694, 1619, 1514, 1493, 1444, 1392, 1363, 1297, 1045 cm^{-1}. ^{1}H-NMR ($CDCl_3$, 500 MHz): δ: 1.51–1.45 (m, 4H, 2xCH_2), 1.62 (dt, *J*=14.8, 7.3 Hz, 2H, CH_2), 1.89–1.82 (m, 2H, CH_2), 2.29 (dd, *J*=13.8, 6.6 Hz, 8H, 4x*N*-CH_2), 2.41 (s, 12H, 6x*N*-CH_2), 3.40 (s, 4H, 2x*N*-CH_2), 3.58 (t, *J*=7.1 Hz, 2H, *N*-CH_2), 3.81–3.74 (m, 18H, 6x*O*-CH_3), 4.11 (t, *J*=7.2 Hz, 2H, *N*-CH_2), 6.32 (d, *J*=8.1 Hz, 1H, ArH), 6.54 (dd, *J*=8.6, 1.7 Hz, 2H, ArH), 6.68 (t, *J*=7.6 Hz, 1H, ArH), 6.89 (d, *J*=8.5 Hz, 2H, ArH), 7.04 (t, J=7.4 Hz, 1H, ArH), 7.23 (dd, *J*=7.2, 5.6 Hz, 4H, ArH), 7.42 (dd, *J*=6.4, 3.2 Hz, 2H, ArH), 7.87 (s, 1H, ArH) ppm. APT (125 MHz, $CDCl_3$): δ : 23.1 (CH_2), 23.2 (CH_2), 25.7 (CH_2), 26.8 (CH_2), 37.1 (*N*-CH_2), 45.8 (*N*-CH_2), 51.7 (4x*N*-CH_2), 52.2 (4x*N*-CH_2), 54.9 (2x*O*-CH_3), 55.3 (*N*-CH_2), 55.4 (*N*-CH_2), 56.7 (*N*-CH_2),

57.0 (N-CH_2), 59.7 (2xO-CH_3), 60.1 (2xO-CH_3), 103.5 (Cq), 105.8 (2xCAr), 118.8 (CAr), 119.3 (CAr), 121.3 (CAr), 121.6 (CAr), 122.7 (Cq), 112.3 (Cq), 123.9 (Cq), 124.1 (2xCAr), 127.0 (Cq), 127.1 (2xCAr), 127.7 (CAr), 128.9 (2xCAr), 129.6 (2xCq), 130.5 (Cq), 132.4 (CAr), 135.6 (2xCq), 141.2 (Cq), 151.6 (Cq), 151.8 (Cq), 151.8 (Cq), 170.8 (C=O), 170.9 (C=O) ppm. HR-MS (ESI-QTOF): m/z: calculated: $([C_{54}H_{68}N_6O_8]+H)^+$: 929.5171, found: 929.5140.

Synthesis of 1-(4-azidobutyl)-3-(1-(4-azidobutyl)-1*H*-indol-3-yl)-4-phenyl-1*H*-pyrrole-2,5-dione, (6a)

0.28 mmol (158 mg) of **4** was dissolved in 9 mL of dry DMF and taken into the reaction flask. 1.38 mmol (90 mg) of NaN_3 was dissolved in 0.9 mL of distilled water and added to the reaction flask. The reaction was stirred at 80°C overnight. According to TLC (1:1 ethyl acetate/n-hexane) result, it was observed that the starting material was finished. After the reaction came to room temperature, it was poured into ice water and extracted with ether-brine. The organic phase was dried with Na_2SO_4, filtered and the solvent was evaporated in vacuo. It was used in the next steps without purification. Orange oily substance; R_f: 0.47 (2:1 ethyl acetate/*n*-hexane); yield = 99%. FTIR (ATR): v: 3052, 2936, 2867, 2092, 1693, 1620, 1515, 1491, 1393, 1361, 1240, 1108 cm^{-1}. HR-MS (ESI-QTOF): m/z: calculated: $([C_{26}H_{26}N_8O_2]+H)^+$: 482.2178, found: 483.2250.

Synthesis of 1-(4-(4-(benzyloxy)-6,6-dimethyl-5-oxo-5,6-dihydro-1,2,3,4-tetrazin-1(4*H*)-yl)butyl)-3-(1-(4-(4-(benzyloxy)-6,6-dimethyl-5-oxo-5,6-dihydro-1,2,3,4-tetrazin-1(4H)-yl)butyl)-1*H*-indol-3-yl)-4-phenyl-1*H*-pyrrole-2,5-dione, (4e)

The resulting 2 mmol (544 mg) *N*-(benzyloxy)-2-bromo-2-methylpropanamide (**7**) and 1 mmol (482 mg) **6a** were taken into a Schlenk tube and 6 mmol (636 mg) anhydrous Na_2CO_3 was added. The air in the tube was removed by applying vacuum to the Schlenk tube, then the tube was filled with nitrogen gas. It was dissolved by adding 3 mL of 1,1,1,3,3,3-hexafluoro-2-propanol solvent with the help of an injector. The reaction was allowed to stir under a nitrogen atmosphere for 12 hours. The reaction was terminated by TLC control. The reaction mixture was filtered through celite, and the solvent was removed in vacuo. It was purified by column chromatography in a 1:1 ethyl acetate/*n*-hexane system. Orange oily substance; R_f: 0.56 (1:1 ethyl acetate/n-hexane); yield = 35%. FTIR (ATR): v= 3032, 2939, 2872, 1694, 1620, 1515, 1491, 1392, 1366, 1241, 1081 cm^{-1}. ^{1}H-NMR ($CDCl_3$, 500 MHz): δ : 1.27 (s, 6H, 2xCH_3), 1.32 (s, 6H, 2xCH_3), 1.78 (m, 6H, 3xCH_2), 1.96 (t, J=7.7 Hz, 2H, CH_2), 3.35 (t, J=6.9 Hz, 2H, N-CH_2), 3.40 (t, J=6.8 Hz, 2H, N-CH_2), 3.69 (t, J=6.5 Hz, 2H, N-CH_2), 4.21 (t, J=7.0 Hz, 2H, N-CH_2), 5.11 (s, 2H, O-CH_2), 5.12 (s, 2H, O-CH_2), 6.44 (d, J=8.1 Hz, 1H, ArH), 6.79 (t, J=8.0 Hz, 1H, ArH), 7.15 (t, J=7.6 Hz, 1H, ArH), 7.36 – 7.31 (m, 10H, ArH), 7.48 – 7.44 (m, 4H, ArH), 7.52 (dd, J=7.4, 2.3 Hz, 2H, ArH), 7.97 (s, 1H, ArH) ppm. APT (125 MHz, $CDCl_3$): δ : 20.4 (2xCH_3), 20.5 (2xCH_3), 25.7 (CH_2), 27.0 (CH_2), 31.7 (CH_2), 37.6 (CH2), 46.5 (N-CH2), 48.1 (N-CH2), 59.8 (N-CH2), 61.8 (N-CH_2), 71.0 (O-CH_2), 71.7 (O-CH_2), 78.4 (2xCq), 104.8 (Cq), 109.7 (CAr), 120.6 (CAr), 122.6 (CAr), 122.8 (CAr), 125.0 (Cq), 125.8 (Cq), 128.2 (2xCAr), 128.4 (3x CAr), 129.0 (2xCAr), 129.1 (2xCAr), 129.4 (CAr), 129.7 (2xCAr), 129.8 (2xCAr), 129.9 (2xCAr), 130.5 (Cq), 131.5 (Cq), 133.8 (Cq), 133.9 (Cq), 136.6 (Cq), 162.1 (C=O), 162.2 (C=O), 171.8 (C=O), 171.9 (C=O) ppm. HR-MS (ESI-QTOF): m/z: calculated: $([C_{48}H_{52}N_{10}O_6]+Na)^+$: 887.3964, found: 887.3962.

Synthesis of 3-phenyl-1-(4-(4-phenyl-1*H*-1,2,3-triazol-1-yl)butyl)-4-(1-(4-(4-phenyl-1*H*-1,2), 3-triazol-1-yl)butyl)-1*H*-indol-3-yl)-1*H*-pyrrole-2,5-dione, (4f)

0.28 mmol (135 mg) of **6a** was weighed into a single neck balloon. 0.6 mmol (61.28 mg) phenyl acetylene, 0.006 mmol (1.5 mg) $CuSO_4.5H_2O$, 0.012 mmol (2.37 mg) sodium ascorbate, 0.06 mmol (7.28 mg) benzoic acid and 1:2 (0.2:0.4 mL) *t*-BuOH:H_2O added. The reaction was stirred at room temperature overnight. At the end of the night, TLC control was performed, and it was observed that the starting materials were finished. The reaction was extracted with dichloromethane. After drying with Na_2SO_4, the solvent was removed. Purification was carried out by column chromatography in a 2:1 ethyl acetate/n-hexane system. Orange oily substance; R_f: 0.3 (2:1 ethyl acetate/*n*-hexane); yield= 65%. FTIR (ATR): v: 3133, 3054, 2920, 2850, 1691, 1620, 1514, 1484, 1464, 1436, 1393, 1361, 1299 cm^{-1}. ^{1}H-NMR ($CDCl_3$, 500 MHz): δ : 1.80–1.74 (m, 2H, CH_2), 2.07–1.94 (m, 6H, 3xCH_2), 3.73 (t, J=6.7 Hz, 2H, N-CH_2), 4.24 (t, J=6.3 Hz, 2H, N-CH_2), 4.35 (t, J=6.5 Hz, 2H, N-CH_2), 4.47 (t, J=7.1 Hz, 2H, N-CH_2), 6.43 (d, J=8.1 Hz, 1H, ArH), 6.80 (t, J=7.6 Hz, 1H, ArH), 7.15 (t, J=7.6 Hz, 1H, ArH), 7.32 (ddd, J=12.8, 7.7, 5.0 Hz, 6H, ArH), 7.41 (td, J=7.6, 2.4 Hz, 4H, ArH), 7.51 (dd, J=7.7, 1.6 Hz, 2H, ArH), 7.64 (s, 1H, ArH), 7.81 (dt, J=7.2, 1.6 Hz, 5H, ArH), 7.93 (s, 1H, ArH) ppm. APT (125 MHz, $CDCl_3$): δ : 25.6 (CH_2), 26.6 (CH_2), 27.6 (CH_2), 27.7 (CH_2), 37.1 (N-CH_2), 46.2 (N-CH_2), 49.5 (N-CH_2), 49.6 (N-CH_2), 104.8 (Cq), 109.8 (CAr), 119.6 (CAr), 119.7 (CAr), 120.7 (CAr), 122.7 (CAr),

122.8 (CAr), 124.9 (Cq), 125.7 (2xCAr), 125.8 (2xCAr), 128.1 (CAr), 128.2 (CAr), 128.3 (2xCAr), 128.6 (Cq), 128.8 (2xCAr), 128.9 (2xCAr), 129.1 (CAr), 129.9 (2xCAr), 130.4 (Cq), 130.5 (Cq), 130.6 (Cq), 131.5 (Cq), 133.3 (CAr), 136.4 (Cq), 147.7 (Cq), 147.9 (Cq), 171.7 (C=O), 171.8 (C=O) ppm. HR-MS (ESI-QTOF): m/z: calculated: $([C_{42}H_{38}N_8O_2]+H)^+$: 687.3190, found: 709.3009.

Synthesis of 1-(6-morpholinohexyl)-3-(1-(6-morpholinohexyl)-1*H*-indol-3-yl)-4-phenyl-1*H*-pyrrole-2,5-dione, (5a)

0.4 mmol (243 mg) of **5** was weighed into a single neck balloon. After it was dissolved in 18 mL of dry acetone under a nitrogen atmosphere, 1.78 mmol (155 mg) of morpholine and 4 mmol (553 mg) of anhydrous K_2CO_3 powdered by heating were added. The reaction was refluxed overnight at 55 °C under a nitrogen atmosphere. At the end of the night, TLC control was performed, and it was observed that the starting materials were finished. The reaction was extracted with an ethyl acetate-brine mixture. After drying with Na_2SO_4, the solvent was removed. Purification was carried out by column chromatography in a 1:1 ethyl acetate/methanol system. Orange oily substance; R_f: 0.24 (1:1:1 ethyl acetate/*n*-hexane/methanol); yield = 82%. FTIR (ATR): v: 3134, 3052, 2931, 2854, 2807, 1693, 1619, 1514, 1491, 1464, 1438, 1392, 1371, 1297, 1045 cm^{-1}. ^{1}H-NMR ($CDCl_3$, 500 MHz): δ 1.42–1.35 (m, 8H, 4xCH_2), 1.53–1.46 (m, 4H, 2xCH_2), 1.73–1.66 (m, 2H, CH_2), 1.96–1.89 (m, 2H, CH_2), 2.32 (dd, J=15.2, 6.4 Hz, 4H, 2x*N*-CH_2), 2.42 (s, 8H, 4x*N*-CH_2), 3.65 (t, *J*=7.2 Hz, 2H, *N*-CH_2), 3.73–3.69 (m, 8H, 4x*O*-CH_2), 4.18 (t, *J*=7.2 Hz, 2H, *N*-CH_2), 6.42 (d, *J*=8.1 Hz, 1H, ArH), 6.77 (t, *J*=7.6 Hz, 1H, ArH), 7.14 (d, *J*=15.3 Hz, 1H, ArH), 7.32 (dt, *J*=5.3, 2.6 Hz, 4H, ArH), 7.52 (dd, *J*=7.3, 2.4 Hz, 2H, ArH), 7.97 (s, 1H, ArH) ppm. APT (125 MHz, $CDCl_3$): δ: 26.3 (CH_2), 26.4 (CH_2), 26.8 (CH_2), 26.9 (CH_2), 27.0 (CH_2), 27.1 (CH_2), 28.6 (CH_2), 29.8 (CH_2), 38.2 (N-CH_2), 46.9 (*N*-CH_2), 53.7 (4x *N*-CH_2), 58.9 (*N*-CH_2), 59.0 (*N*-CH_2), 66.9 (4x*O*-CH_2), 104. 5 (Cq), 109.8 (CAr), 120.4 (CAr), 122.3 (CAr), 122.7 (CAr), 125.0 (Cq), 128.0 (Cq), 128.1 (2xCAr), 128.8 (CAr), 129.9 (2xCAr), 130.7 (Cq), 131.5 (Cq), 133.5 (CAr), 136.6 (Cq), 171.9 (C=O), 172.0 (C=O) ppm. HR-MS (ESI-QTOF): m/z: calculated: $([C_{38}H_{50}N_4O_4]+H)^+$: 627.3905, found: 627.3914.

Synthesis of 1-(6-(4-methylpiperazin-1-yl)hexyl)-3-(1-(6-(4-methylpiperazin-1-yl)hexyl)-1*H*-indol-3-yl)-4-phenyl-1*H*-pyrrole-2,5-dione, (5b)

0.17 mmol (102 mg) of **5** was weighed into a single neck balloon. After it was dissolved in 10 mL of dry acetone under a nitrogen atmosphere, 0.68 mmol (68.11 mg) *N*-methyl piperazine and 2 mmol (276 mg) of anhydrous K_2CO_3 powdered by heating were added. The reaction was refluxed overnight at 55 °C under a nitrogen atmosphere. At the end of the night, TLC control was performed, and it was observed that the starting materials were finished. The reaction was extracted with an ethyl acetate-brine mixture. After drying with Na_2SO_4, the solvent was removed. Purification was carried out by column chromatography in a 3:1 chloroform/methanol system. Orange oily substance; Rf: 0.16 (5:1 chloroform/methanol); yield = 75%. FTIR (ATR): v: 3052, 2929, 2850, 2792, 1694, 1619, 1514, 1491, 1438, 1392, 1371, 1298, 1051 cm^{-1}. ^{1}H-NMR ($CDCl_3$, 500 MHz): δ : 1.44–1.33 (m, 10H, 5xCH_2), 1.50 (dt, *J*=13.6, 6.8 Hz, 4H, 2xCH_2), 1.69 (dt, *J*=14.5, 7.3 Hz, 2H, CH_2), 1.92 (dt, *J*=14.6, 7.3 Hz, 2H, *N*-CH_2), 2.29 (d, *J*=1.6 Hz, 6H, 2x *N*-CH_3), 2.65–2.30 (m, 18H, 9x*N*-CH_2), 3.65 (t, *J*=7.2 Hz, 2H, *N*-CH_2), 4.18 (t, *J*=7.2 Hz, 2H, *N*-CH_2), 6.42 (d, *J*=8.1 Hz, 1H, ArH), 6.77 (t, *J*=7.6 Hz, 1H, ArH), 7.14 (t, *J*=7.6 Hz, 1H, ArH), 7.31 (dd, *J*=8.1, 3.2 Hz, 4H, ArH), 7.53–7.50 (m, 2H, ArH), 7.97 (s, 1H, ArH) ppm. APT (125 MHz, $CDCl_3$): δ : 26.7 (2xCH_2), 26.8 (2xCH_2), 27.1 (CH_2), 27.2 (CH_2), 28.6 (2xCH_2), 29.7 (*N*-CH_2), 29.8 (*N*-CH_2), 38.2 (*N*-CH_2), 45.9 (2xCH_3), 46.9 (*N*-CH_2), 53.1 (2x*N*-CH_2), 55.0 (4x*N*-CH_2), 58.4 (*N*-CH_2), 58.5 (*N*-CH_2), 104.5 (Cq), 109.7 (CAr), 120.4 (CAr), 122.3 (CAr), 122.7 (CAr), 125.0 (Cq), 128.0 (Cq), 128.1 (2xCAr), 128.8 (CAr), 129.9 (2xCAr), 130.7 (Cq), 131.5 (Cq), 133.5 (Cq), 136.6 (Cq), 171.9 (C=O), 172.0 (C=O) ppm. HR-MS (ESI-QTOF): m/z: calculated: $[C_{40}H_{56}N_6O_2]+H)^+$: 653.4538, found: 653.4533.

Synthesis of 1-(6-(4-benzylpiperidin-1-yl)hexyl)-3-(1-(6-(4-benzylpiperidin-1-yl)hexyl)-1*H*-indol-3-yl)-4-phenyl-1*H*-pyrrole-2,5-dione, (5c)

0.24 mmol (140 mg) of **5** was weighed into a single neck balloon. After it was dissolved in 10 mL of dry acetone under a nitrogen atmosphere, 1.14 mmol (200 mg) 4-benzyl piperidine and 3.6 mmol (496 mg) of anhydrous K_2CO_3 powdered by heating were added. The reaction was refluxed overnight at 55 °C under a nitrogen atmosphere. At the end of the night, TLC control was performed, and it was observed that the starting materials were finished. The reaction was extracted with an ethyl acetate-brine mixture. After drying with Na_2SO_4, the solvent was removed. Purification was carried out by column chromatography in a 1:1:1 ethyl acetate/*n*-hexane/methanol system. Orange oily substance; R_f: 0.16 (1:1:1 ethyl acetate/n-hexane/methanol); yield = 84%. FTIR (ATR): v: 3059, 3024, 2922, 2852, 2802, 2767,

1695, 1618, 1514, 1492, 1452, 1392, 1371, 1298 cm^{-1}. ^{1}H-NMR ($CDCl_3$, 500 MHz): δ: 1.42–1.33 (m, 12H, 6xCH_2), 1.57–1.48 (m, 6H, 3xCH_2), 1.71–1.62 (m, 6H, 3xCH_2), 1.96–1.84 (m, 6H, 2xCH ve 2x*N*-CH_2), 2.33 (dd, *J*=15.8, 10.1 Hz, 4H, 2x *N*-CH_2), 2.54 (d, *J*=7.0 Hz, 4H, 2xCH_2), 3.00–2.90 (m, 4H, 2x*N*-CH_2), 3.64 (d, *J*=7.2 Hz, 2H, *N*-CH_2), 4.18 (t, *J*=7.2 Hz, 2H, *N*-CH_2), 6.43 (d, *J*=8.1 Hz, 1H, ArH), 6.78 (t, *J*=7.6 Hz, 1H, ArH), 7.21–7.12 (m, 7H, ArH), 7.34–7.25 (m, 8H, ArH), 7.53 (d, *J*=9.6 Hz, 2H, ArH), 7.98 (s, 1H, ArH) ppm. APT (125 MHz, $CDCl_3$): δ : 25.5 (CH_2), 25.6 (CH_2), 25.8 (CH_2), 26.1 (CH_2), 26.2 (CH_2), 27.6 (CH_2), 28.6 (CH_2), 28.8 (CH_2), 30.7 (CH_2), 30.8 (CH_2), 36.8 (2xCH), 37.2 (CH_2), 42.0 (CH_2), 45.8 (*N*-CH_2), 52.6 (*N*-CH_2), 52.7 (*N*-CH_2), 57.6 (*N*-CH_2), 57.7 (*N*-CH_2), 60.7 (*N*-CH_2), 69.1 (*N*-CH_2), 69.4 (*N*-CH_2), 70.2 (*N*-CH_2), 71.5 (*N*-CH_2), 103.5 (Cq), 108.7 (CAr), 119.3 (CAr), 121.3 (CAr), 121.6 (CAr), 123.9 (Cq), 124.7 (2xCAr), 127.0 (Cq), 127.1 (5xCAr), 127.7 (CAr), 128.0 (5xCAr), 128.9 (2xCAr), 129.7 (2xCq), 130.5 (Cq), 132.4 (CAr), 135.6 (2xCq), 170.8 (C=O), 170.9 (C=O) ppm. HR-MS (ESI-QTOF): m/z: calculated: ($[C_{54}H_{66}N_4O_2]+2H)^{+2}$: 402.2666, found: 402.2658.

Synthesis of 3-phenyl-1-(6-(4-(2,3,4-trimethoxybenzyl)piperazin-1-yl)hexyl)-4-(1-(6-(4-(2,3,4-trimethoxybenzyl)) piperazin-1-yl)hexyl)-1*H*-indol-3-yl)-1*H*-pyrrole-2,5-dione, (5d)

0.21 mmol (132 mg) of **5** was weighed into a single neck balloon. After it was dissolved in 10 mL of dry acetone under a nitrogen atmosphere, 1 mmol (339.3 mg) of trimetazidine dihydrochloride and 2.1 mmol (290 mg) of anhydrous K_2CO_3 powdered by heating were added. The reaction was refluxed overnight at 55 °C under a nitrogen atmosphere. At the end of the night, TLC control was performed, and it was observed that the starting materials were finished. The reaction was extracted with an ethyl acetate-brine mixture. After drying with Na_2SO_4, the solvent was removed. Purification was carried out by column chromatography in a 1:1:1 ethyl acetate/*n*-hexane/methanol system. Orange oily substance; R_f: 0.42 (1:1:1 ethyl acetate/*n*-hexane/methanol); yield = 76%. FTIR (ATR): ν: 3007, 2935, 2873, 2810, 2769, 1694, 1619, 1599, 1514, 1493, 1463, 1444, 1392, 1298, 1046 cm^{-1}. ^{1}H-NMR ($CDCl_3$, 500 MHz): δ: 1.35 (dd, *J*= 9.3, 6.1 Hz, 8H, 4xCH_2), 1.47 (m, 4H, 2xCH_2), 1.66 (dt, *J*=16.4, 8.5 Hz, 2H, CH_2), 1.94–1.85 (m, 2H, CH_2), 2.30 (dd, *J*=15.0, 6.4 Hz, 8H, 4x*N*-CH_2), 2.48 (s, 12H, 6x*N*-CH_2), 3.48 (s, 4H, 2x*N*-CH_2), 3.64-3.59 (t, *J*=7.1 Hz, 2H, *N*-CH_2), 3.88–3.82 (m, 18H, 6x*O*-CH_3), 4.15 (t, *J*=7.2 Hz, 2H, *N*-CH_2), 6.39 (d, *J*=8.0 Hz, 1H, ArH), 6.61 (d, *J*=8.5 Hz, 2H, ArH), 6.75 (t, *J*=7.6 Hz, 1H, ArH), 6.96 (d, *J*=8.4 Hz, 2H, ArH), 7.12 (t, *J*=7.48 Hz, 1H, ArH), 7.30 (dd, *J*=7.2, 5.3, 1.4 Hz, 4H, ArH), 7.53 – 7.46 (m, 2H, ArH), 7.95 (s, 1H, ArH) ppm. ^{13}C-NMR (125 MHz, $CDCl_3$): δ : 26.7 (CH_2), 26.9 (CH_2), 27.0 (CH_2), 27.2 (CH_2), 27.3 (CH_2), 28.7 (CH_2), 29.7 (CH_2) 29.9 (CH_2), 38.3 (*N*-CH_2), 47.0 (*N*-CH_2), 52.7 (4x*N*-CH_2), 53.3 (4x*N*-CH_2), 53.4 (2x*O*-CH_3), 56.0 (*N*-CH_2), 56.5 (*N*-CH_2), 58.6 (*N*-CH_2), 58.7 (*N*-CH_2), 60.8 (2x*O*-CH_3), 61.2 (2x*O*-CH_3), 104.6 (Cq), 106.9 (2xCAr), 109.8 (CAr), 120.4 (CAr), 122.4 (CAr), 122.7 (CAr), 123.8 (Cq), 125.0 (Cq), 125.3 (2xCAr), 128.1 (Cq), 128.2 (2xCAr), 128.9 (CAr), 130.0 (2xCAr), 130.7 (2xCq), 131.6 (Cq), 133.6 (CAr), 136.7 (Cq), 142.3 (2xCq), 152.7 (2xCq), 152.9 (2xCq), 172.0 (C=O), 172.1 (C=O) ppm. HR-MS (ESI-QTOF): m/z: calculated: ($[C_{58}H_{76}N_6O_8]+2H)^{+2}$: 493.2935, found: 493.2945.

Synthesis of 1-(6-azidohexyl)-3-(1-(6-azidohexyl)-1*H*-indol-3-yl)-4-phenyl-1*H*-pyrrole-2,5-dione, (6b)

0.43 mmol (266 mg) of **5** was dissolved in 20 mL of dry DMF and taken into the reaction flask. 4.3 mmol (281.45 mg) of NaN_3 was dissolved in 2 mL of distilled water and added to the reaction flask. The reaction was stirred at 80°C overnight. According to TLC (1:1 ethyl acetate/n-hexane) result, it was observed that the starting material was finished. After the reaction came to room temperature, it was poured into ice water and extracted with ether-brine. The organic phase was dried with Na_2SO_4, filtered and the solvent was evaporated in vacuo. It was used in the next steps without purification. Orange oily substance; R_f: 0.8 (2:1 ethyl acetate/*n*-hexane); yield = 99%. FTIR (ATR): ν: 3052, 2933, 2857, 2089, 1693, 1619, 1514, 1491, 1392, 1371, 1242 cm^{-1}. HR-MS (ESI-QTOF): m/z: calculated: ($[C_{30}H_{34}N_8O_2]+H)^{+}$: 538.2801, found: 539.2873.

Synthesis of 1-(6-(4-(benzyloxy)-6,6-dimethyl-5-oxo-5,6-dihydro-1,2,3,4-tetrazin-1(4*H*)-yl)hexyl)-3-(1-(6-(4-(benzyloxy)-6,6-dimethyl-5-oxo-5,6-dihydro-1,2,3,4-tetrazin-1(4*H*)-yl)hexyl)-1*H*-indol-3-yl)-4-phenyl-1*H*-pyrrole-2,5-dione, (5e)

The resulting 2 mmol (544 mg) *N*-(benzyloxy)-2-bromo-2-methylpropanamide (**7**) and 1 mmol (534.64 mg) **6b** were taken into a Schlenk tube and 6 mmol (636 mg) anhydrous Na_2CO_3 was added. The air in the tube was removed by applying vacuum to the Schlenk tube, then the tube was filled with nitrogen gas. It was dissolved by adding 3 mL of 1,1,1,3,3,3-hexafluoro-2-propanol solvent with the help of an injector. The reaction was allowed to stir under a nitrogen atmosphere for 12 hours. The reaction was terminated by TLC control. The reaction mixture was filtered through celite,

and the solvent was removed in vacuo. It was purified by column chromatography in a 1:2 ethyl acetate/n-hexane system. Orange oily substance; R_f: 0.18 (1:2 ethyl acetate/*n*-hexane); yield = 48%. FTIR (ATR): ν: 3032, 2936, 2861, 1695, 1620, 1515, 1491, 1392, 1366, 1236, 1080 cm^{-1}. ^{1}H-NMR ($CDCl_3$, 500 MHz): δ : 1.30 (d, *J*= 2.6 Hz, 12H, 4xCH_3), 1.40 (m, 8H, 4xCH_2), 1.70 (m, 6H, 3xCH_2), 1.96–1.89 (m, 2H, CH_2), 3.32 (dd, *J*=13.9, 6.5 Hz, 4H, 2x*N*-CH_2), 3.66 (t, *J*=7.1 Hz, 2H, *N*-CH_2), 4.19 (t, *J*=7.1 Hz, 2H, *N*-CH_2), 5.13 (s, 4H, 2x*O*-CH_2), 6.43 (d, J=8.1 Hz, 1H, ArH), 6.78 (t, *J*=7.2 Hz, 1H, ArH), 7.15 (t, *J*=7.7 Hz, 1H, ArH), 7.37 – 7.30 (m, 10H, ArH), 7.47 (dd, *J*=7.3, 2.0 Hz, 4H, ArH), 7.52 (dd, *J*=7.4, 2.2 Hz, 2H, ArH), 7.98 (s, 1H, ArH) ppm. APT (125 MHz, $CDCl_3$): δ : 20.5 (4xCH_3), 26.2 (CH_2), 26.5 (CH_2), 26.6 (CH_2), 28.6 (CH_2), 29.5 (CH_2), 29.6 (CH_2), 29.8 (CH_2), 38.1 (CH_2), 46.8 (*N*-CH_2), 48.8 (*N*-CH_2), 59.8 (*N*-CH_2), 61.8 (*N*-CH_2), 71.1 (*O*-CH_2), 71.6 (*O*-CH_2), 78.4 (2xCq), 109.7 (CAr), 120.5 (CAr), 122.4 (CAr), 122.7 (CAr), 125.0 (Cq), 125.8 (Cq), 128.2 (2xCAr), 128.4 (4xCAr), 128.9 (CAr), 129.0 (2xCAr), 129.7 (2xCAr), 129.8 (2xCAr), 129.9 (2xCAr), 130.6 (Cq), 131.5 (Cq), 133.5 (CAr), 133.8 (Cq), 133.9 (2xCq), 136.6 (Cq), 162.0 (C=O), 162.1 (C=O), 171.9 (C=O), 172.0 (C=O) ppm. HR-MS (ESI-QTOF): m/z: calculated: $([C_{52}H_{60}N_{10}O_6]+H)^+$: 921.4776, found: 921.4742.

Synthesis of 3-phenyl-1-(6-(4-phenyl-1*H*-1,2,3-triazol-1-yl)hexyl)-4-(1-(6-(4-phenyl-1*H*-1,2,3-triazol-1-yl)hexyl)-1*H*-indol-3-yl)-1*H*-pyrrole-2,5-dione, (5f)

0.22 mmol (118 mg) of **6b** was weighed into a single neck balloon. 0.5 mmol (51.06 mg) phenyl acetylene, 0.005 mmol (1.25 mg) $CuSO_4.5H_2O$, 0.01 mmol (1.98 mg) sodium ascorbate, 0.05 mmol (6.07 mg) benzoic acid and 1:2 (0.2:0.4 mL) *t*-BuOH:H_2O added. The reaction was stirred at room temperature overnight. At the end of the night, TLC control was performed, and it was observed that the starting materials were finished. The reaction was extracted with dichloromethane. After drying with Na_2SO_4, the solvent was removed. Purification was carried out by column chromatography in a 2:1 ethyl acetate/n-hexane system. Orange oily substance; Rf: 0.46 (2:1 ethyl acetate/*n*-hexane); yield = 68%. FTIR (ATR): ν: 3053, 2938, 2859, 1692, 1619, 1514, 1490, 1436, 1392, 1360, 1298 cm^{-1}. ^{1}H-NMR ($CDCl_3$, 500 MHz): δ : 1.39 (m, 8H, 4xCH_2), 1.70–1.65 (m, 2H, CH_2), 1.95–1.88 (m, 6H, 3xCH_2), 3.63 (t, *J*=7.1 Hz, 2H, *N*-CH_2), 4.15 (t, *J*=7.0 Hz, 2H, *N*-CH_2), 4.34 (dd, *J*=15.2, 7.4 Hz, 4H, 2x*N*-CH_2), 6.41 (d, *J*=8.1 Hz, 1H, ArH), 6.77 (t, *J*=7.4 Hz, 1H, ArH), 7.12 (t, *J*=7.9 Hz, 1H, ArH), 7.32–7.28 (m, 6H, ArH), 7.39 (td, *J*=7.6, 3.5 Hz, 4H, ArH), 7.50 (dd, *J*=7.5, 2.0 Hz, 2H, ArH), 7.70 (s, 1H, ArH), 7.75 (s, 1H, ArH), 7.81 (dd, J=7.6, 6.5 Hz, 4H, ArH), 7.94 (s, 1H, ArH) ppm. APT (125 MHz, $CDCl_3$): δ : 26.0 (2xCH_2), 26.2 (CH_2), 26.3 (CH_2), 28.4 (CH_2), 29.5 (CH_2), 30.0 (CH_2), 30.1 (CH_2), 37.98 (CH_2), 46.7 (CH_2), 50.1 (CH_2), 50.2 (CH_2), 104.6 (Cq), 119.5 (CAr), 120.5 (CAr), 122.4 (CAr), 122.7 (CAr), 125.0 (Cq), 125.6 (3xCAr), 125.7 (2xCAr), 128.0 (CAr), 128.1 (2xCAr), 128.2 (2xCAr), 128.8 (CAr), 128.6 (3xCAr), 128.9 (CAr), 129.9 (2xCAr), 130.6 (2xCq), 130.7 (Cq), 130.8 (Cq), 131.5 (Cq), 133.5 (CAr), 136.6 (Cq), 147.7 (Cq), 147.8 (Cq), 171.9 (C=O), 172.0 (C=O) ppm. HR-MS (ESI-QTOF): m/z: calculated: $([C_{46}H_{46}N_8O_2]+H)^+$: 743.3822, found: 743.3817.

Synthesis of *N*-(Benzyloxy)-2-bromo-2-methylpropanamide, (7)[4]

5 mmol (798.05 mg) of *O*-benzylhydroxylamine hydrochloride was taken into a two-necked flask. 5 mmol (505.95 mg) of anhydrous triethylamine was added to it and after adding anhydrous CH_2Cl_2 to dissolve it, it was mixed with a magnetic stirrer at 0°C. After the reaction reached 0°C, 5 mmol (1149.5 mg) of 2-bromoisobutyryl bromide was added dropwise with the help of an injector. After TLC control (1:1 ethyl acetate/n-hexane) was performed, extraction with CH_2Cl_2 and water was applied. Molecular sieve was used for drying and the solvent was evaporated. Petroleum ether and ether were added to the resulting oily substance, and it was precipitated in an ultrasonic bath. White solid, m.p. 88.6-91.1°C, R_f: 0.2 (1:3 ethyl acetate/*n*-hexane), yield: 87%. FTIR (ATR): ν: 3192, 3036, 2956, 2890, 1651, 1497, 1469, 1454, 1112, 1031, 1004 cm^{-1}. ^{1}H-NMR (500 MHz, $CDCl_3$): δ 9.02 (brs, 1H), 7.37-7.31 (m, 5H), 4.87 (s, 2H), and 1.86 (s, 6H) ppm.

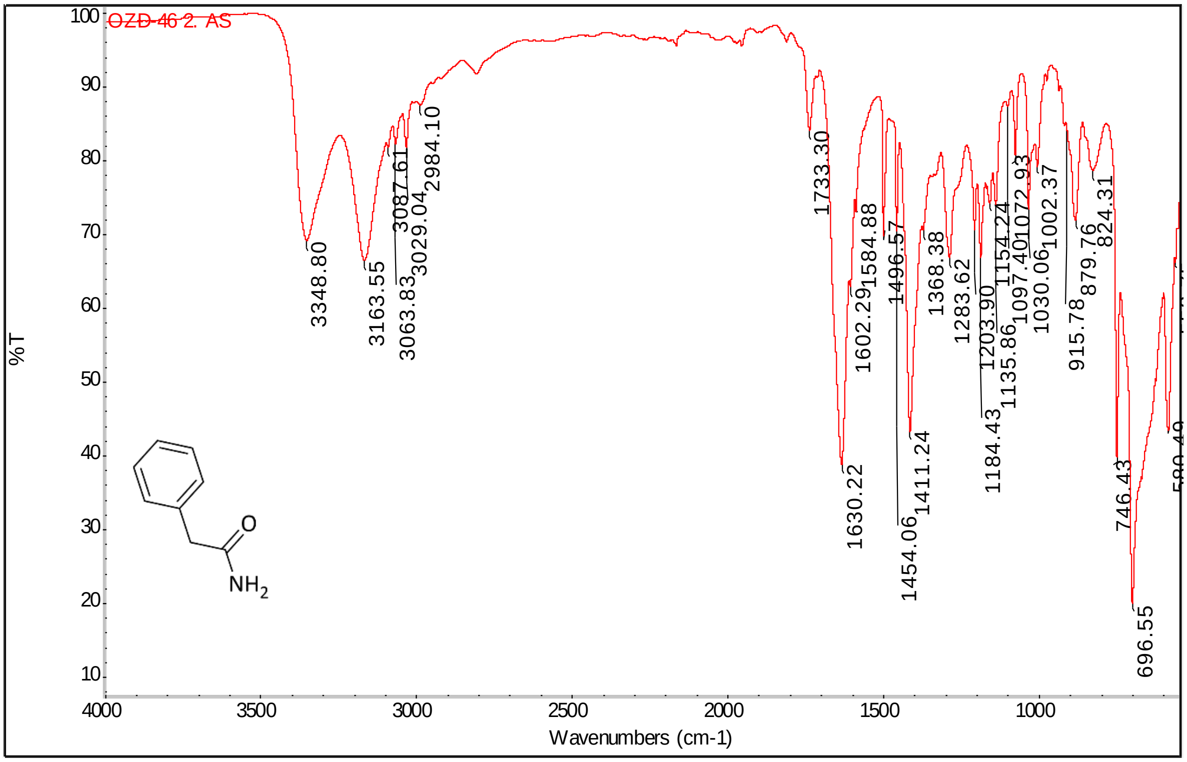

Figure S1. FTIR spectrum of compound **1** (ATR)

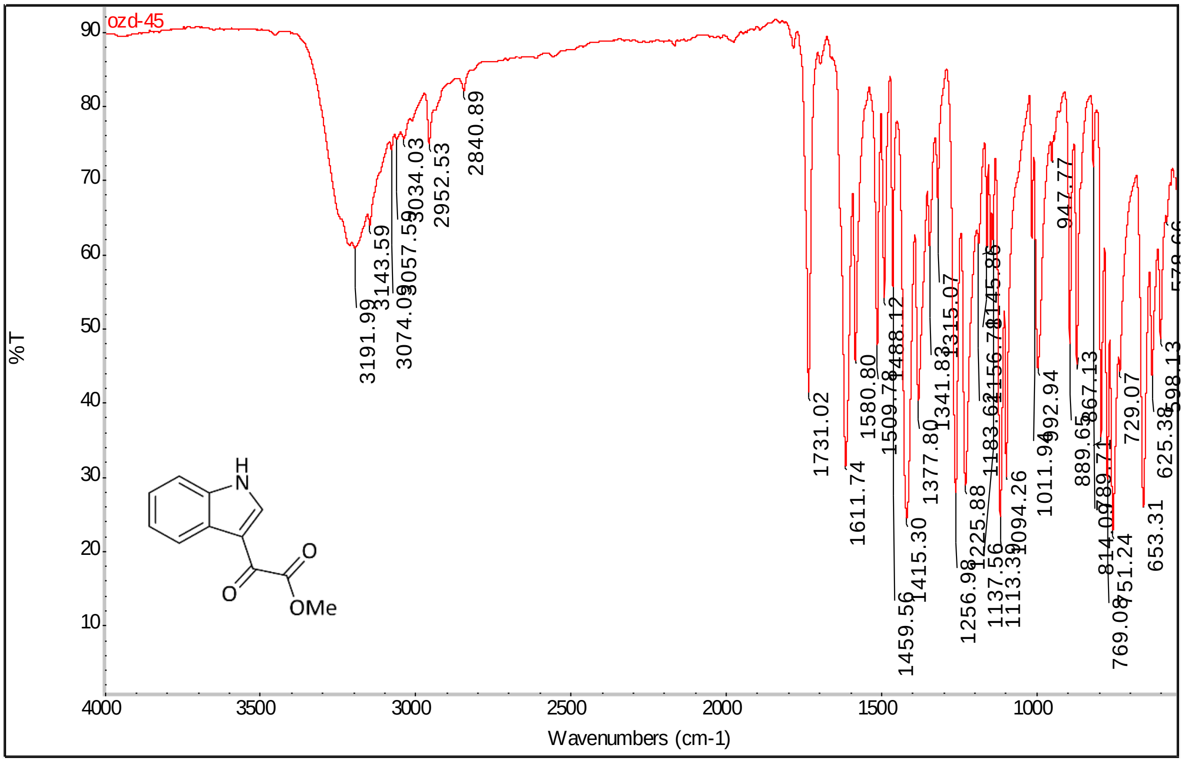

Figure S2. FTIR spectrum of compound **2** (ATR)

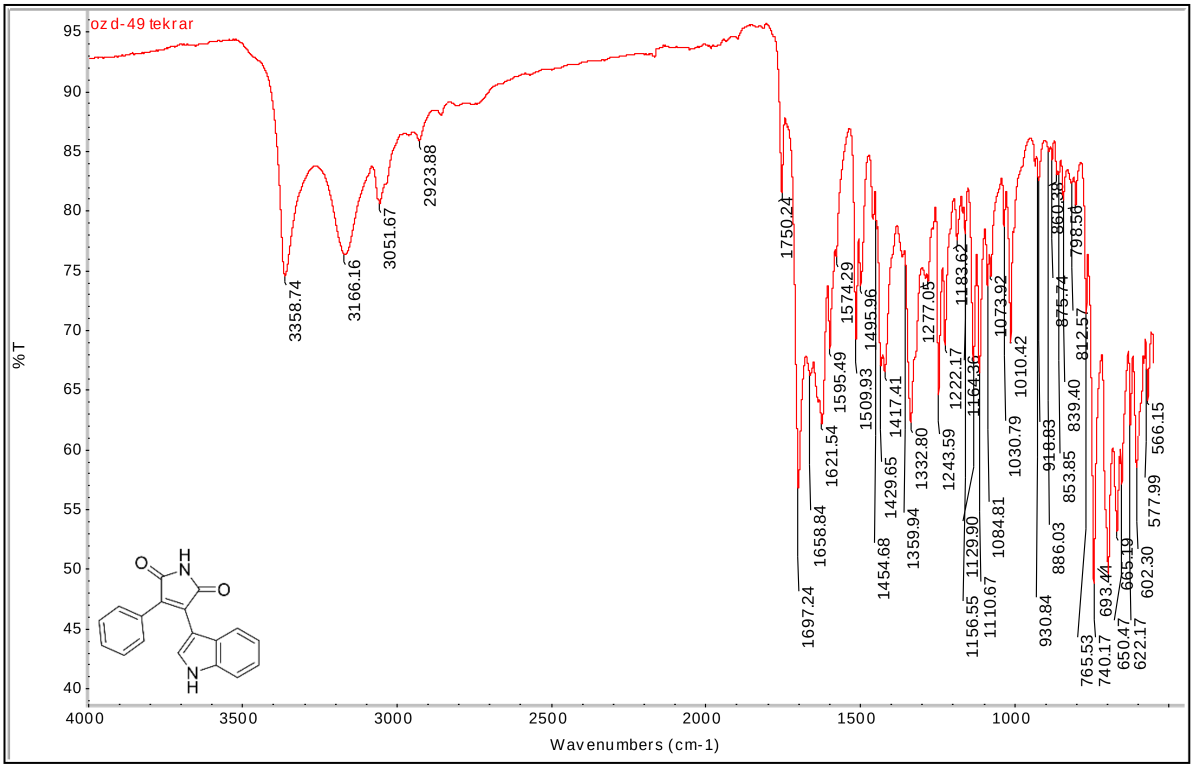

Figure S3. FTIR spectrum of compound **3** (ATR)

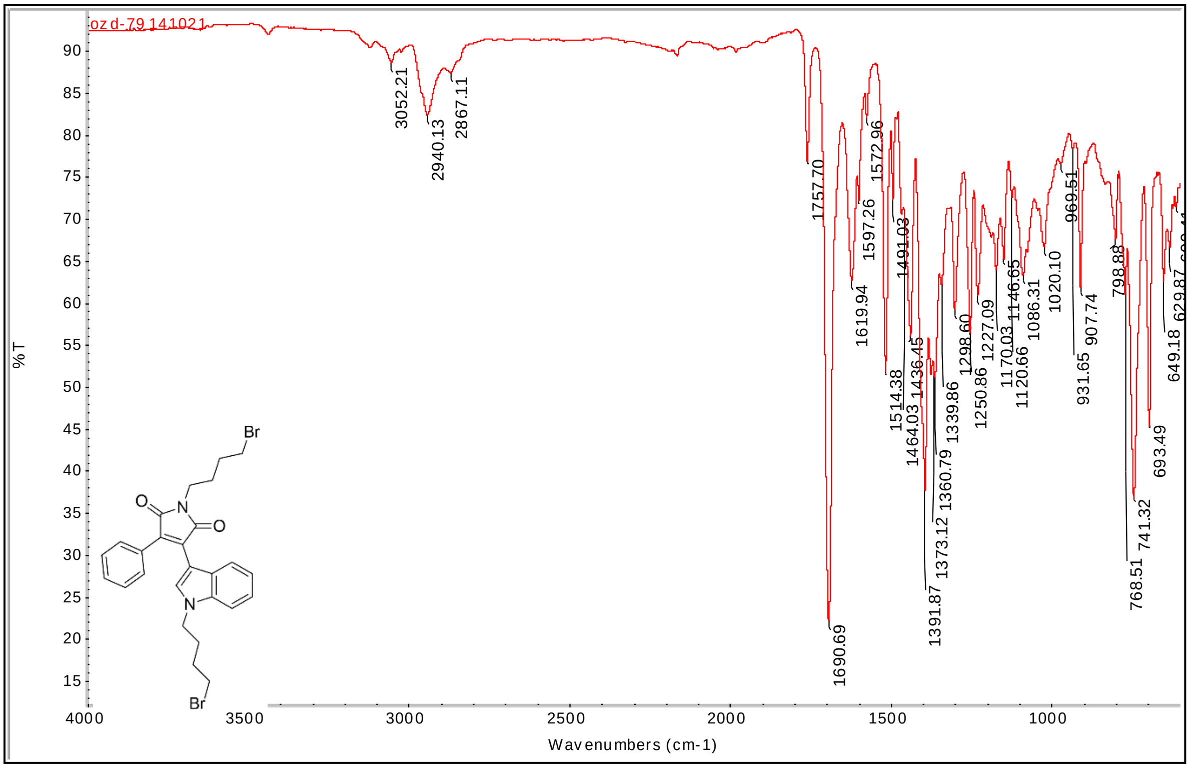

Figure S4. FTIR spectrum of compound **4** (ATR)

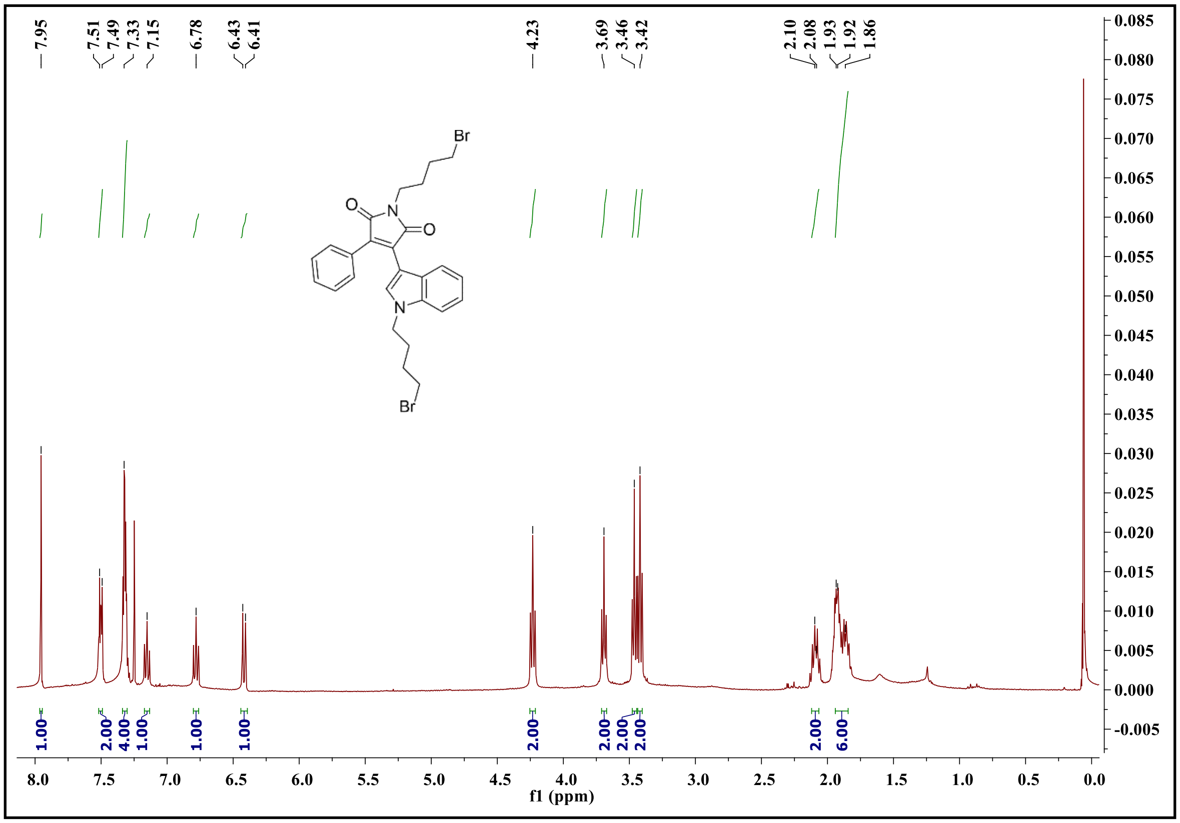

Figure S5. ^{1}H-NMR spectrum of compound **4** ($CDCl_3$)

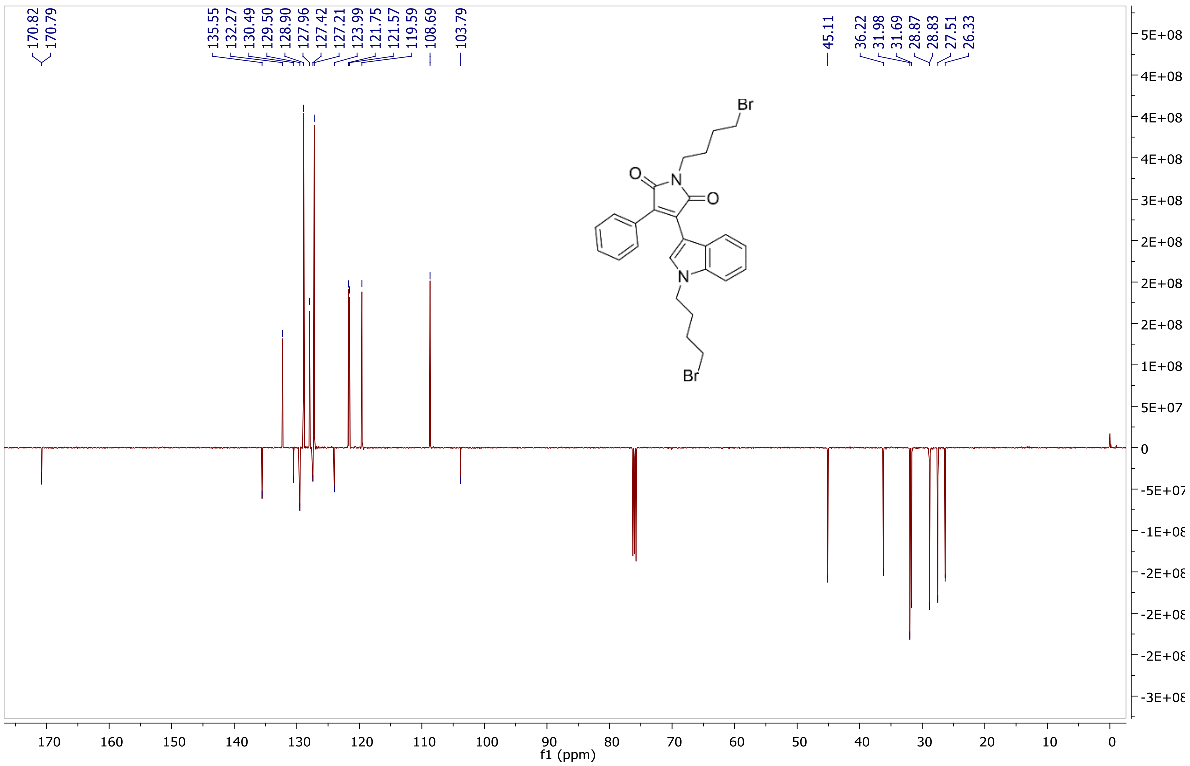

Figure S6. APT NMR spectrum of compound **4** ($CDCl_3$)

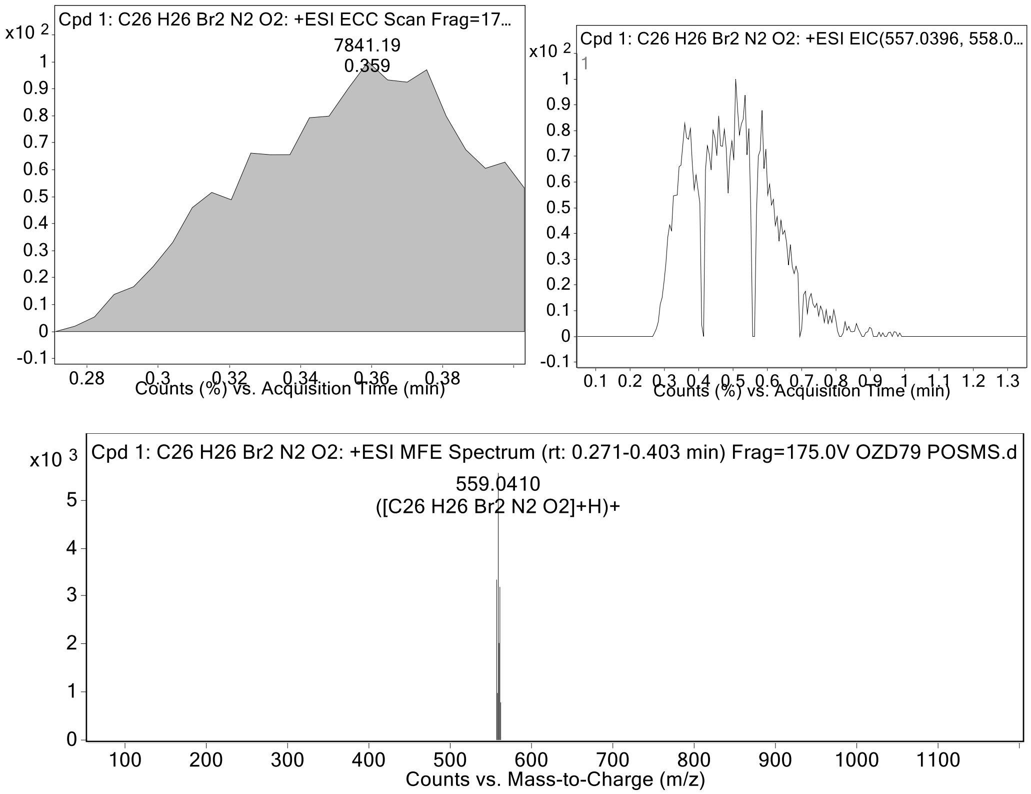

Figure S7. HR-MS (QTOF) spectrum of compound **4**

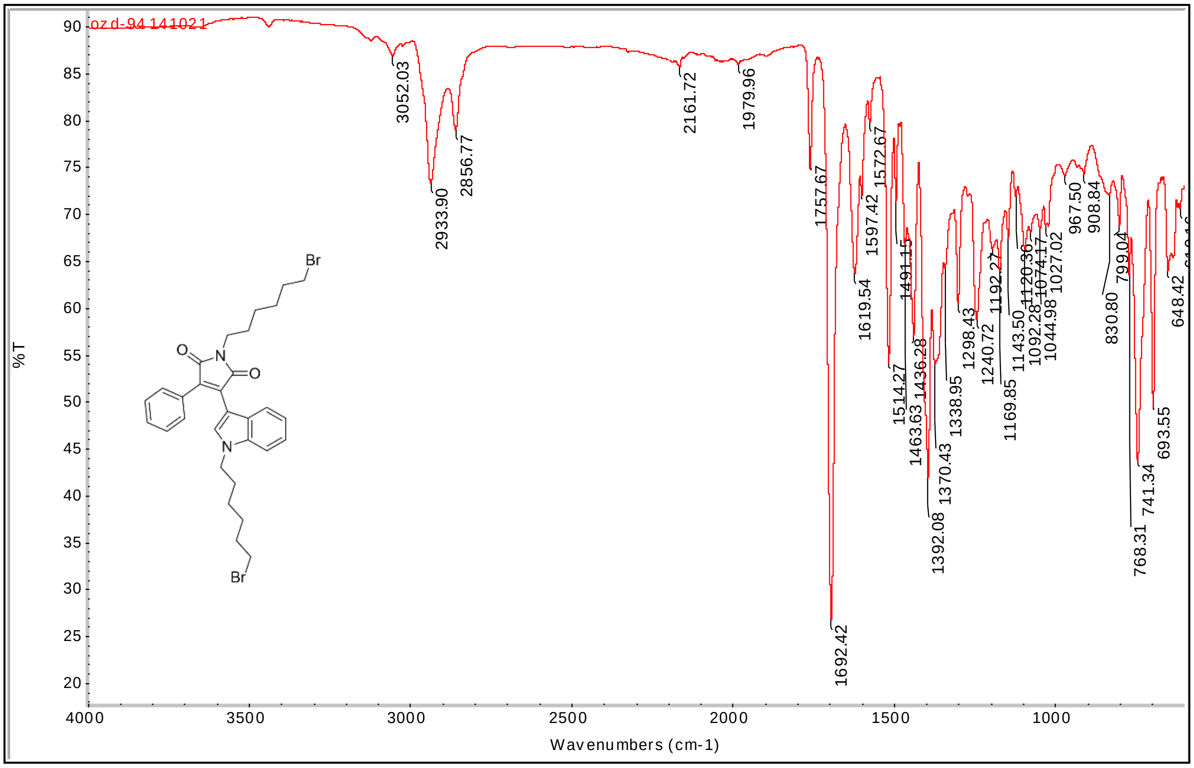

Figure S8. FTIR spectrum of compound **5** (ATR)

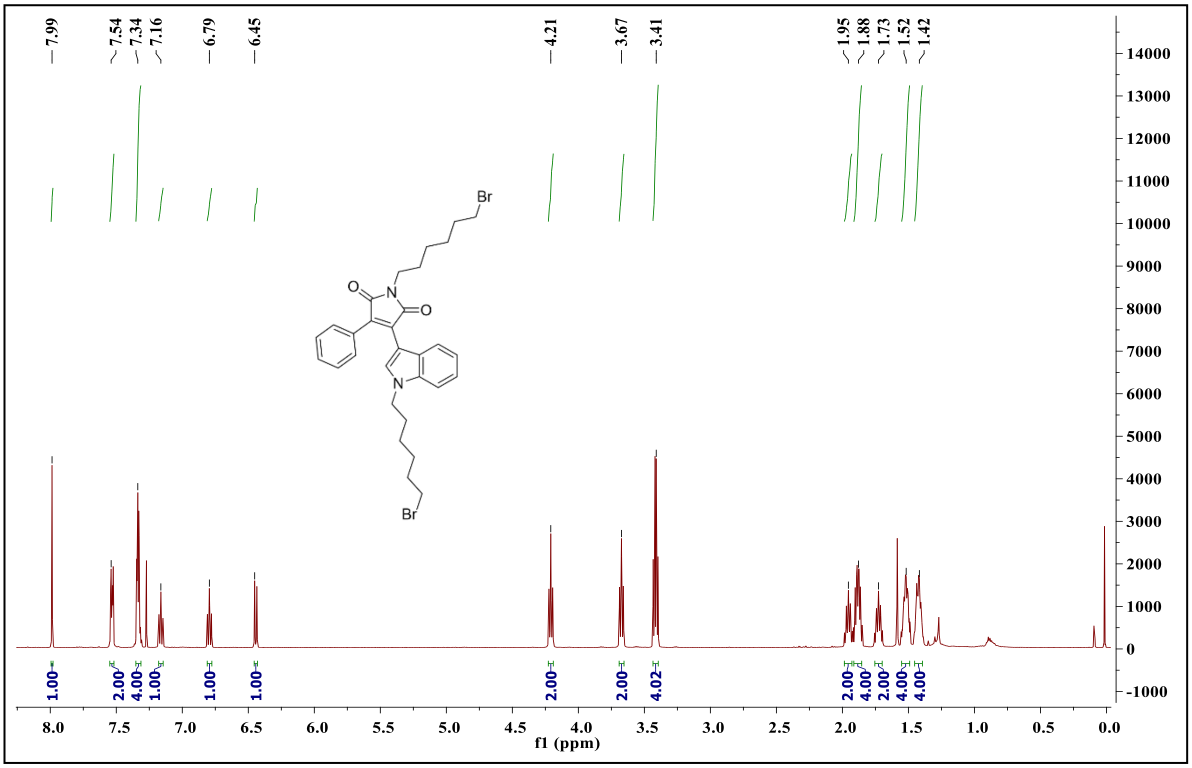

Figure S9. ^{1}H-NMR spectrum of compound **5** ($CDCl_3$)

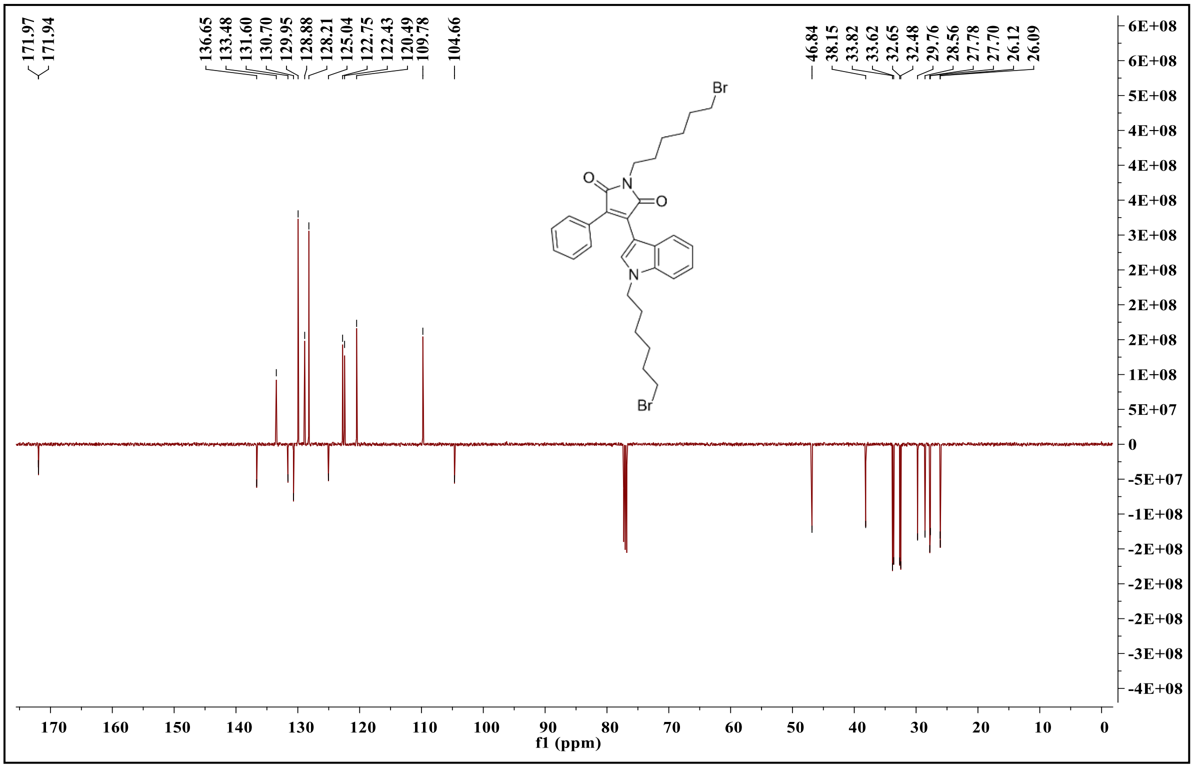

Figure S10. APT NMR spectrum of compound **5** ($CDCl_3$)

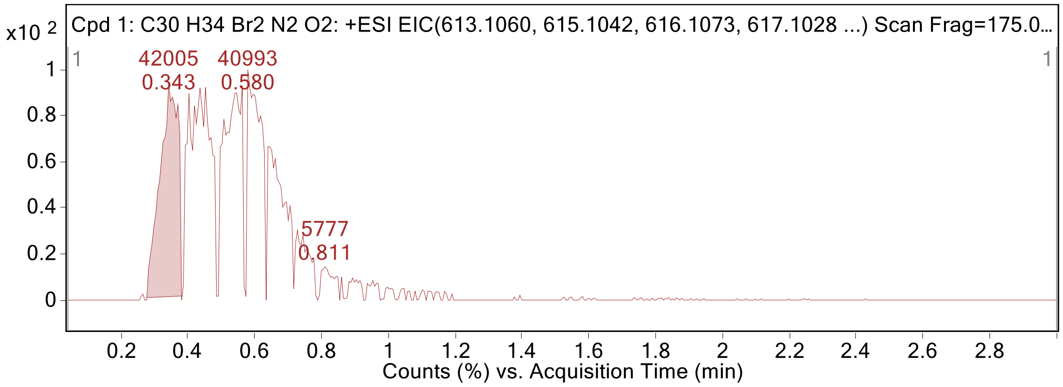

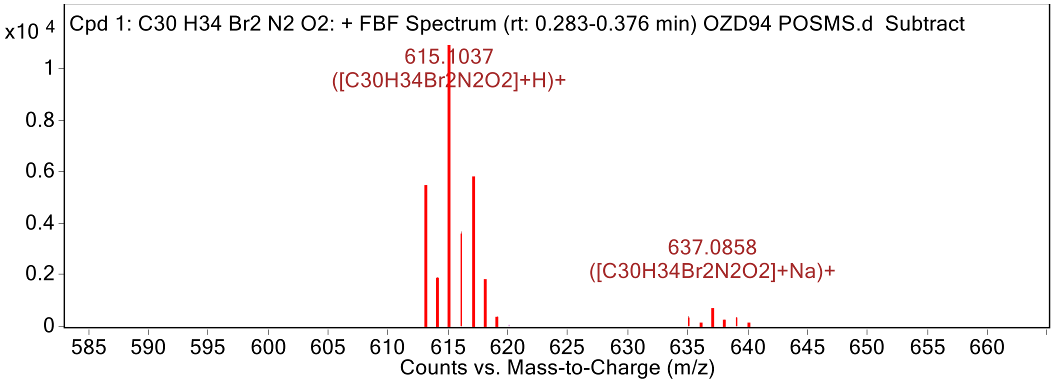

Figure S11. HR-MS (QTOF) spectrum of compound **5**

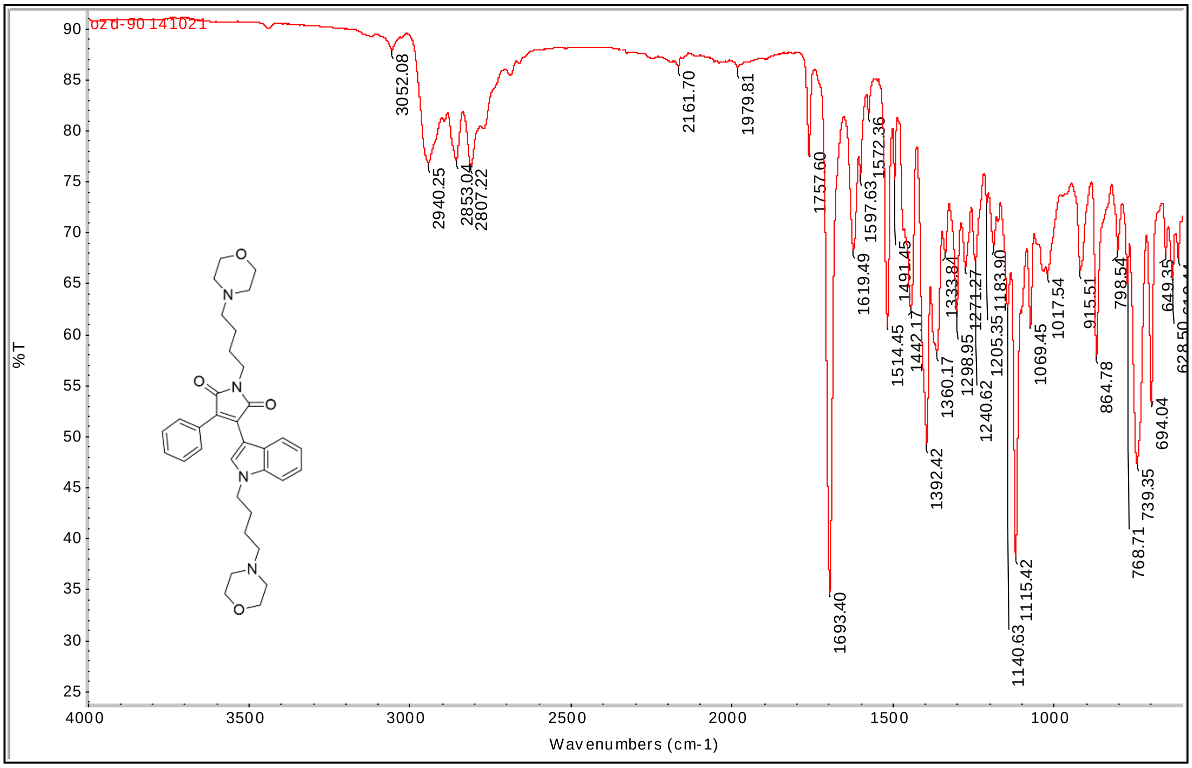

Figure S12. FTIR spectrum of compound **4a** (ATR)

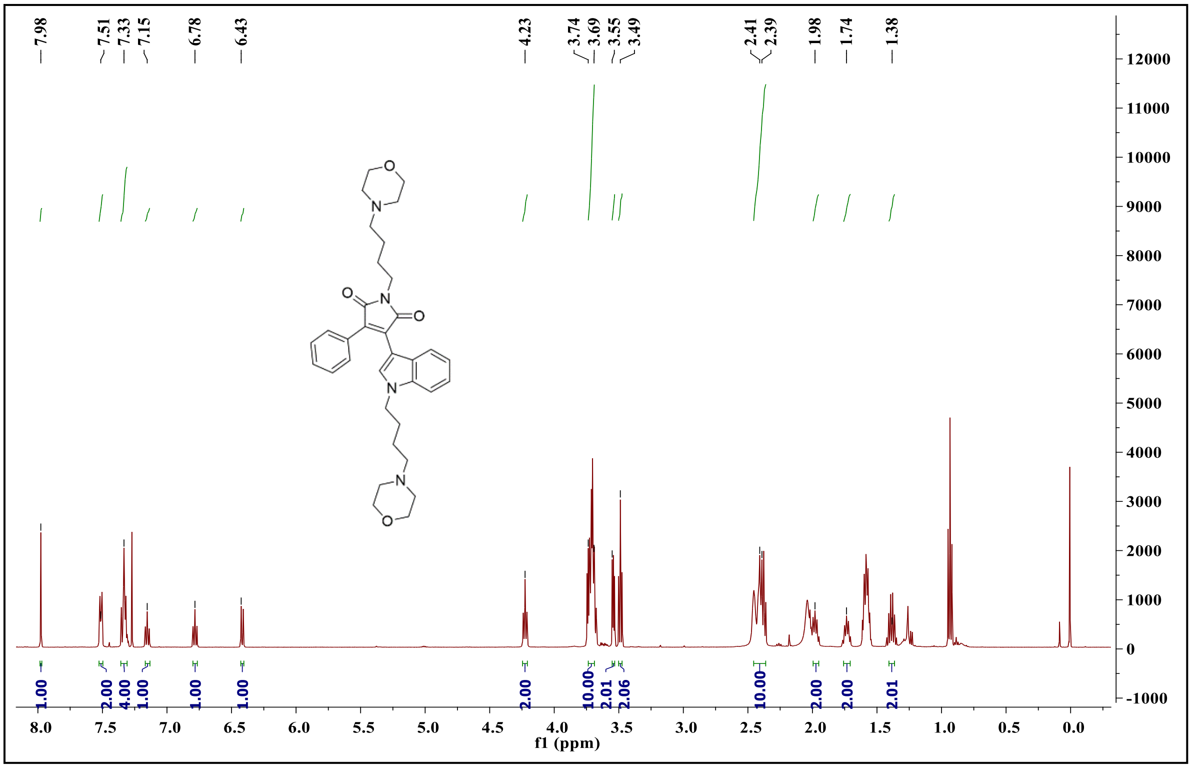

Figure S13. ^{1}H-NMR spectrum of compound **4a** ($CDCl_3$)

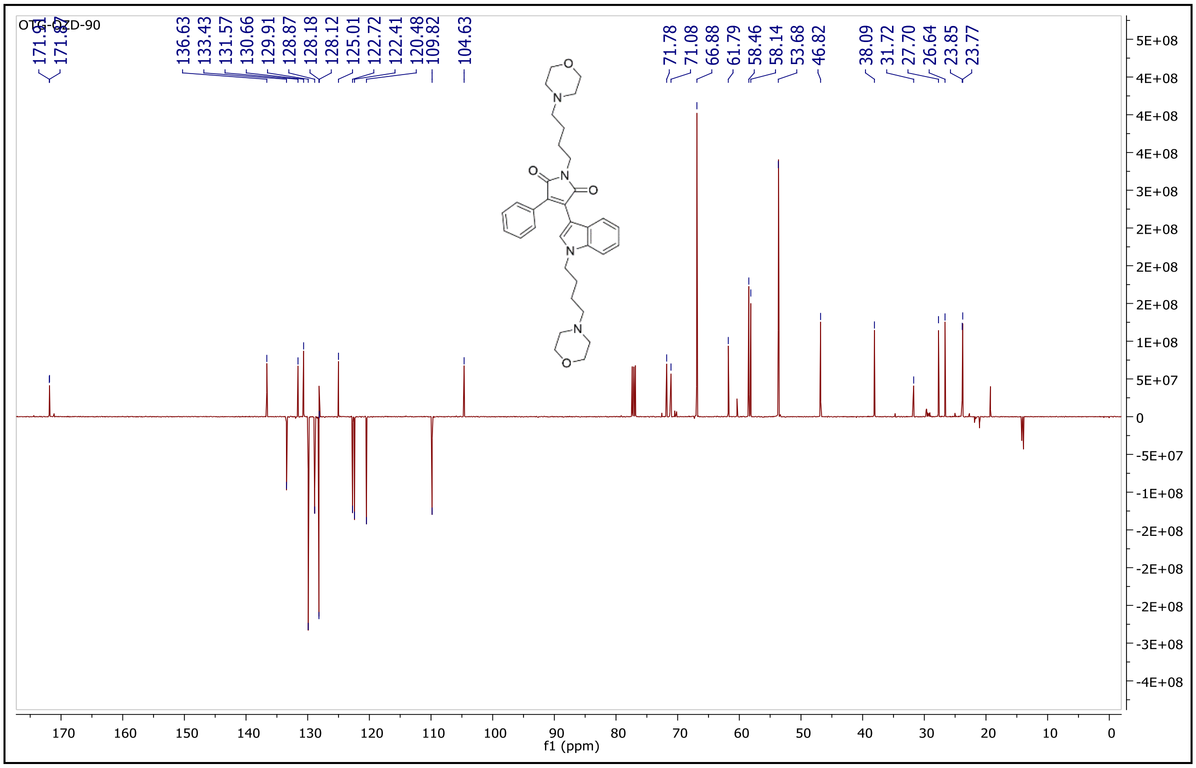

Figure S.14 APT NMR spectrum of compound **4a** ($CDCl_3$)

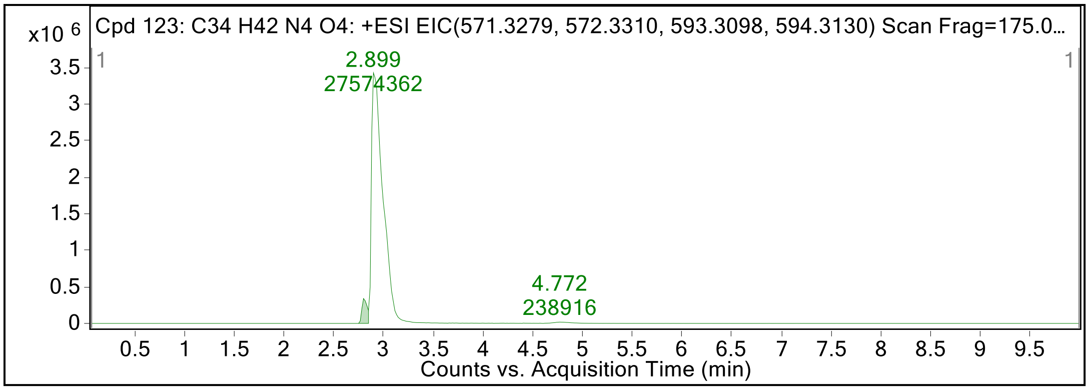

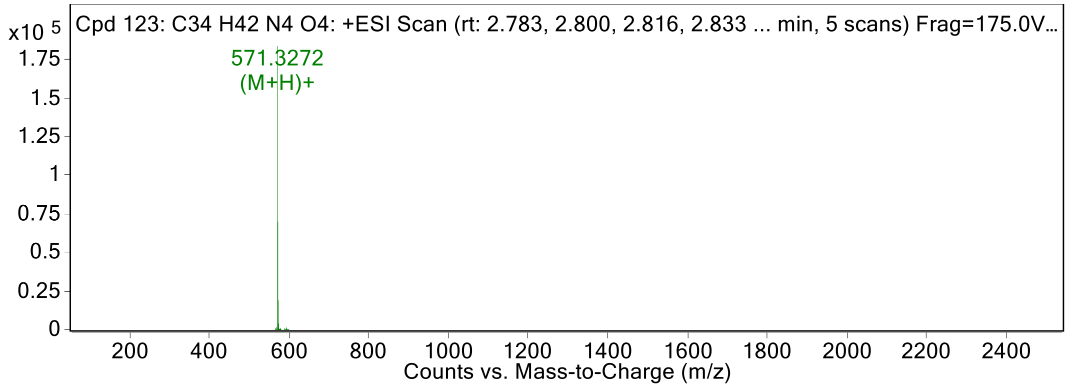

Figure S15. HR-MS (QTOF) spectrum of compound **4a**

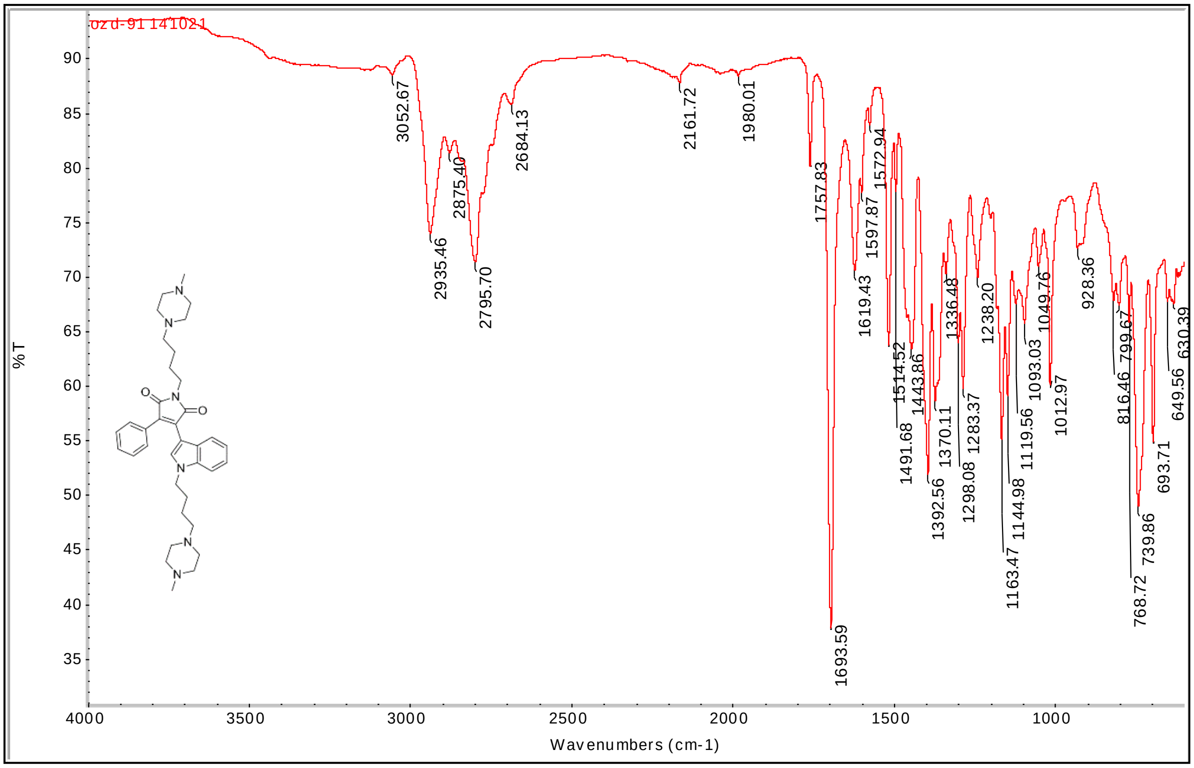

Figure S16. FTIR spectrum of compound **4b** (ATR)

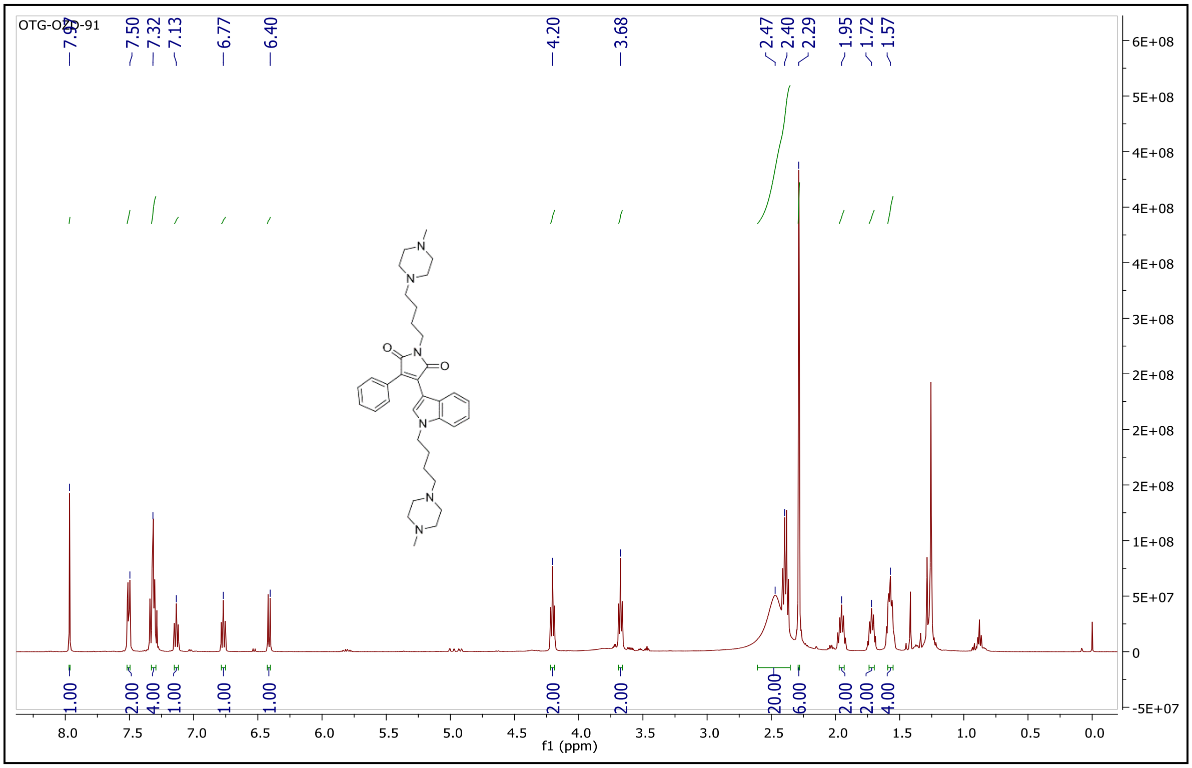

Figure S17. ^{1}H-NMR spectrum of compound **4b** ($CDCl_3$)

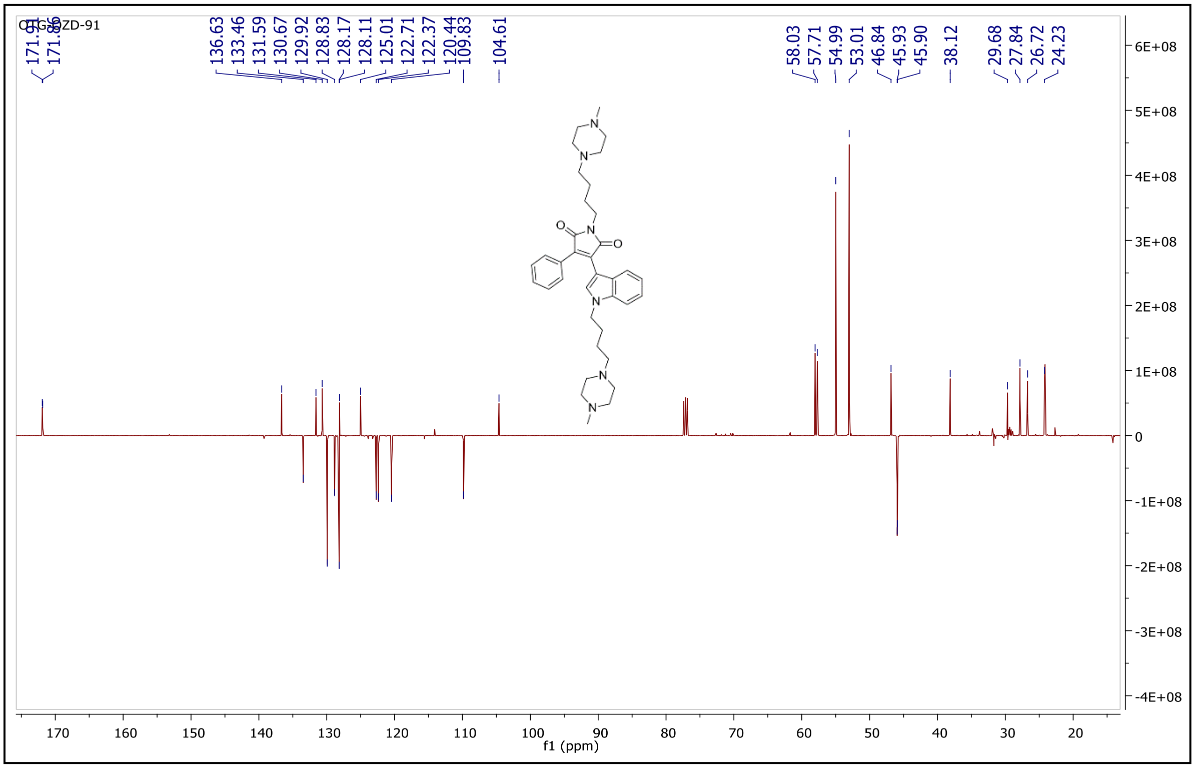

Figure S18. APT NMR spectrum of compound **4b** ($CDCl_3$)

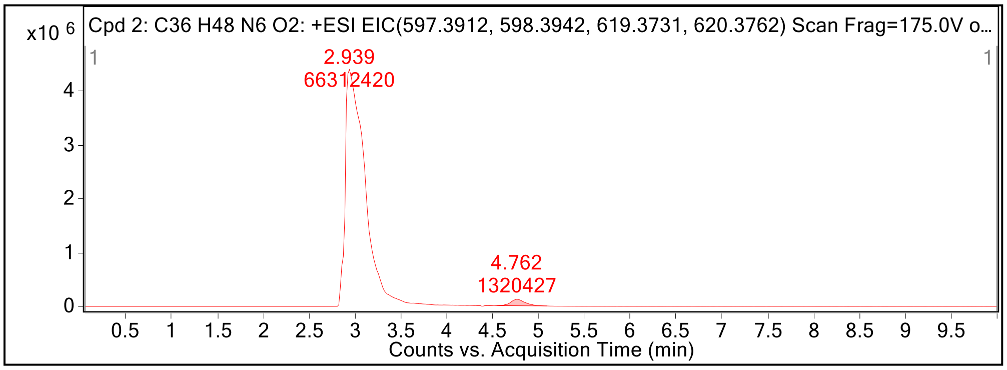

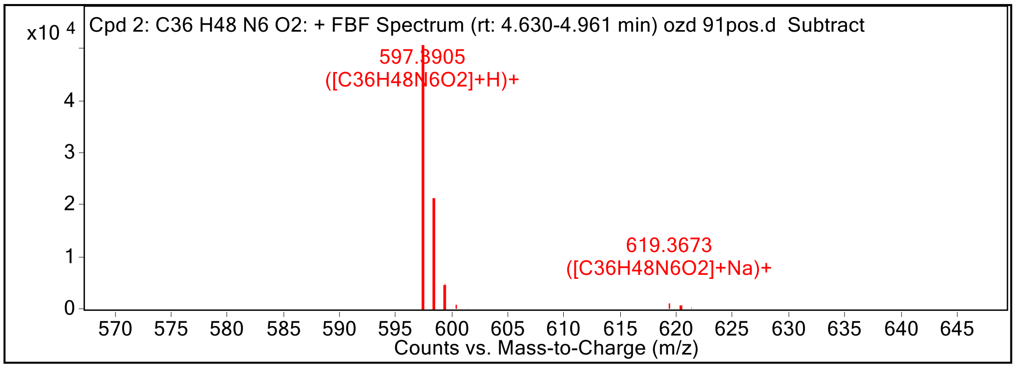

Figure S19. HR-MS (QTOF) spectrum of compound **4b**

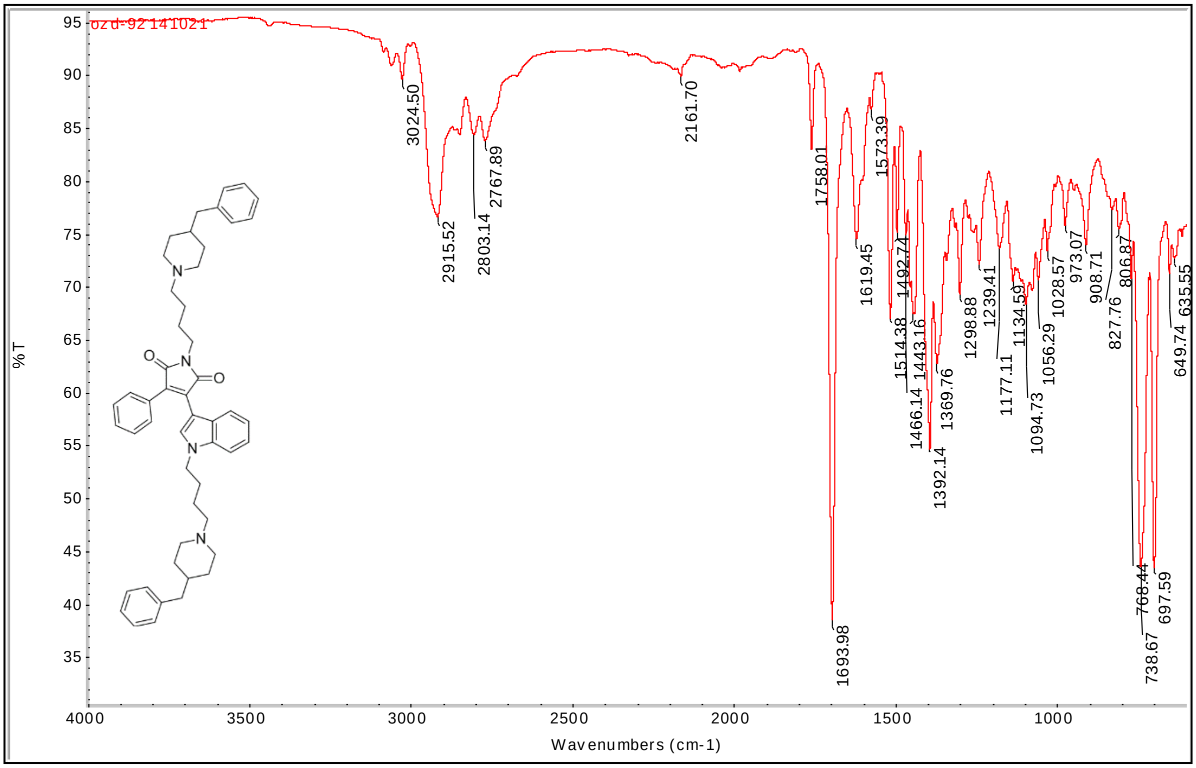

Figure S20. FTIR spectrum of compound **4c** (ATR)

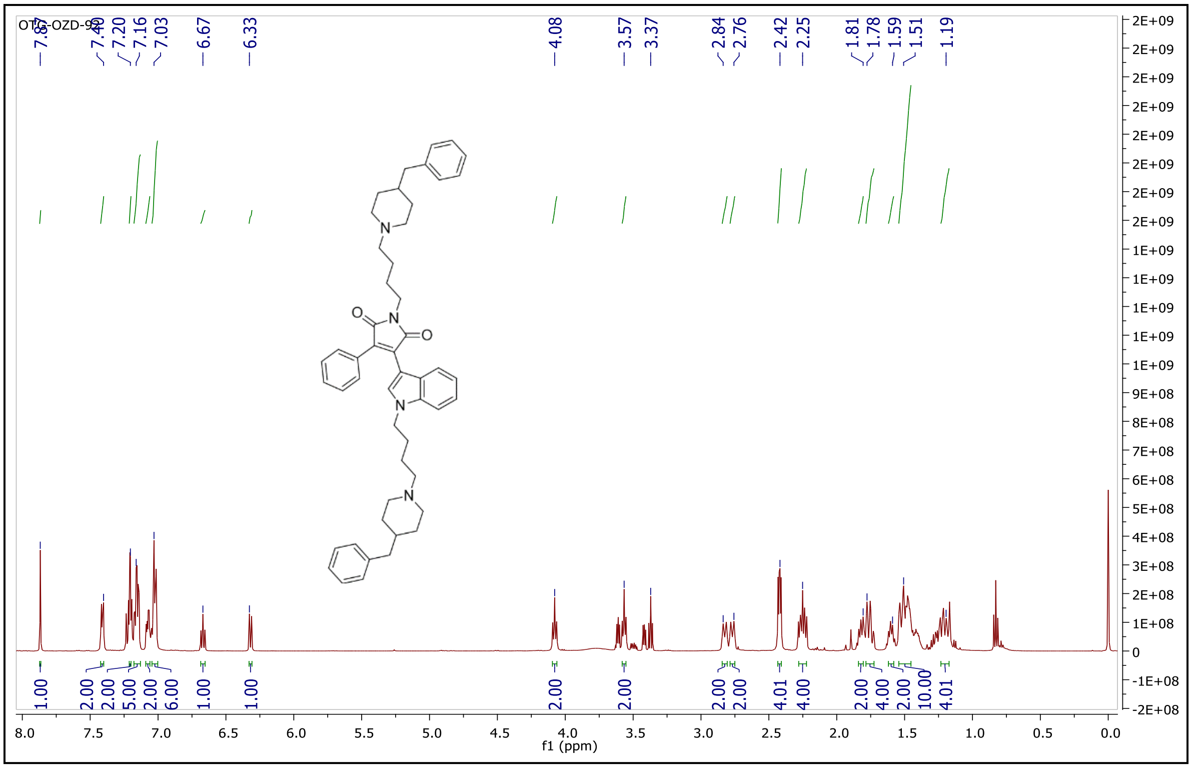

Figure S21. ^{1}H-NMR spectrum of compound **4c** ($CDCl_3$)

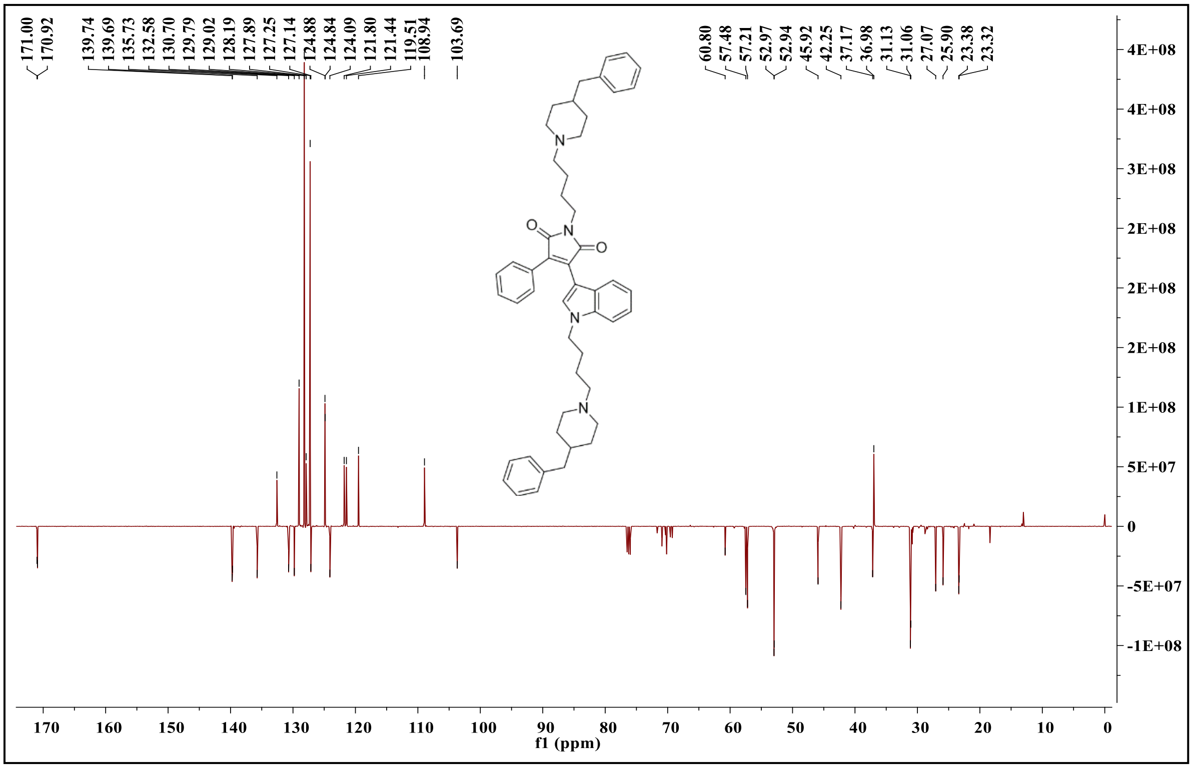

Figure S22. APT NMR spectrum of compound **4c** ($CDCl_3$)

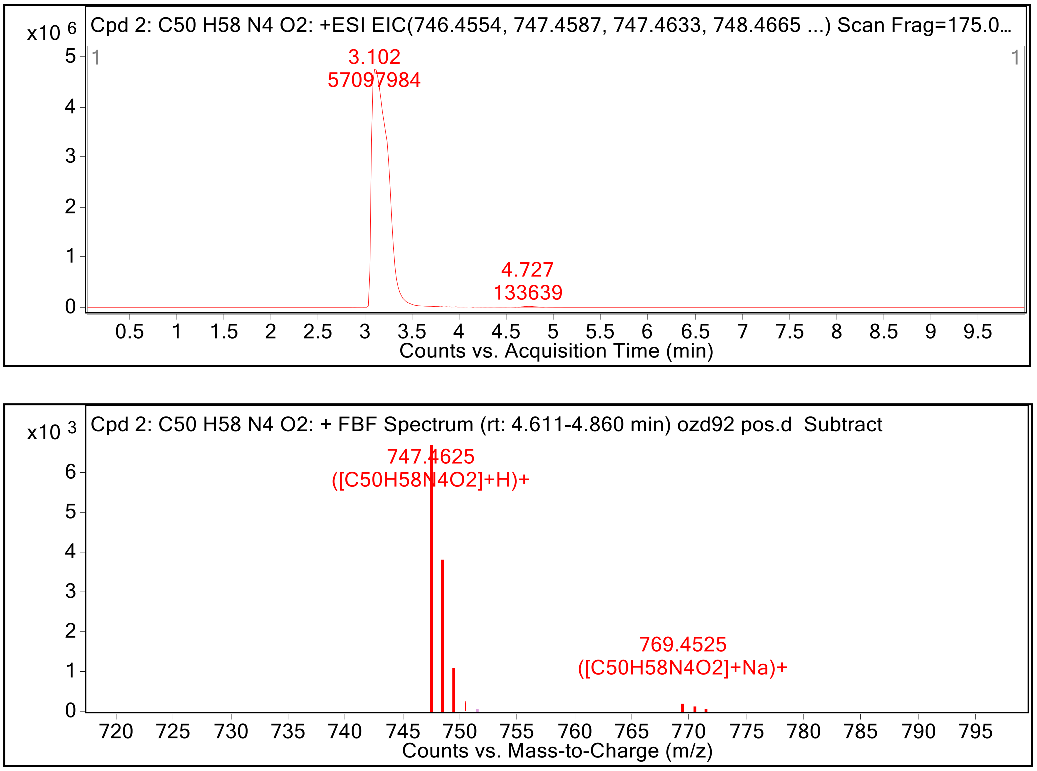

Figure S23. HR-MS (QTOF) spectrum of compound **4c**

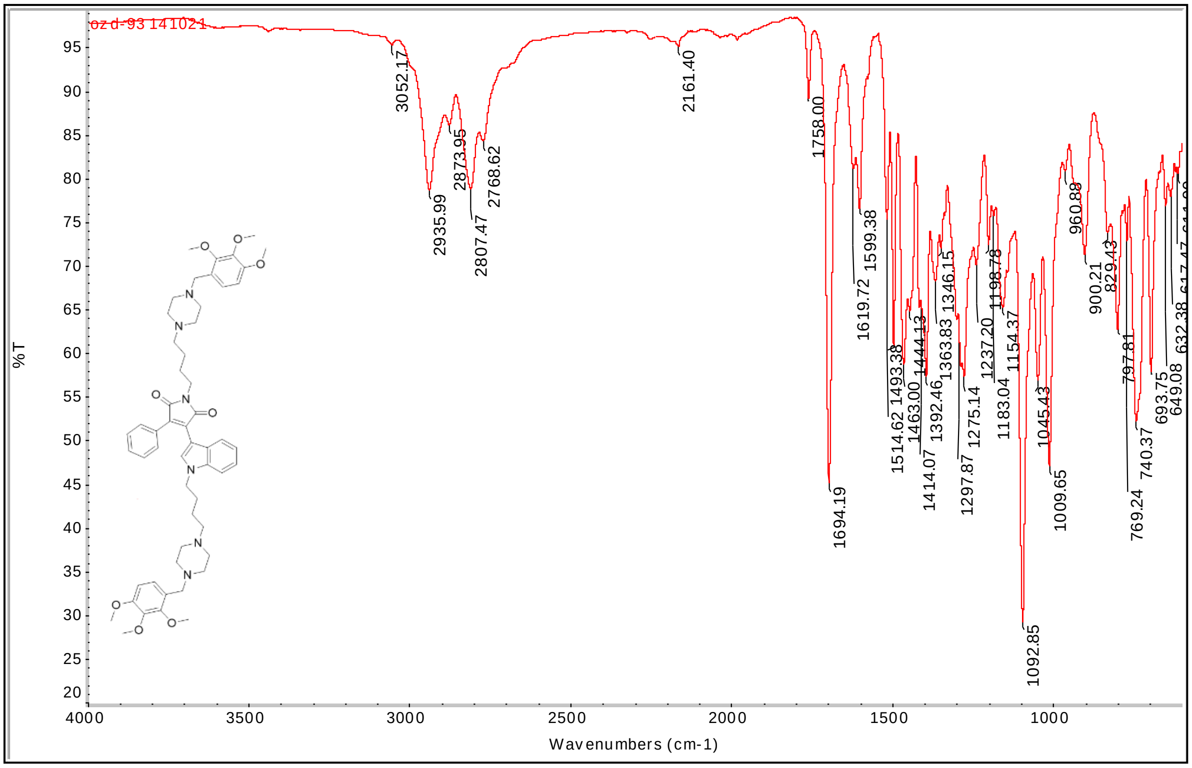

Figure S24. FTIR spectrum of compound **4d** (ATR)

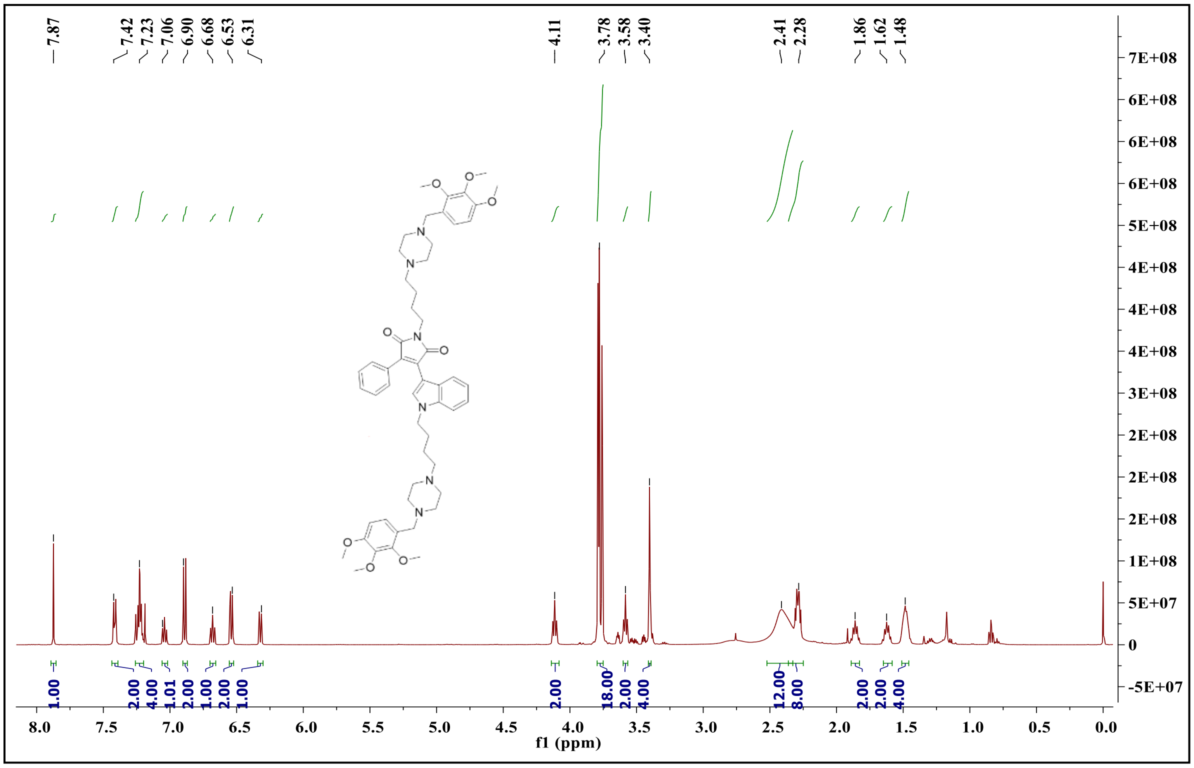

Figure S25. ^{1}H-NMR spectrum of compound **4d** ($CDCl_3$)

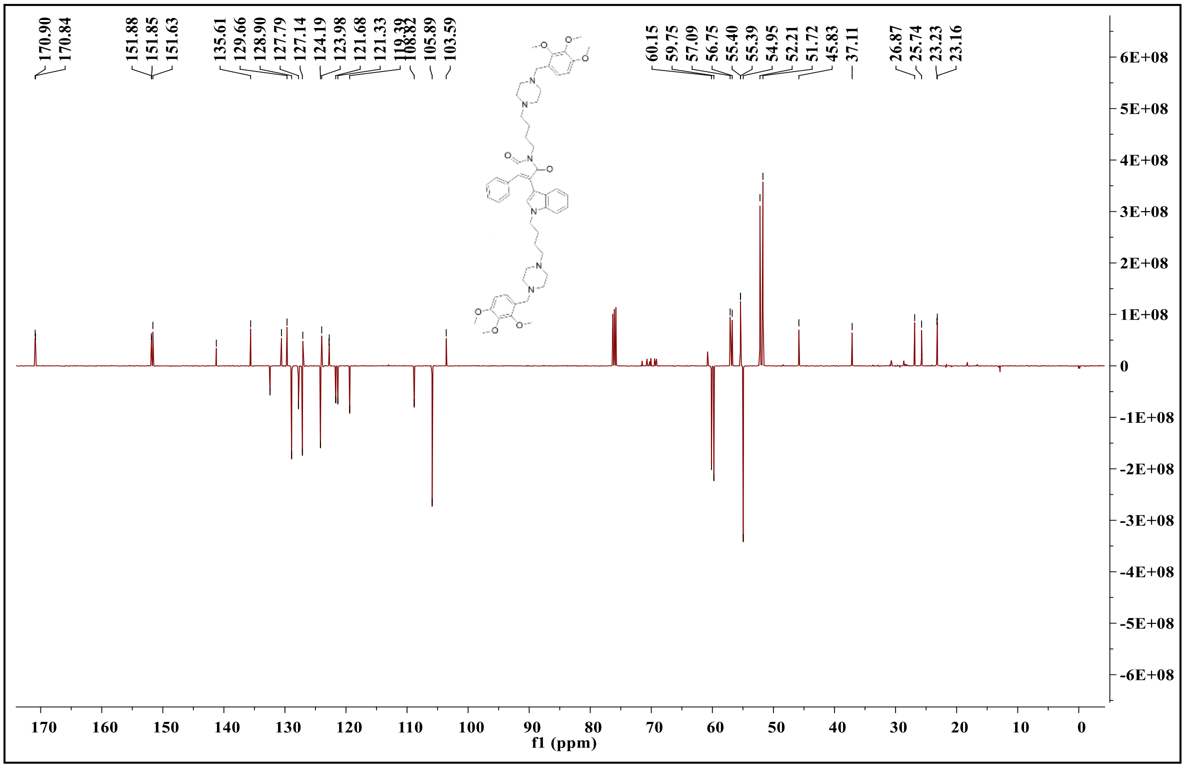

Figure S26. APT NMR spectrum of compound **4d** ($CDCl_3$)

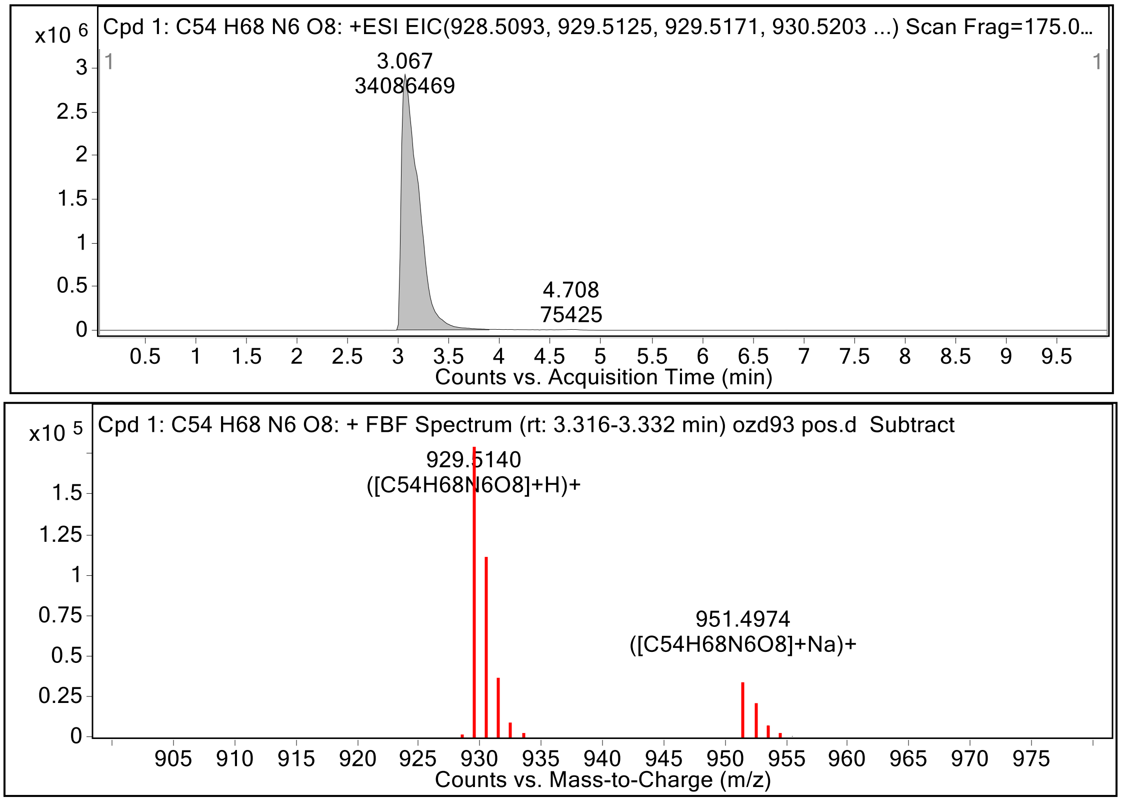

Figure S27. HR-MS (QTOF) spectrum of compound **4d**

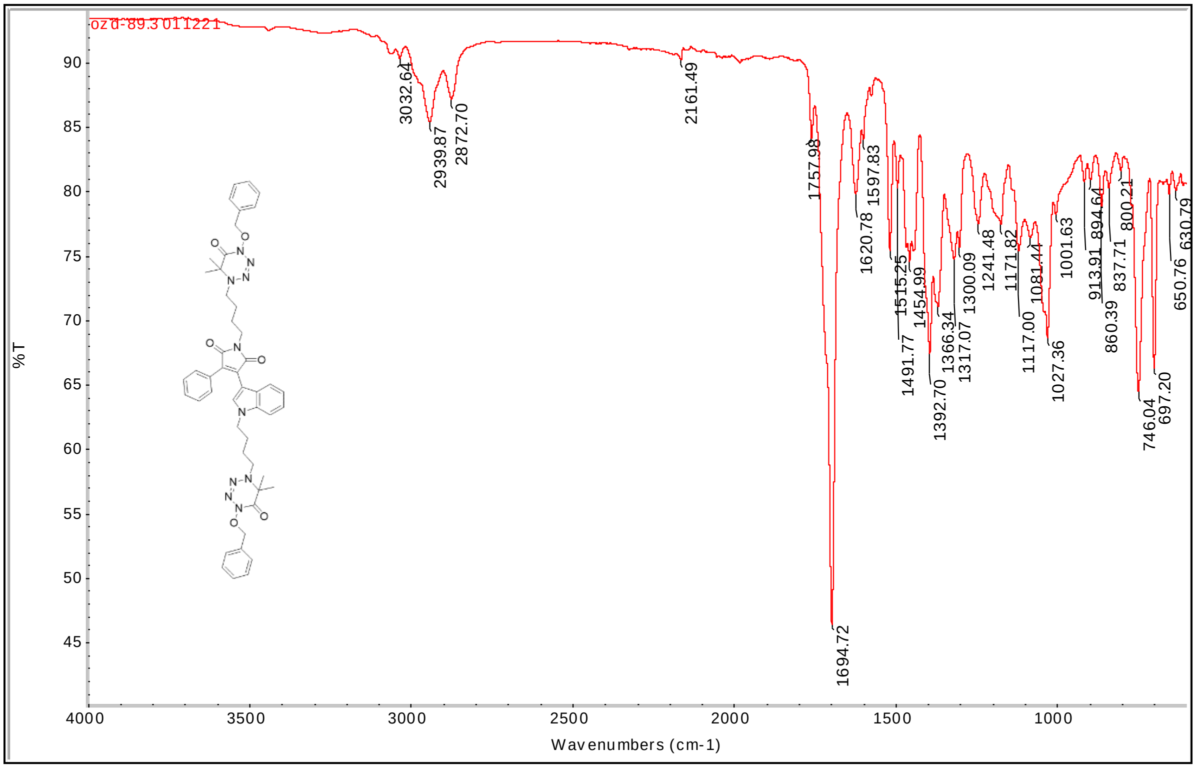

Figure S28. FTIR spectrum of compound **4e** (ATR)

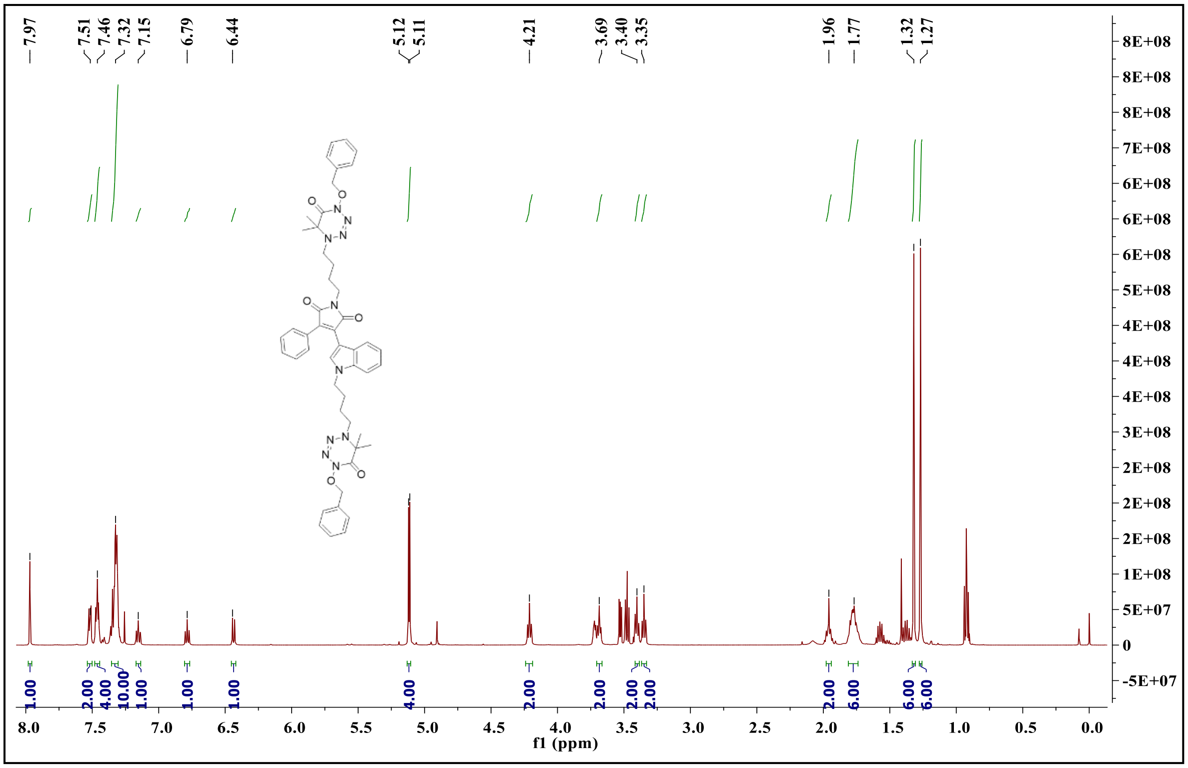

Figure S29. ^{1}H-NMR spectrum of compound **4e** ($CDCl_3$)

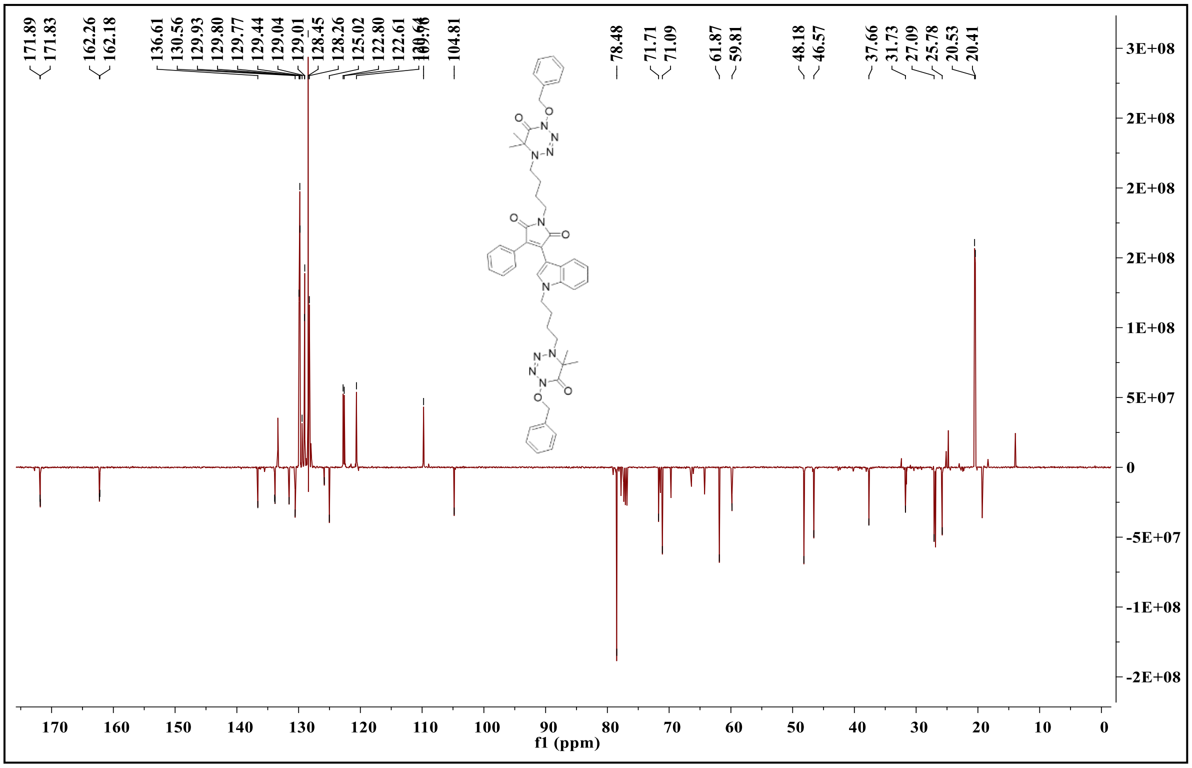

Figure S30. APT NMR spectrum of compound **4e** ($CDCl_3$)

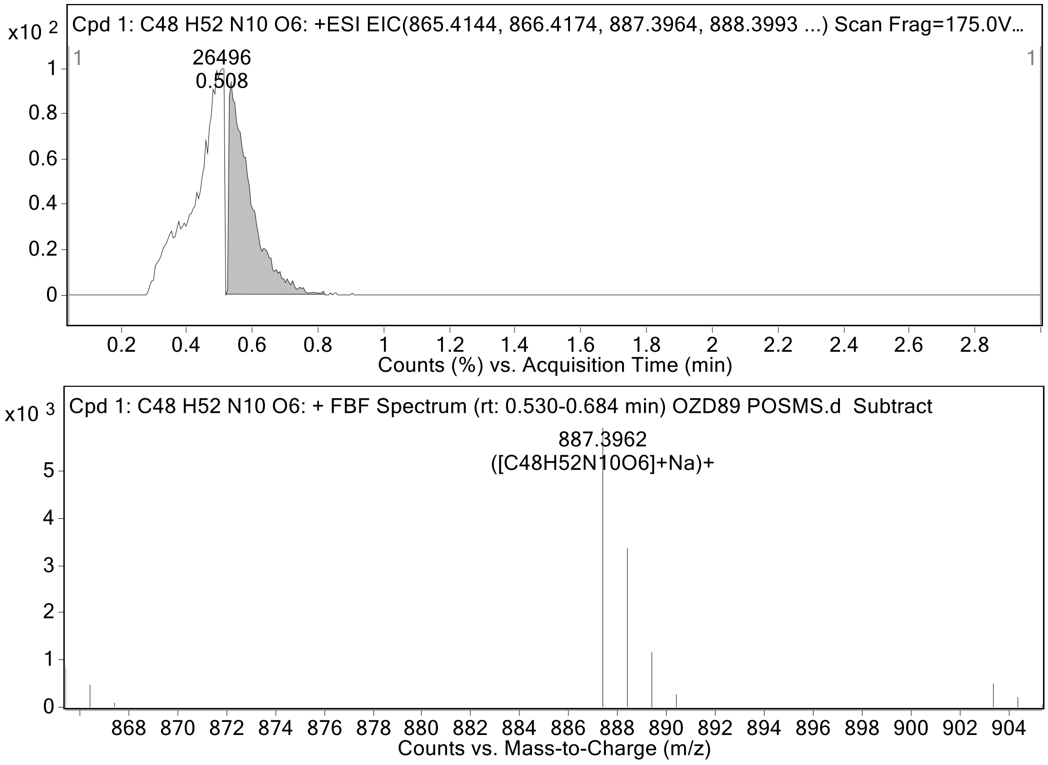

Figure S31. HR-MS (QTOF) spectrum of compound **4e**

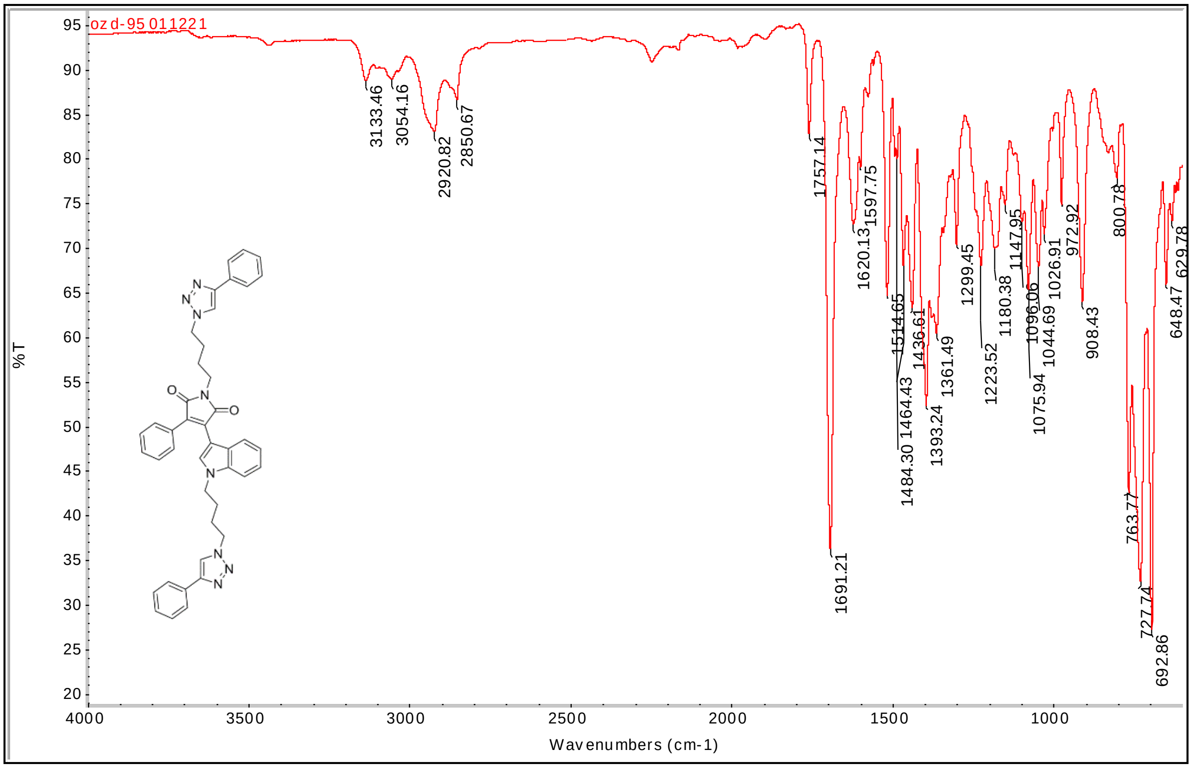

Figure S32. FTIR spectrum of compound **4f** (ATR)

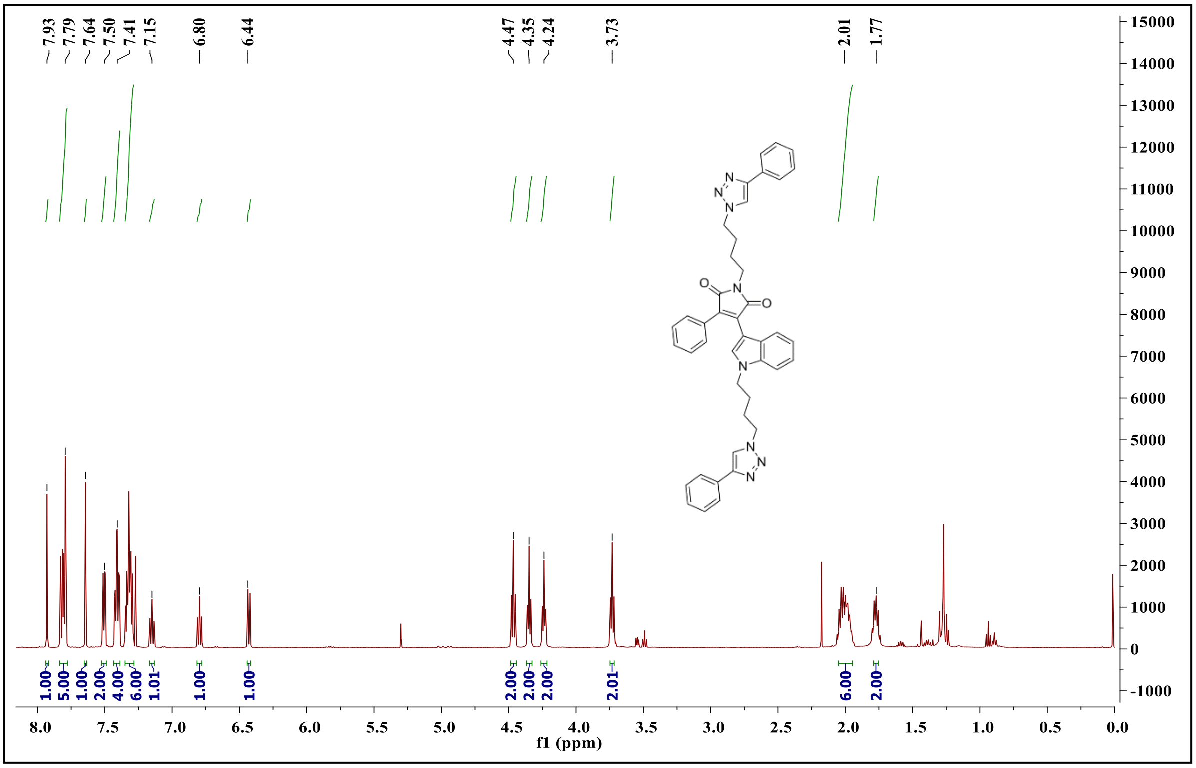

Figure S33. ^{1}H-NMR spectrum of compound **4f** ($CDCl_3$)

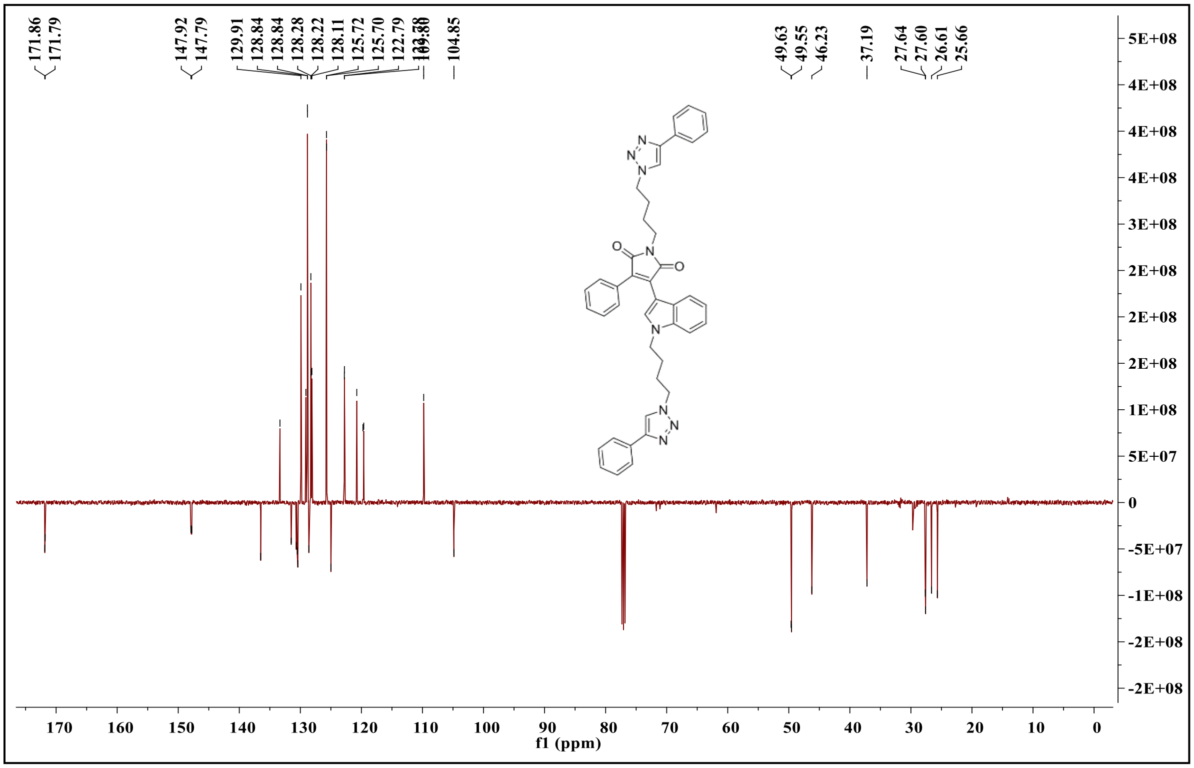

Figure S34. APT NMR spectrum of compound **4f** ($CDCl_3$)

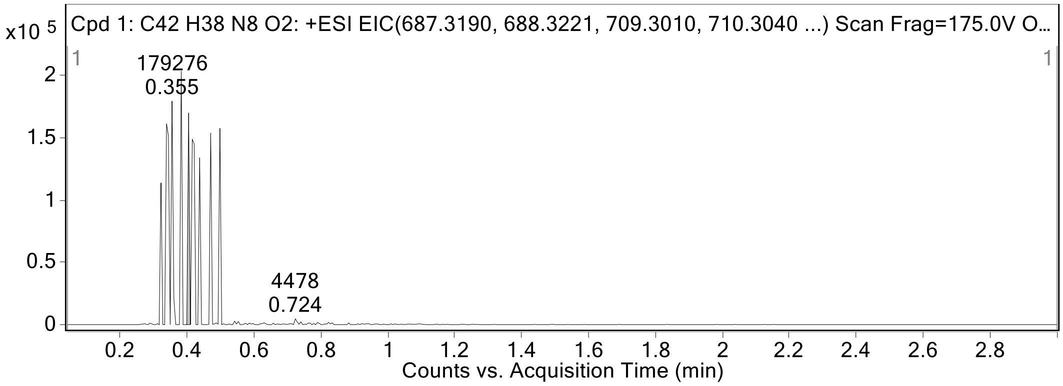

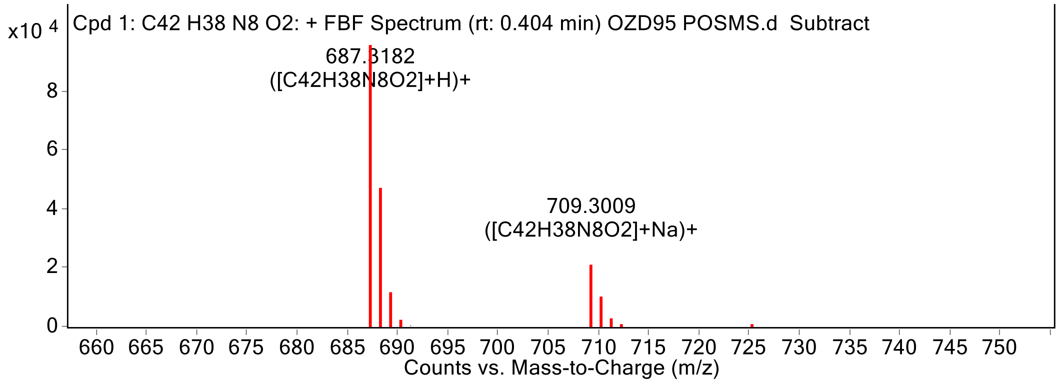

Figure S35. HR-MS (QTOF) spectrum of compound **4f**

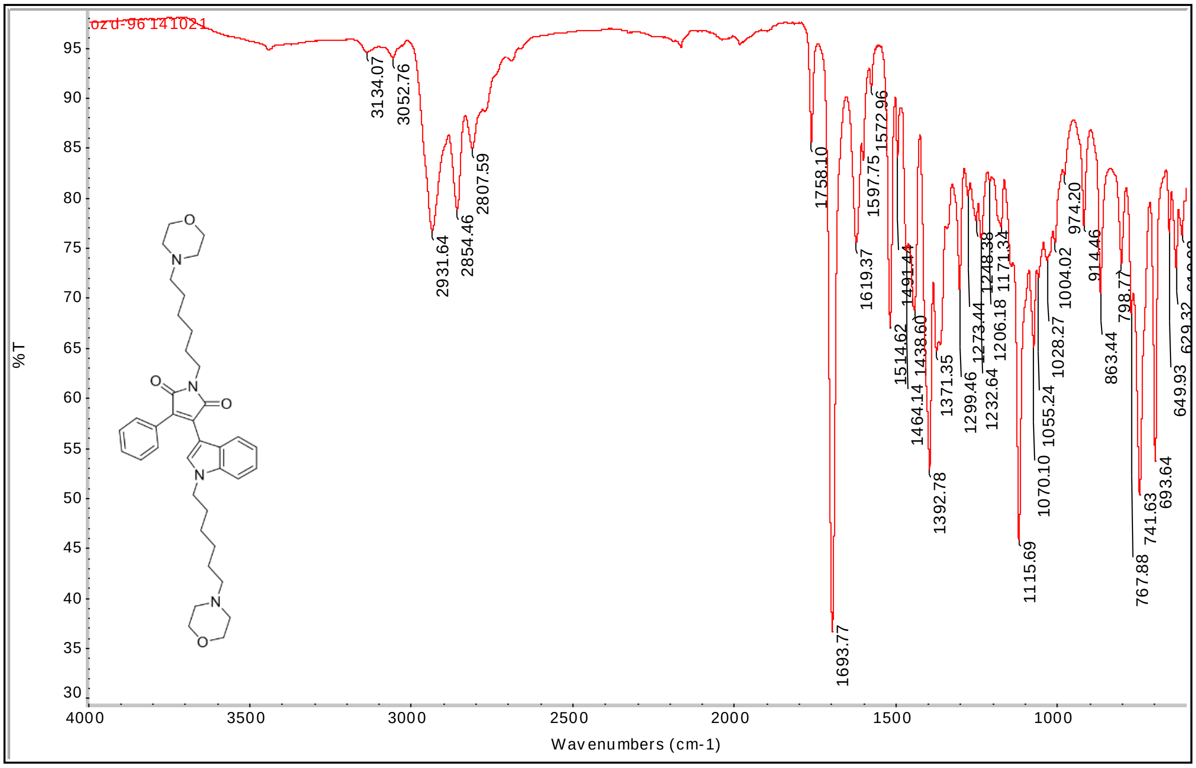

Figure S36. FTIR spectrum of compound **5a** (ATR)

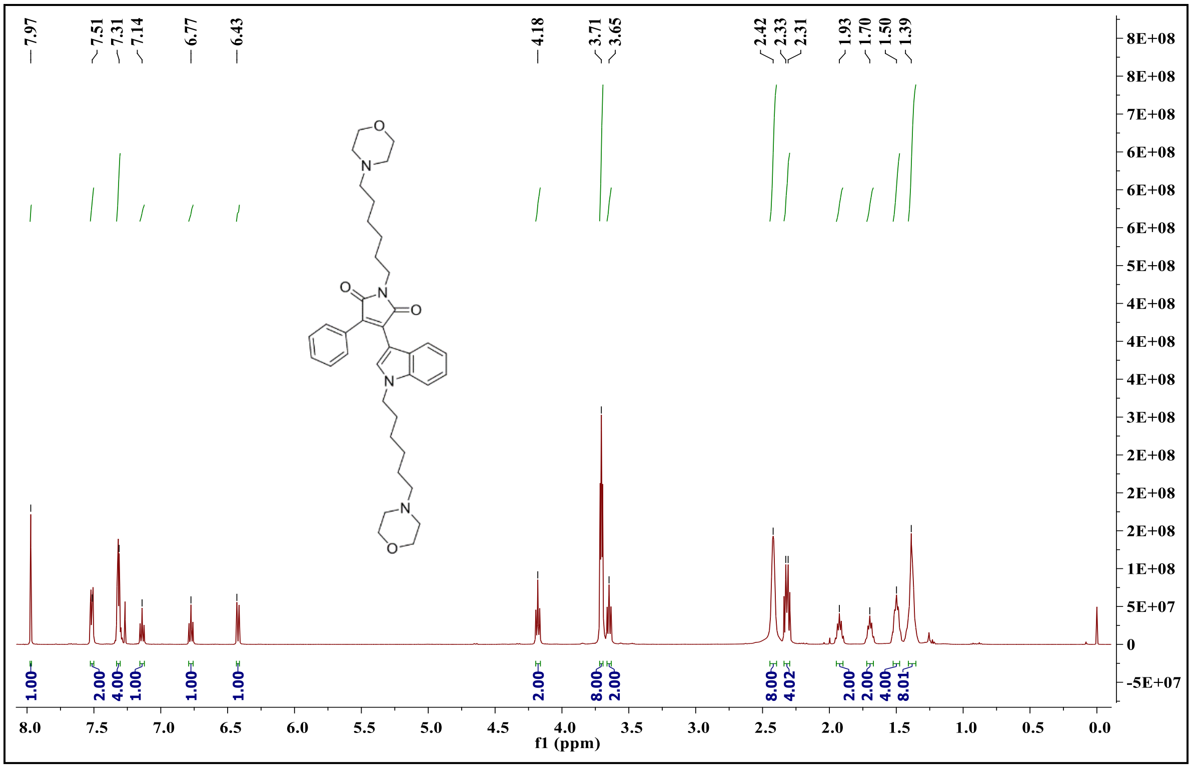

Figure S37. ^{1}H-NMR spectrum of compound **5a** ($CDCl_3$)

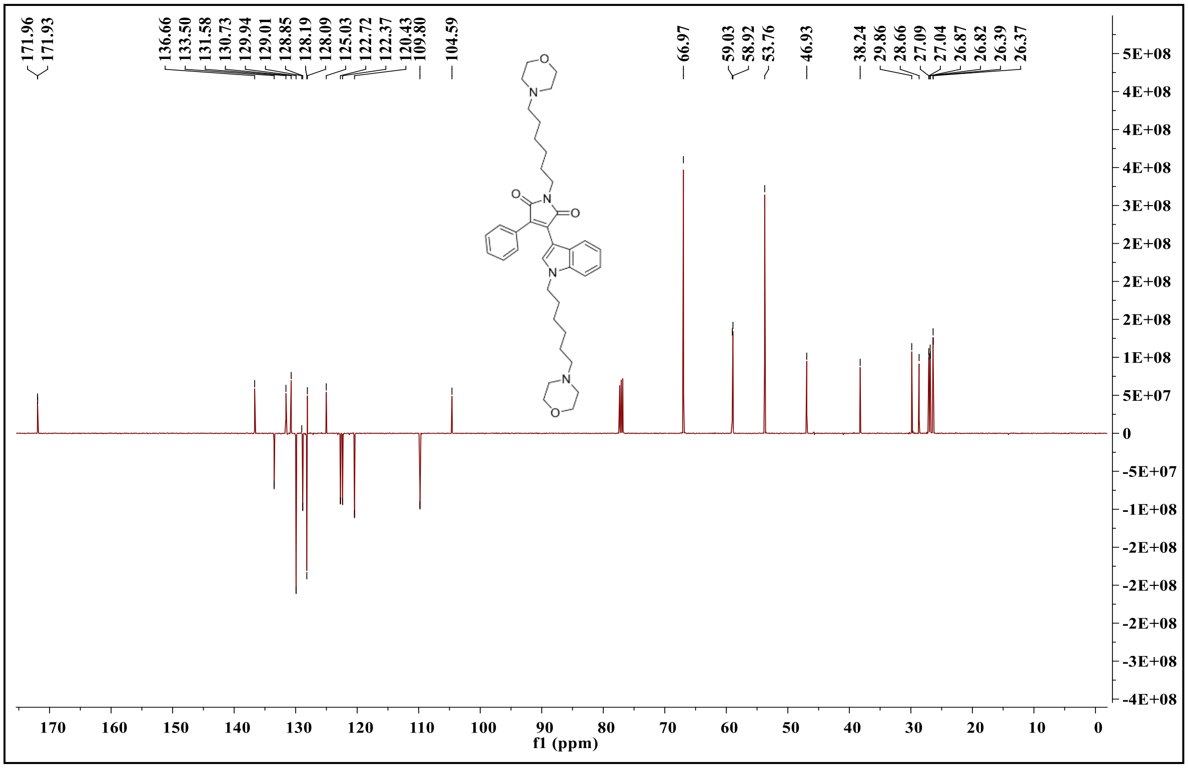

Figure S38. APT NMR spectrum of compound **5a** ($CDCl_3$)

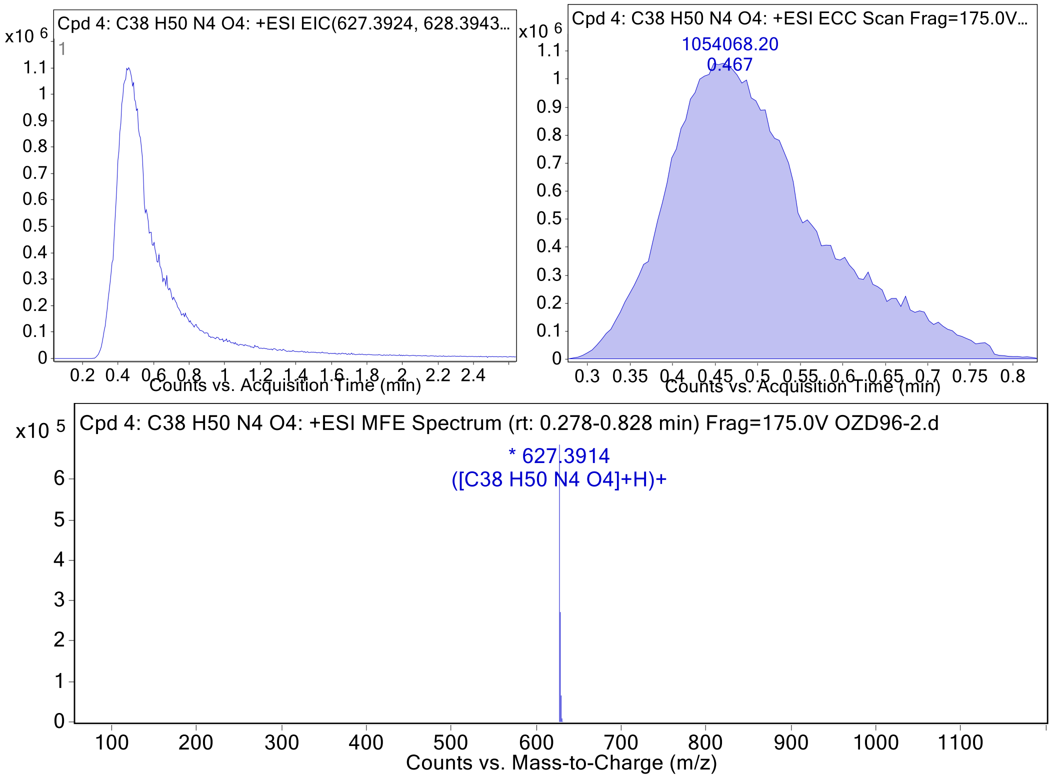

Figure S39. HR-MS (QTOF) spectrum of compound **5a**

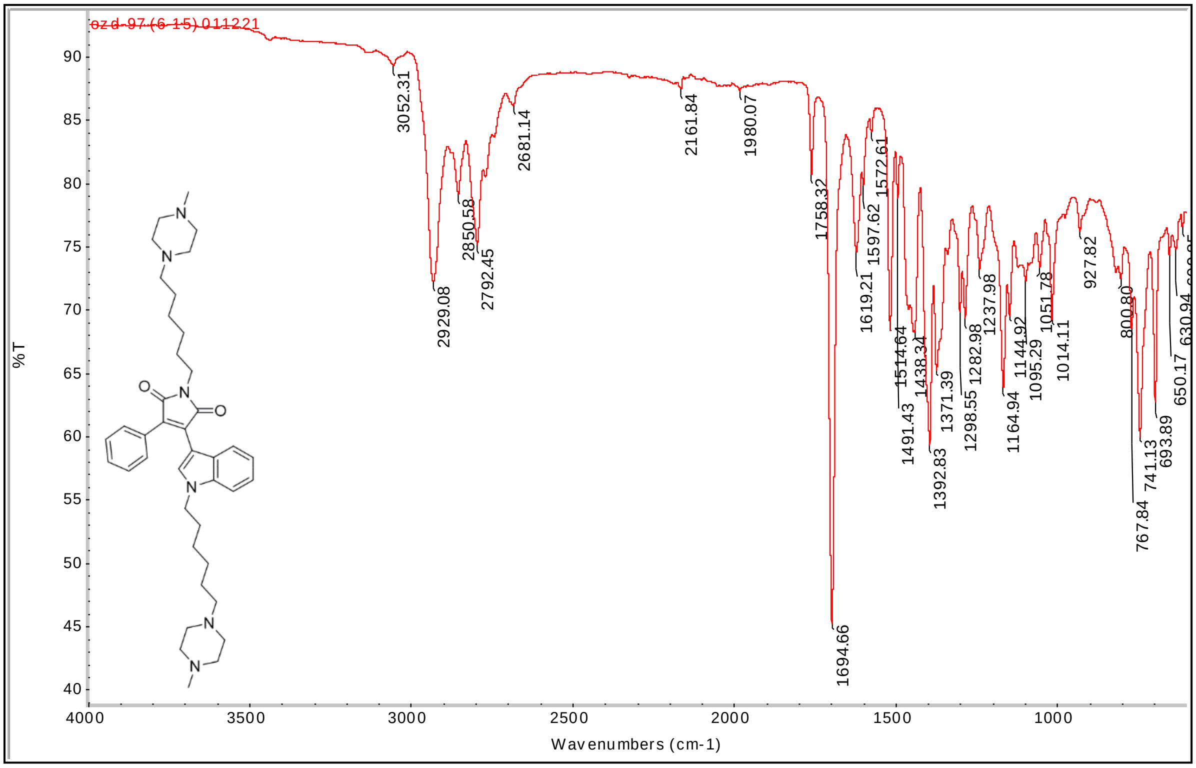

Figure S40. FTIR spectrum of compound **5b** (ATR)

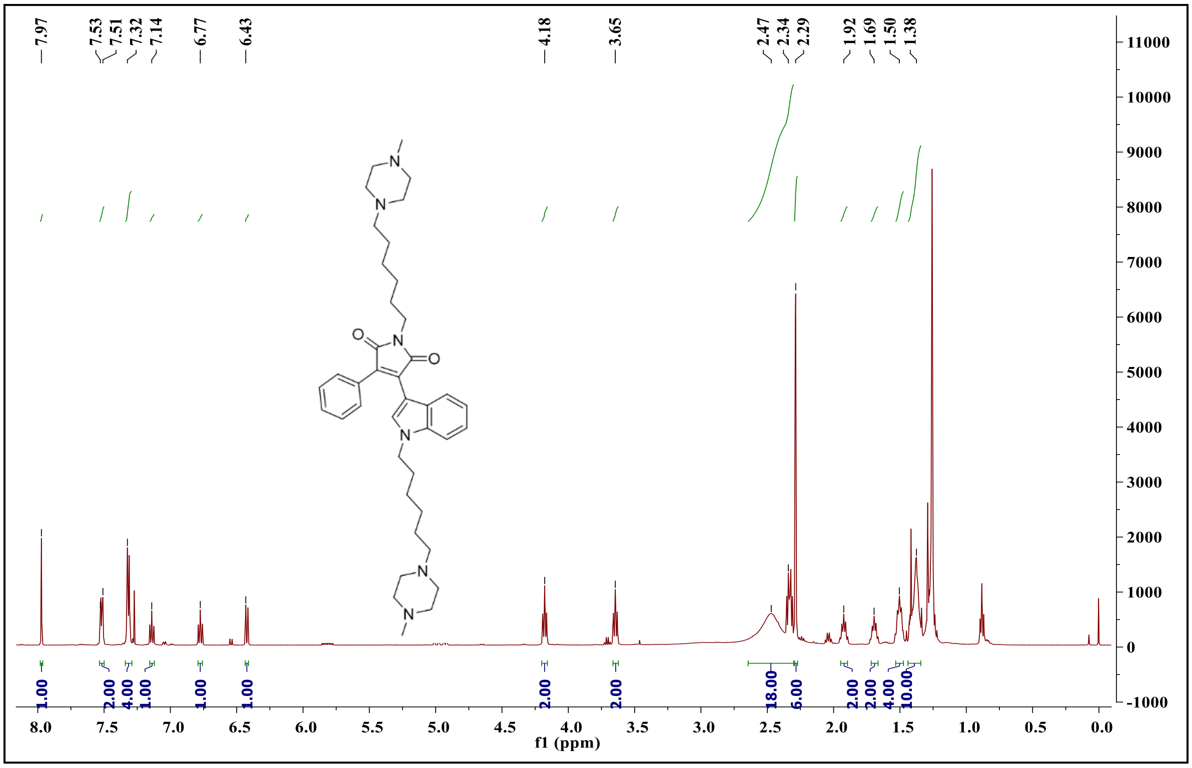

Figure S41. ^{1}H-NMR spectrum of compound **5b** ($CDCl_3$)

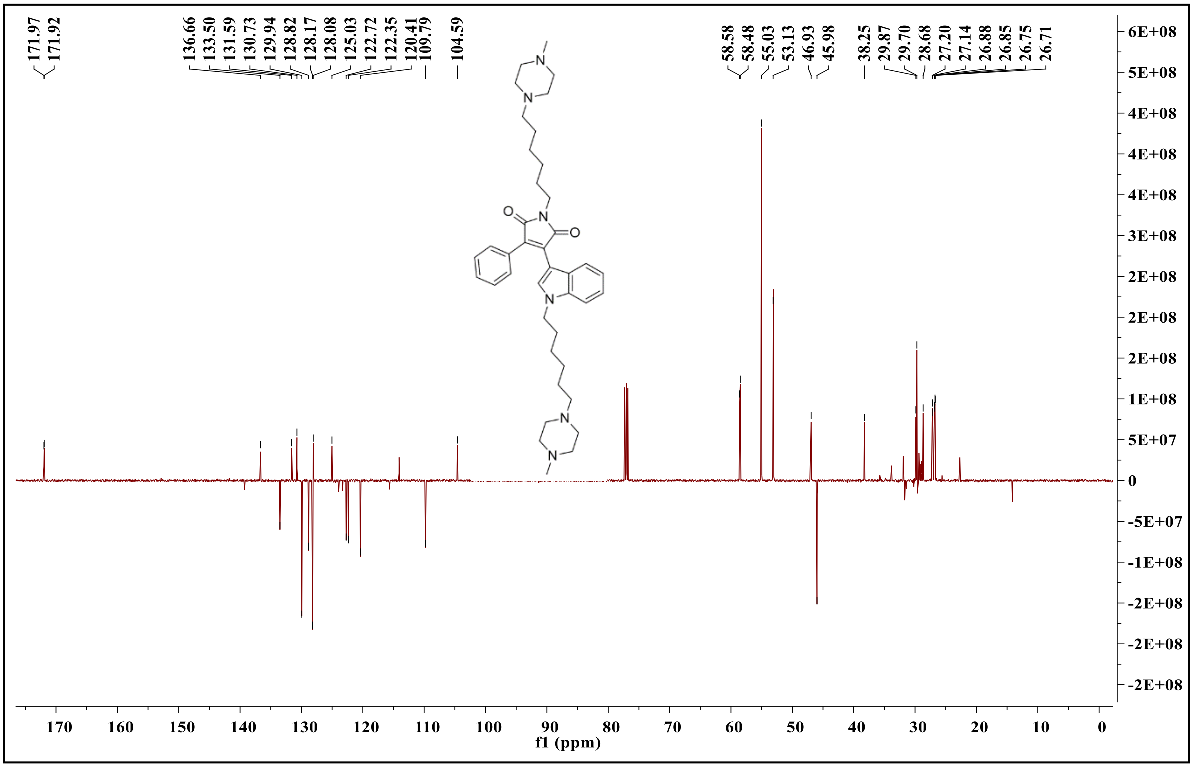

Figure S42. APT NMR spectrum of compound **5b** ($CDCl_3$)

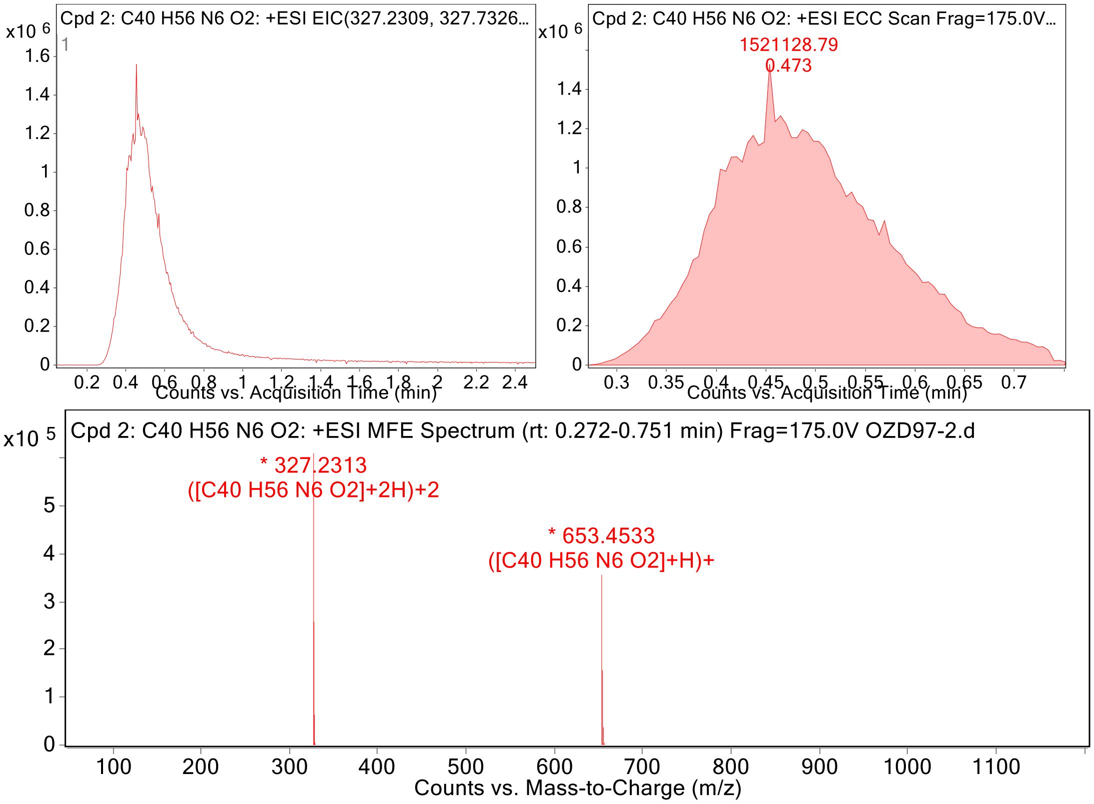

Figure S43. HR-MS (QTOF) spectrum of compound **5b**

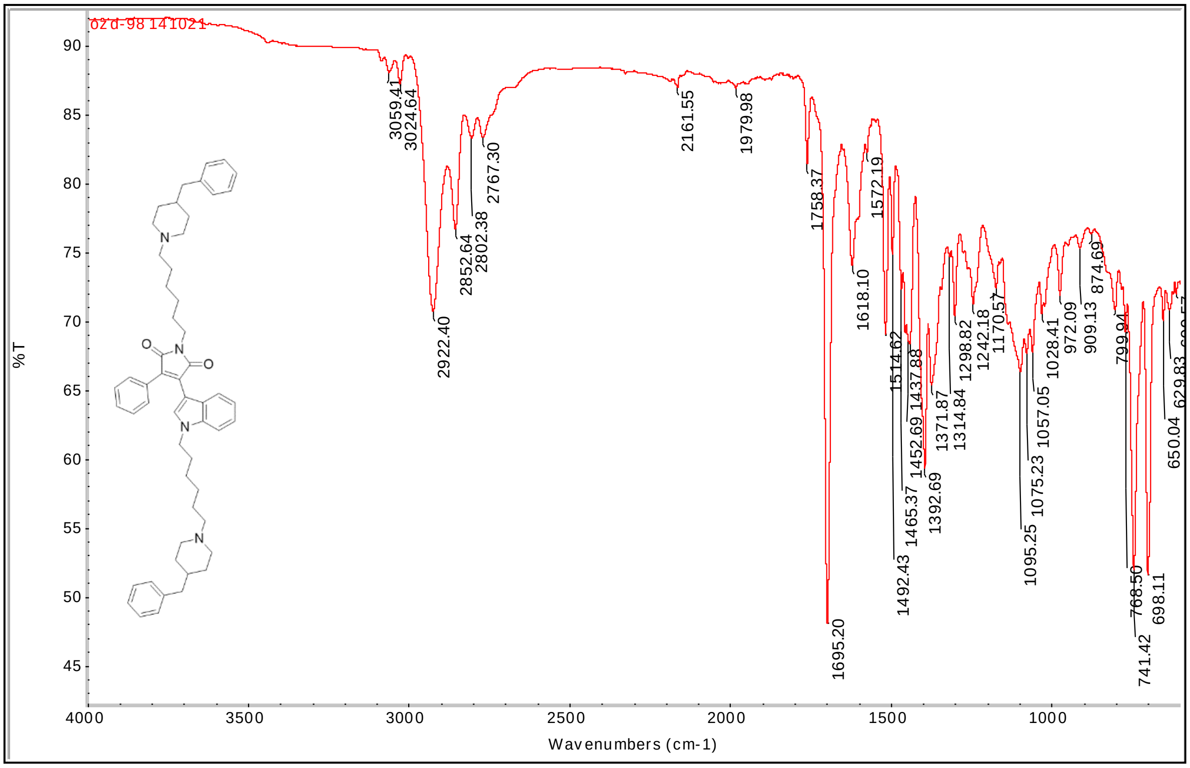

Figure S44. FTIR spectrum of compound **5c** (ATR)

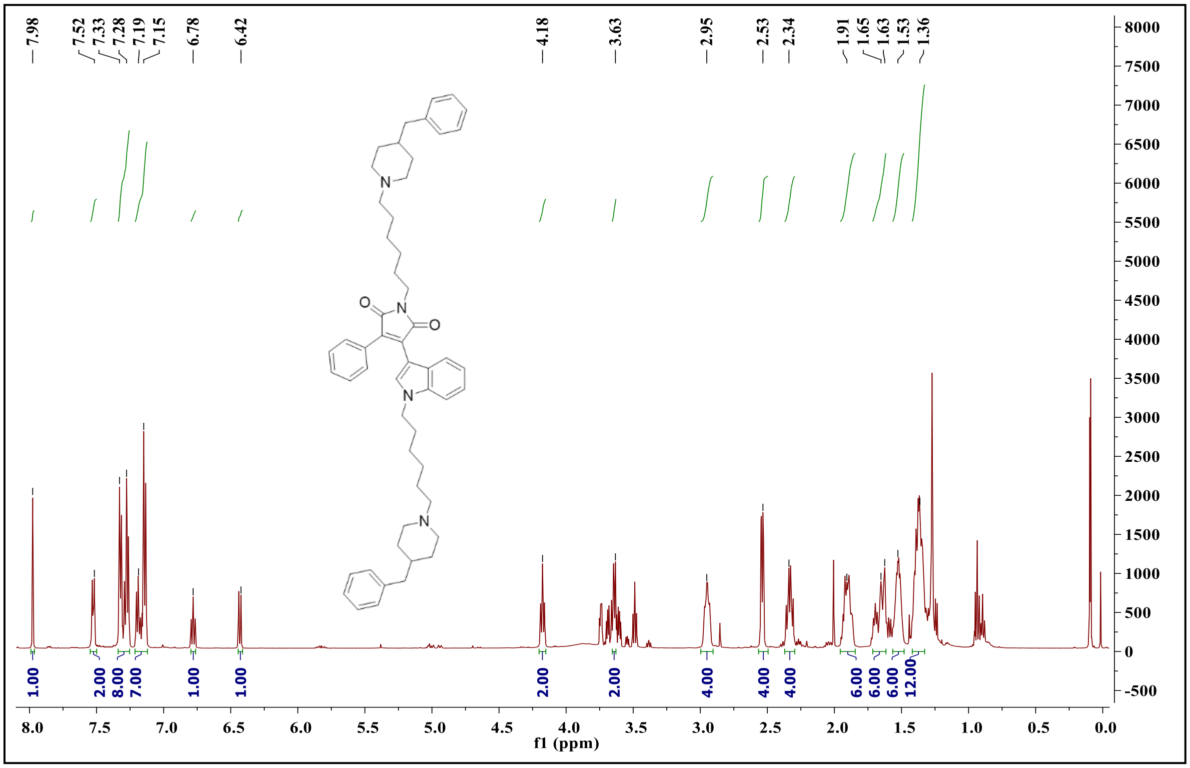

Figure S45. ^{1}H-NMR spectrum of compound **5c** ($CDCl_3$)

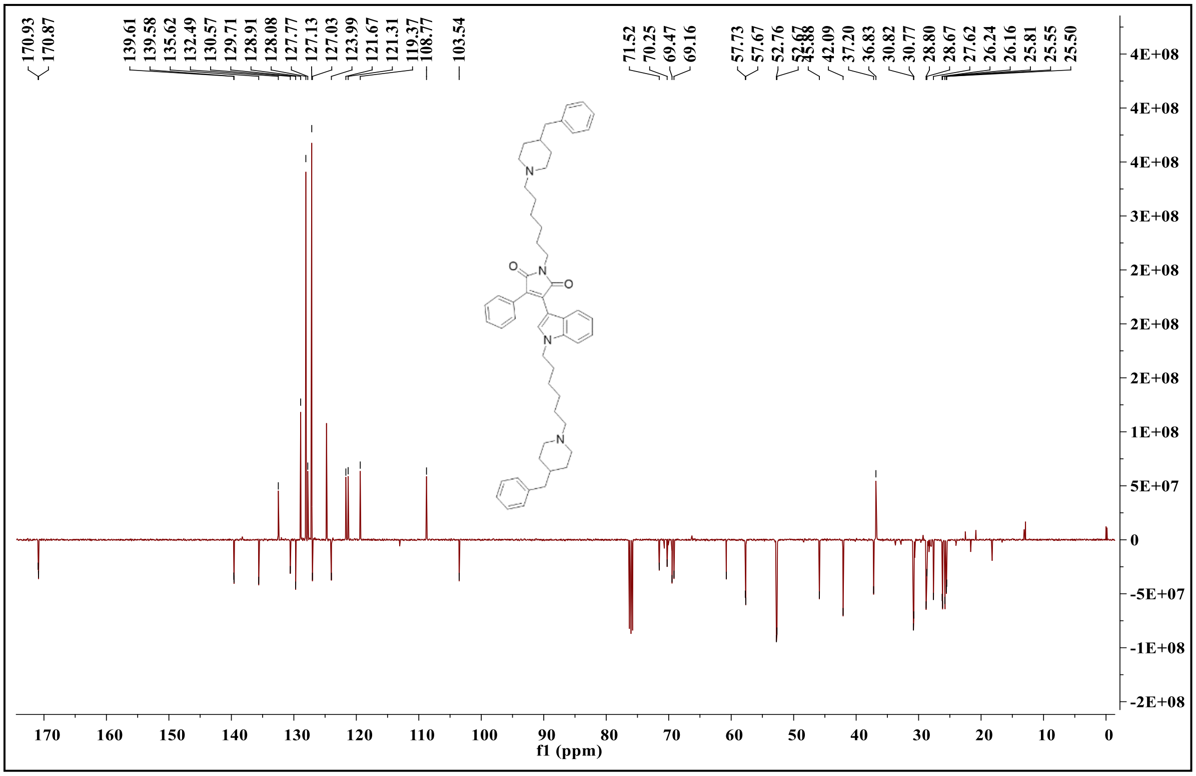

Figure S46. APT NMR spectrum of compound **5c** ($CDCl_3$)

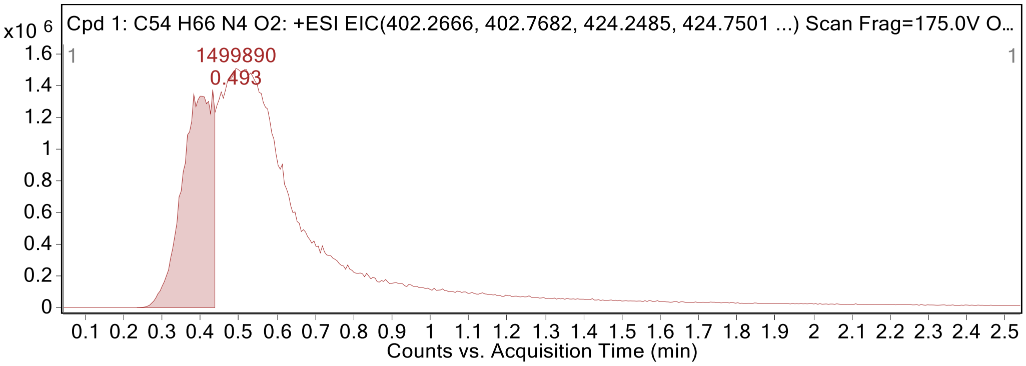

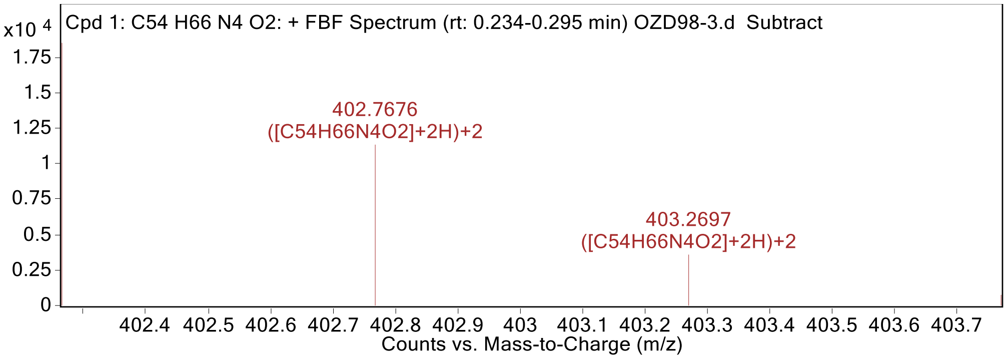

Figure S47. HR-MS (QTOF) spectrum of compound **5c**

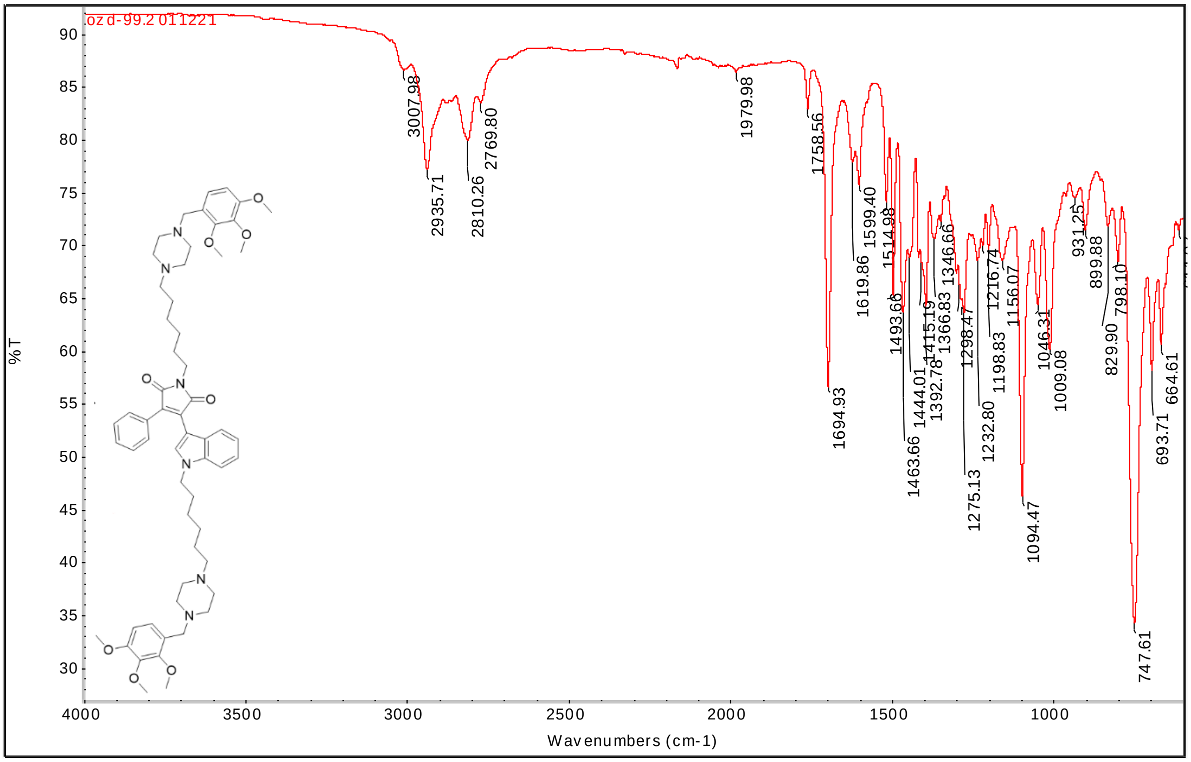

Figure S48. FTIR spectrum of compound **5d** (ATR)

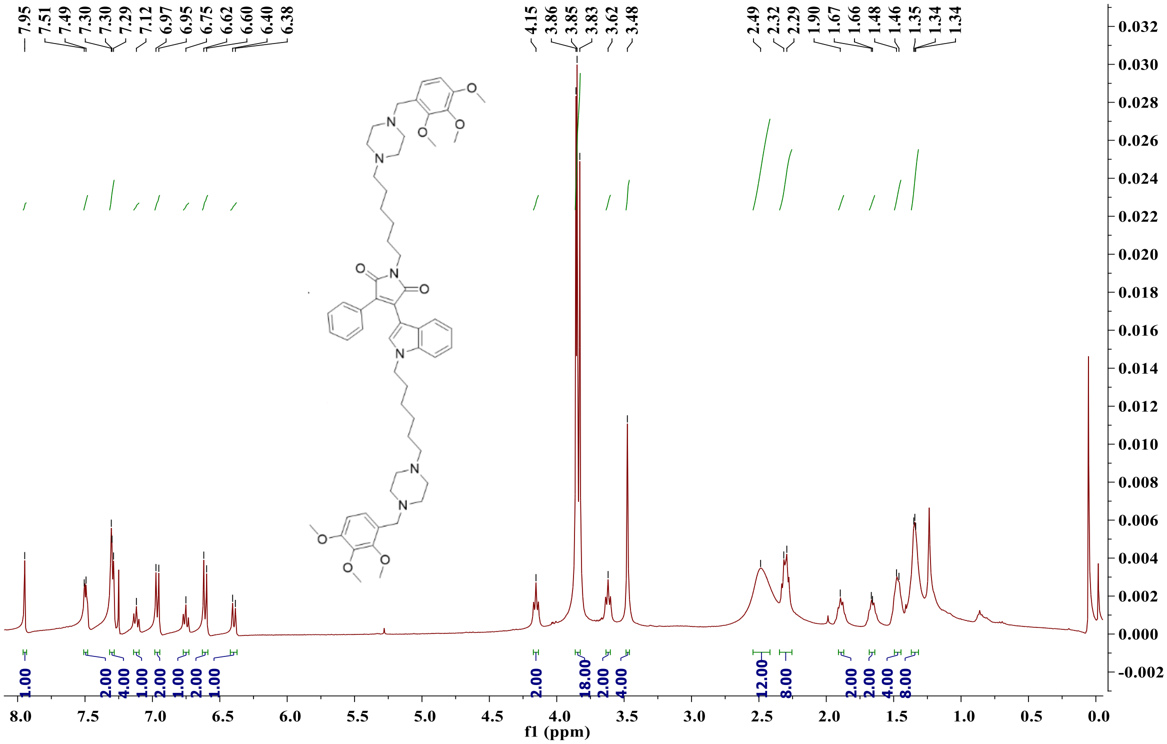

Figure S49. ^{1}H-NMR spectrum of compound **5d** ($CDCl_3$)

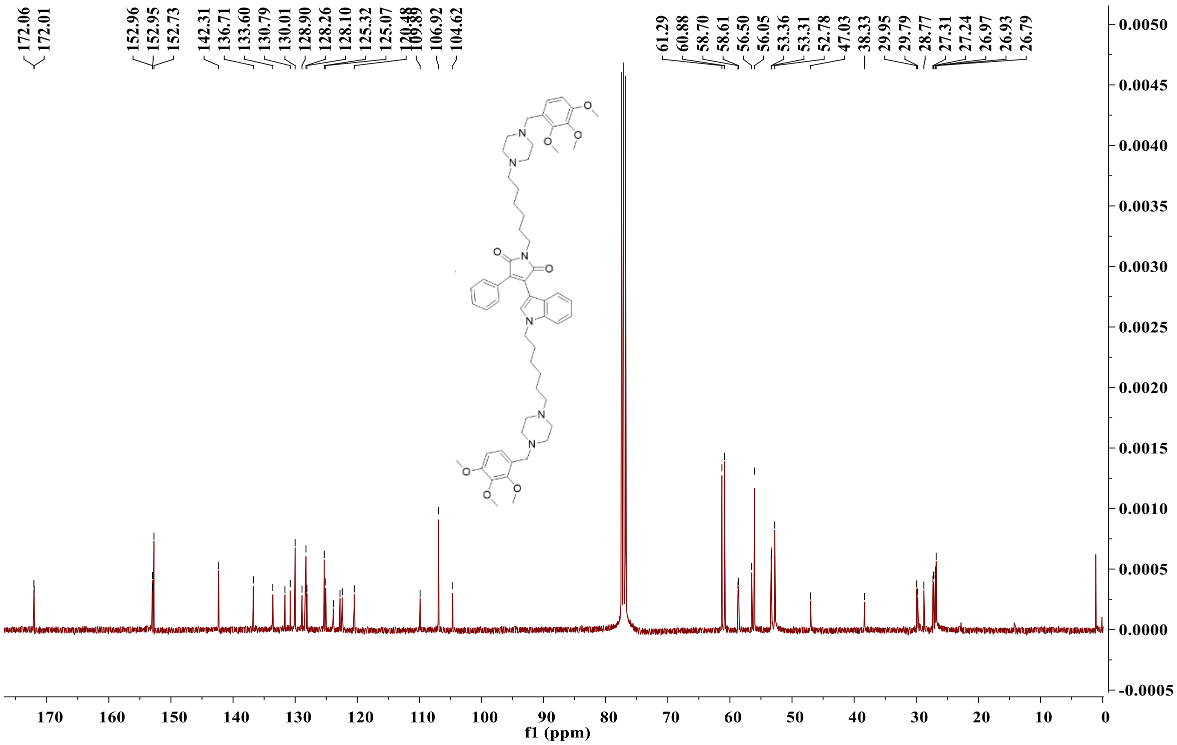

Figure S50. ^{13}C-NMR spectrum of compound **5d** ($CDCl_3$)

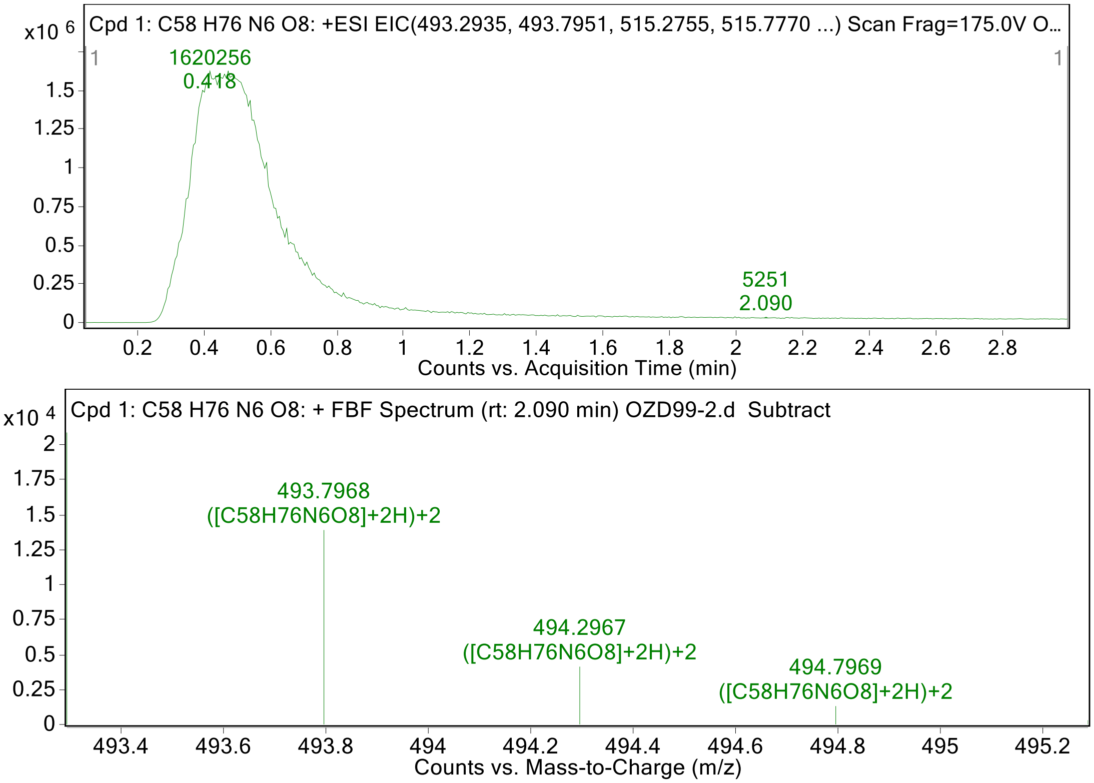

Figure S51. HR-MS (QTOF) spectrum of compound **5d**

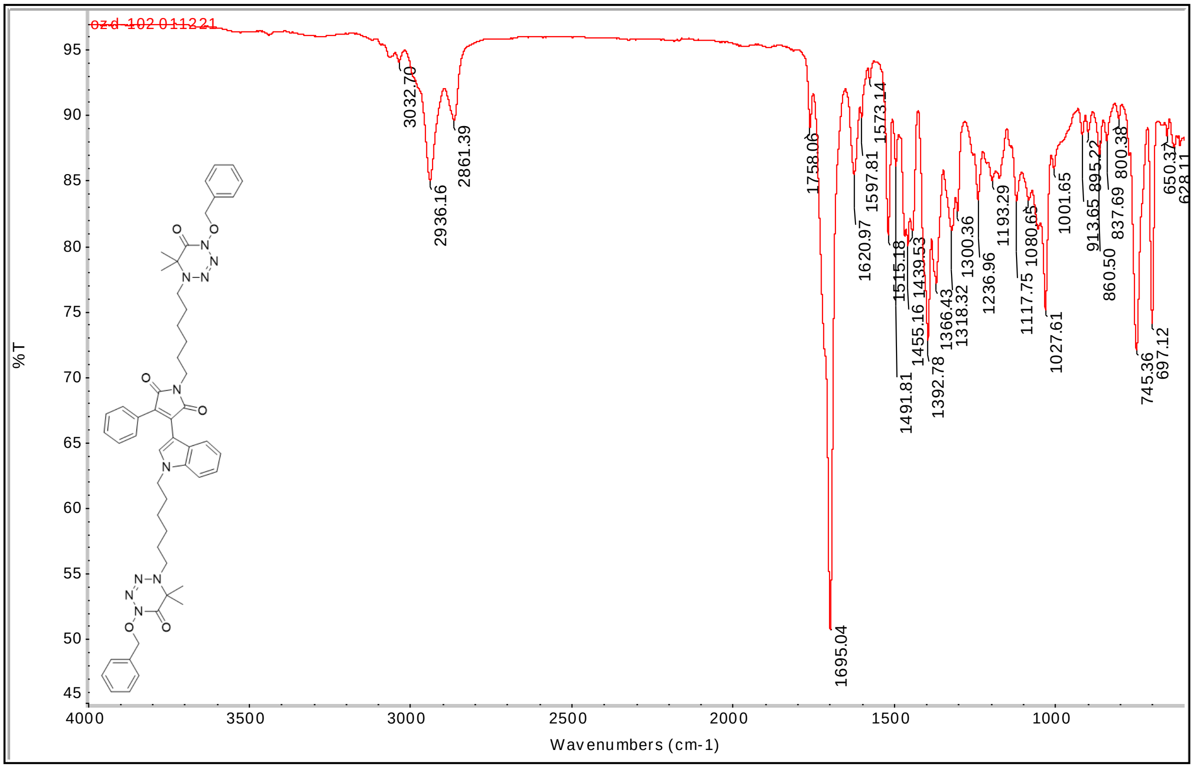

Figure S52. FTIR spectrum of compound **5e** (ATR)

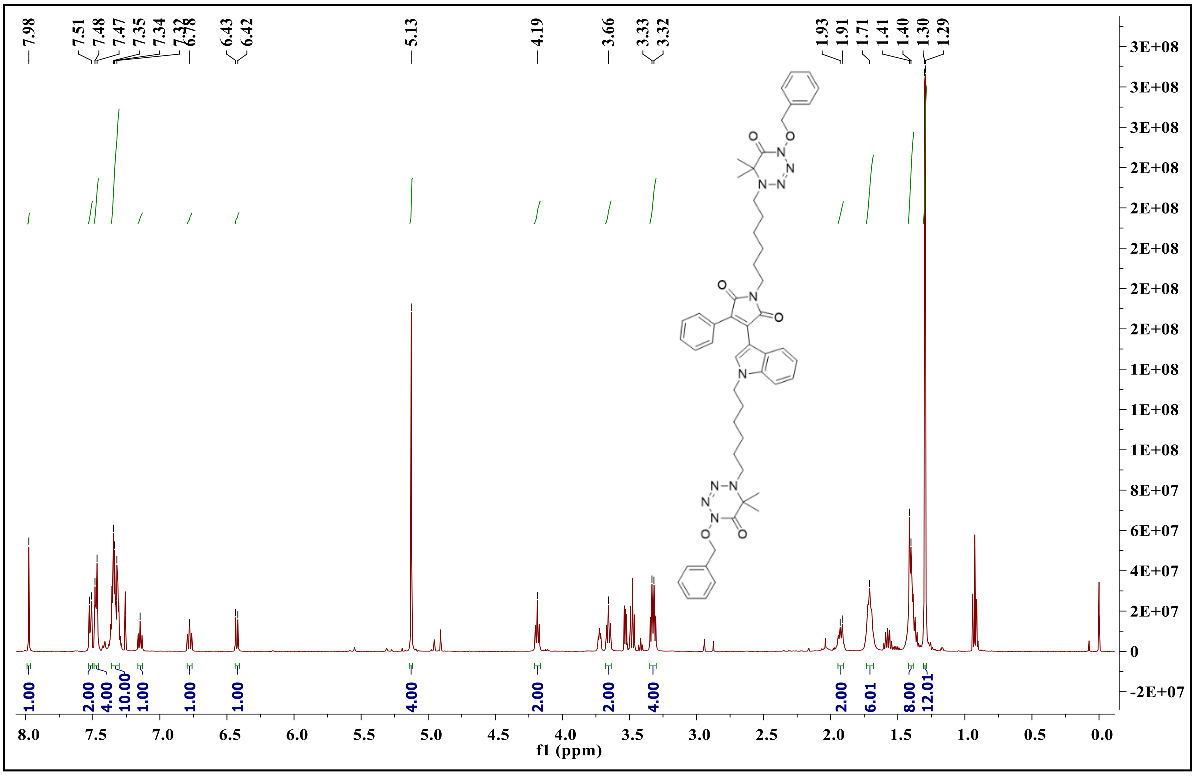

Figure S53. ^{1}H-NMR spectrum of compound **5e** ($CDCl_3$)

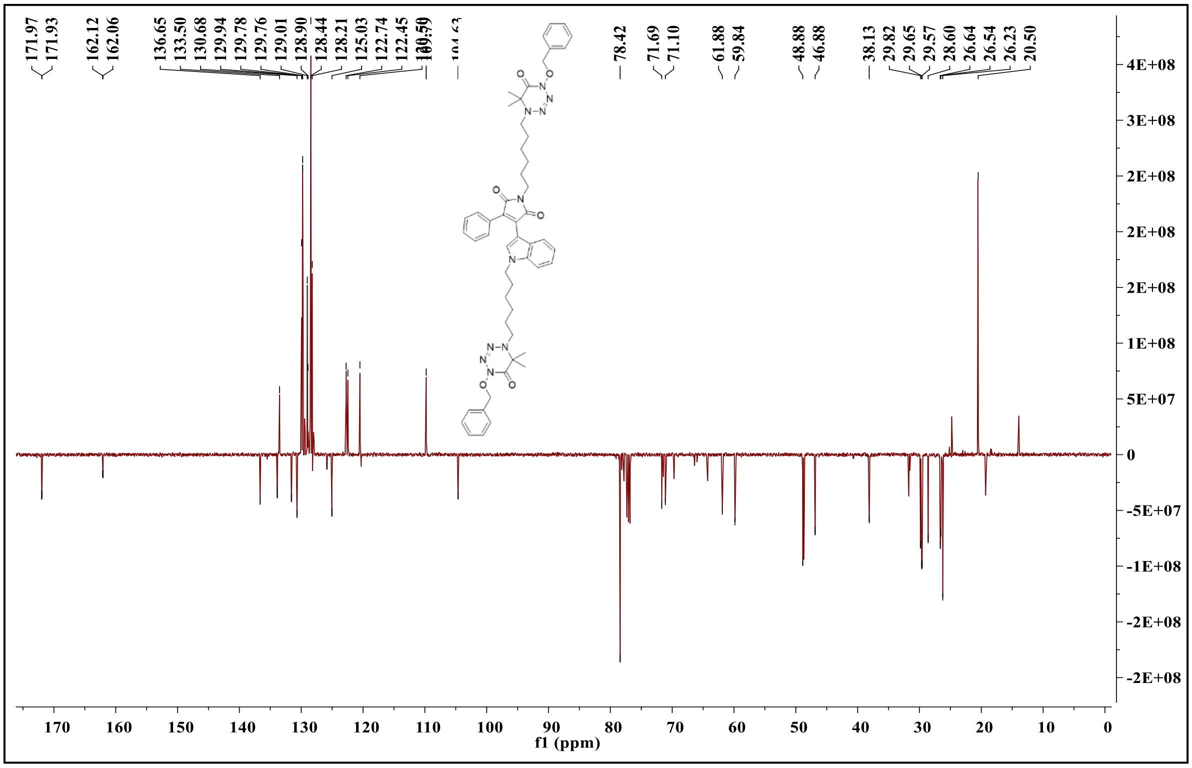

Figure S54. APT NMR spectrum of compound **5e** ($CDCl_3$)

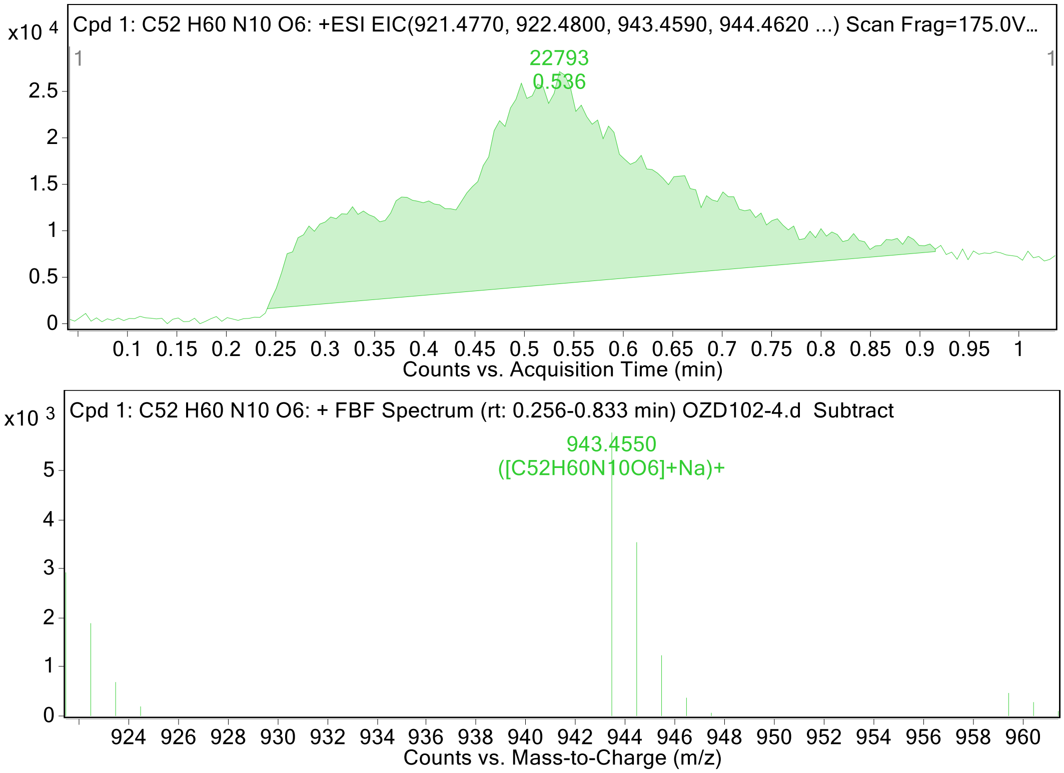

Figure S55. HR-MS (QTOF) spectrum of compound **5e**

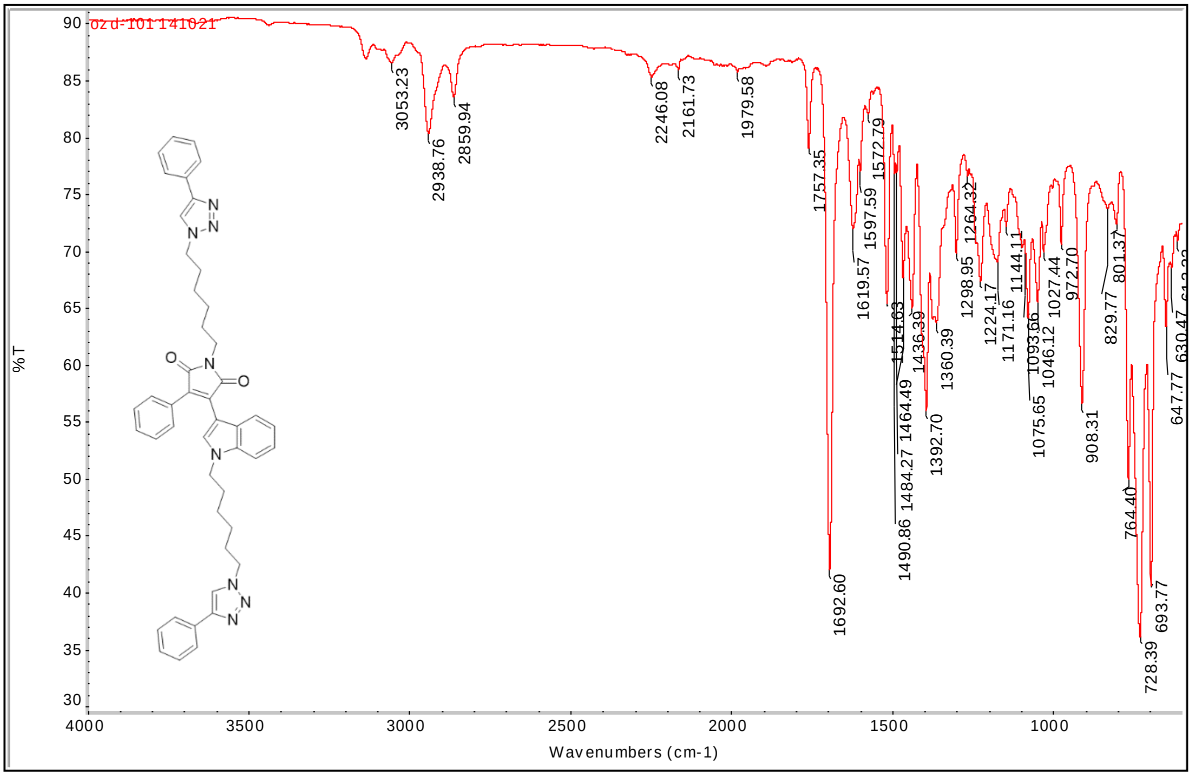

Figure S56. FTIR spectrum of compound **5f** (ATR)

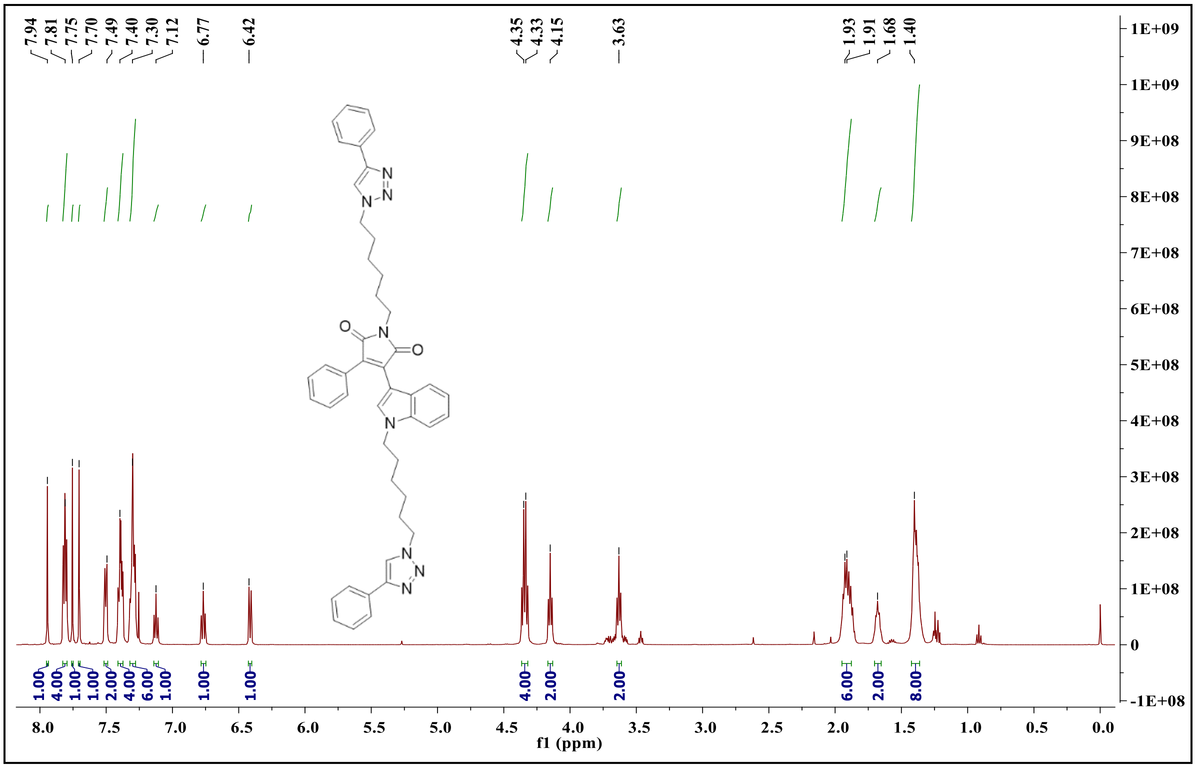

Figure S57. ^{1}H-NMR spectrum of compound **5f** ($CDCl_3$)

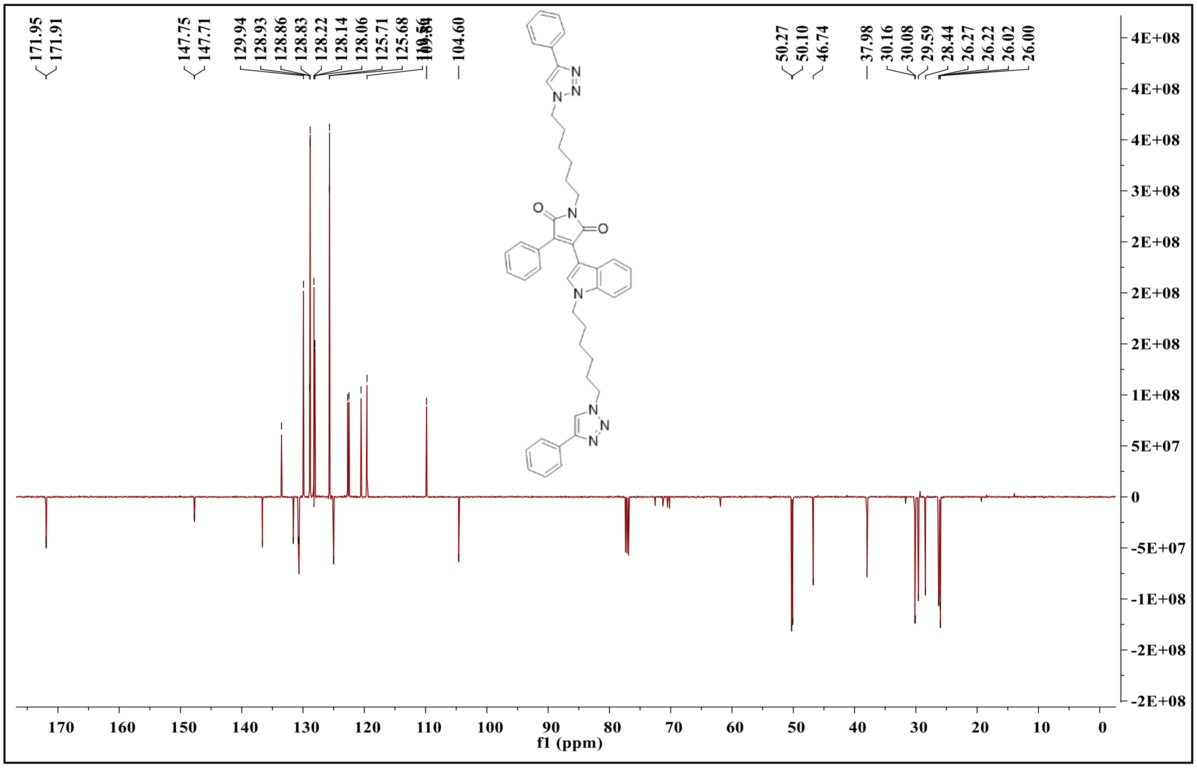

Figure S58. APT NMR spectrum of compound **5f** ($CDCl_3$)

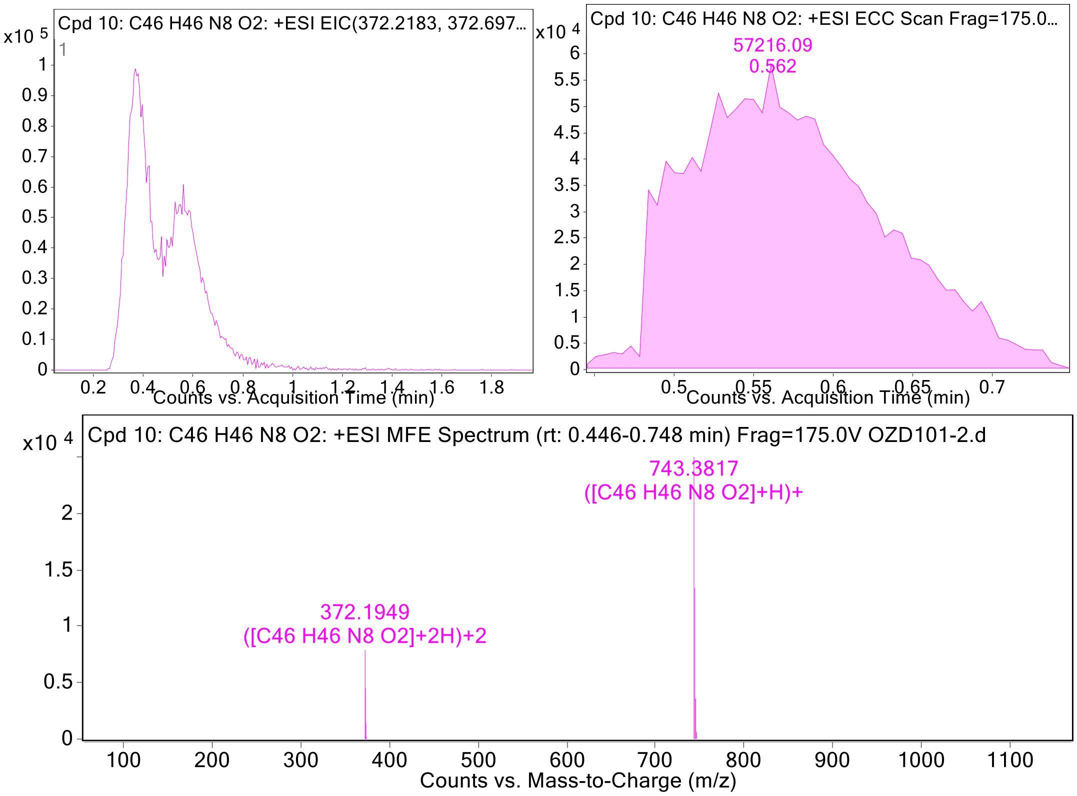

Figure S59. HR-MS (QTOF) spectrum of compound **5f**

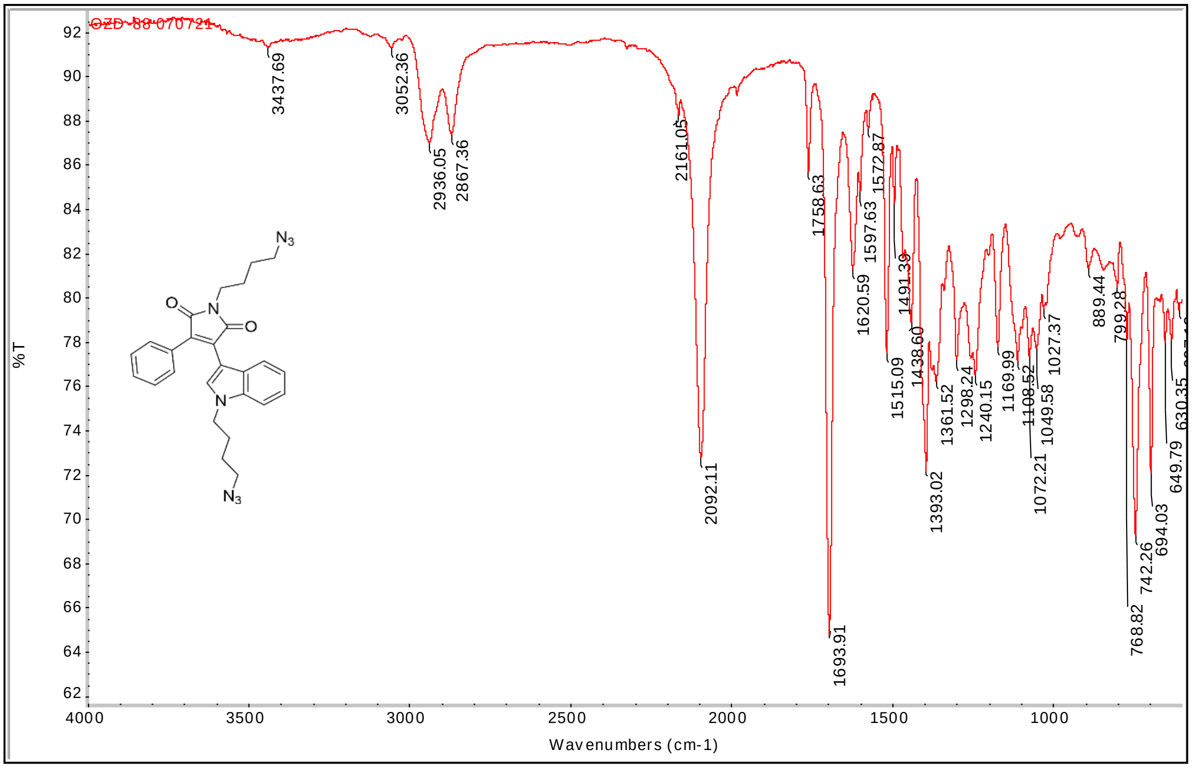

Figure S60. FTIR spectrum of compound **6a** (ATR)

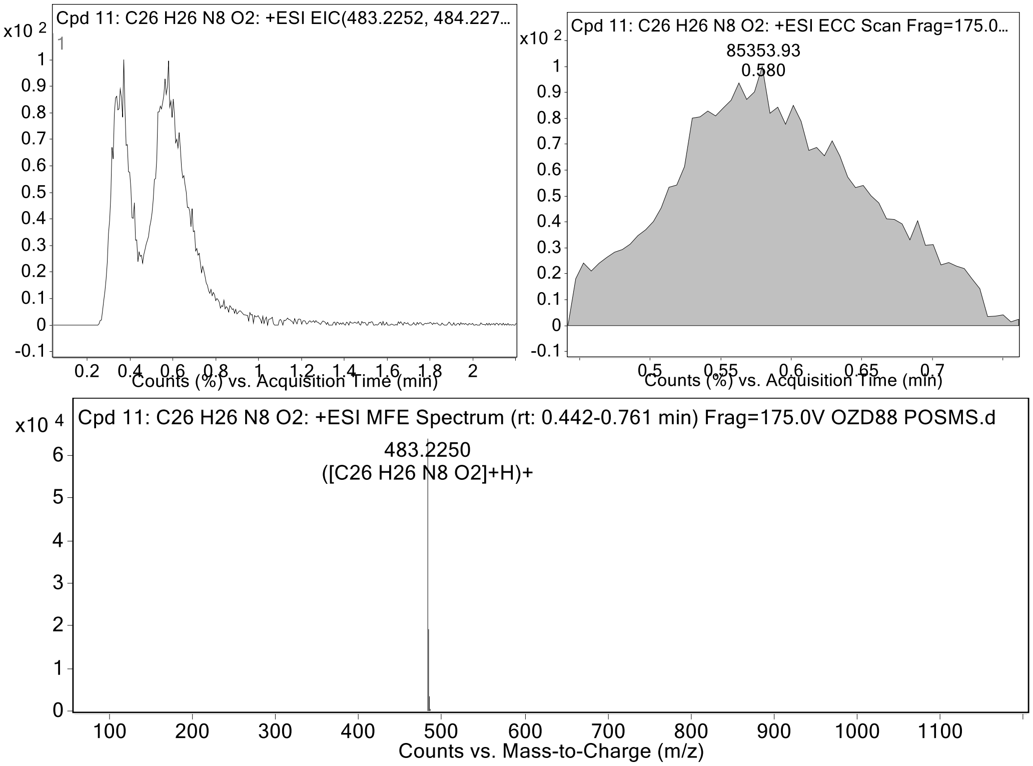

Figure S61. HR-MS (QTOF) spectrum of compound **6a**

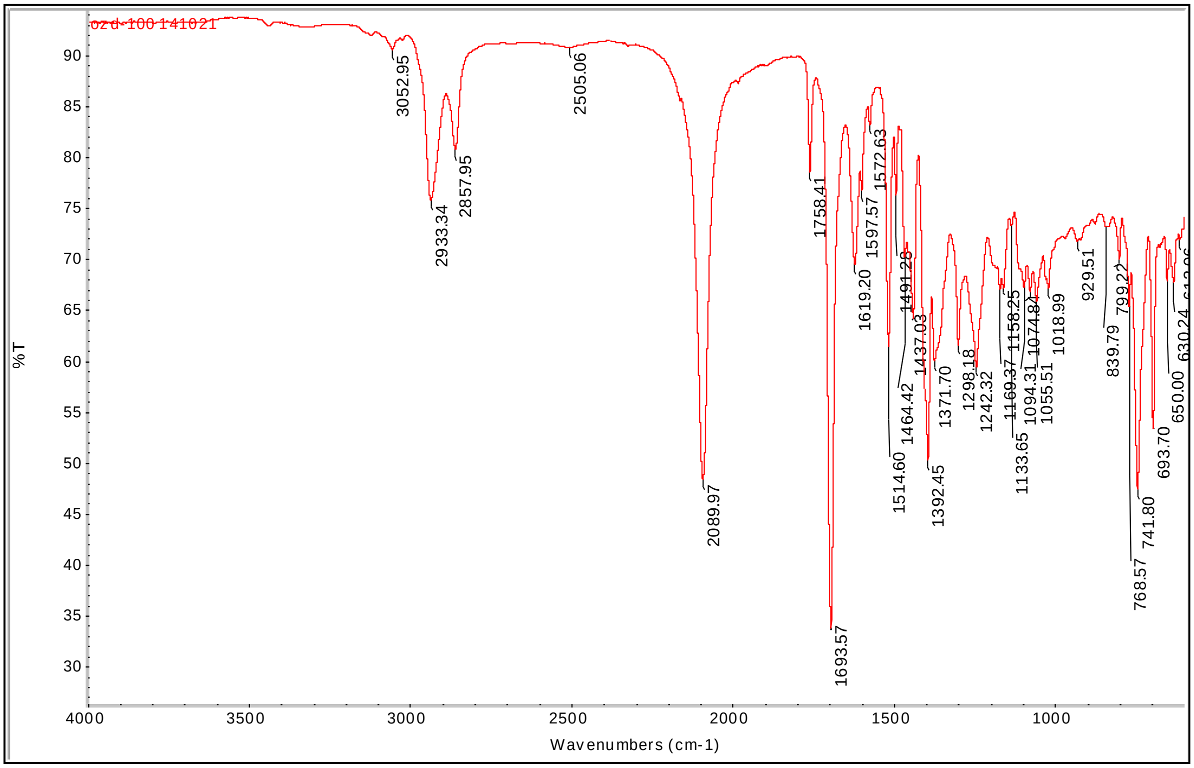

Figure S62. FTIR spectrum of compound **6b** (ATR)

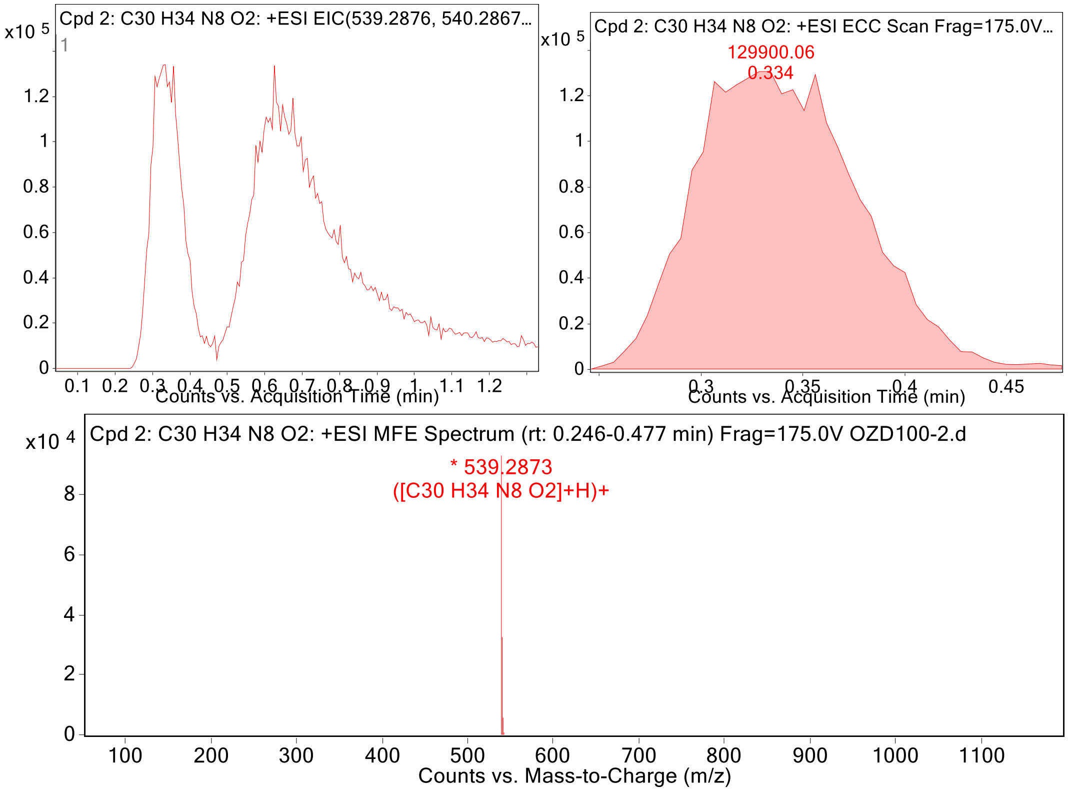

Figure S63. HR-MS (QTOF) spectrum of compound **6b**

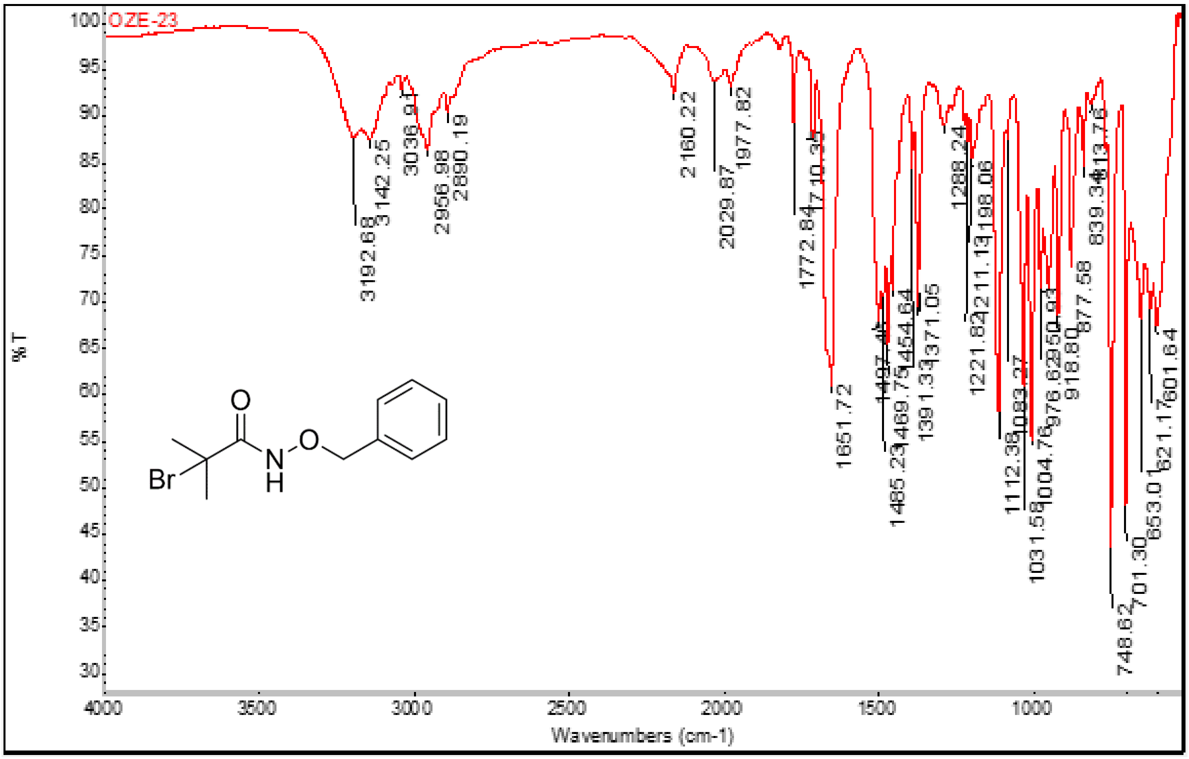

Figure S64. FTIR spectrum of compound **7** (ATR)

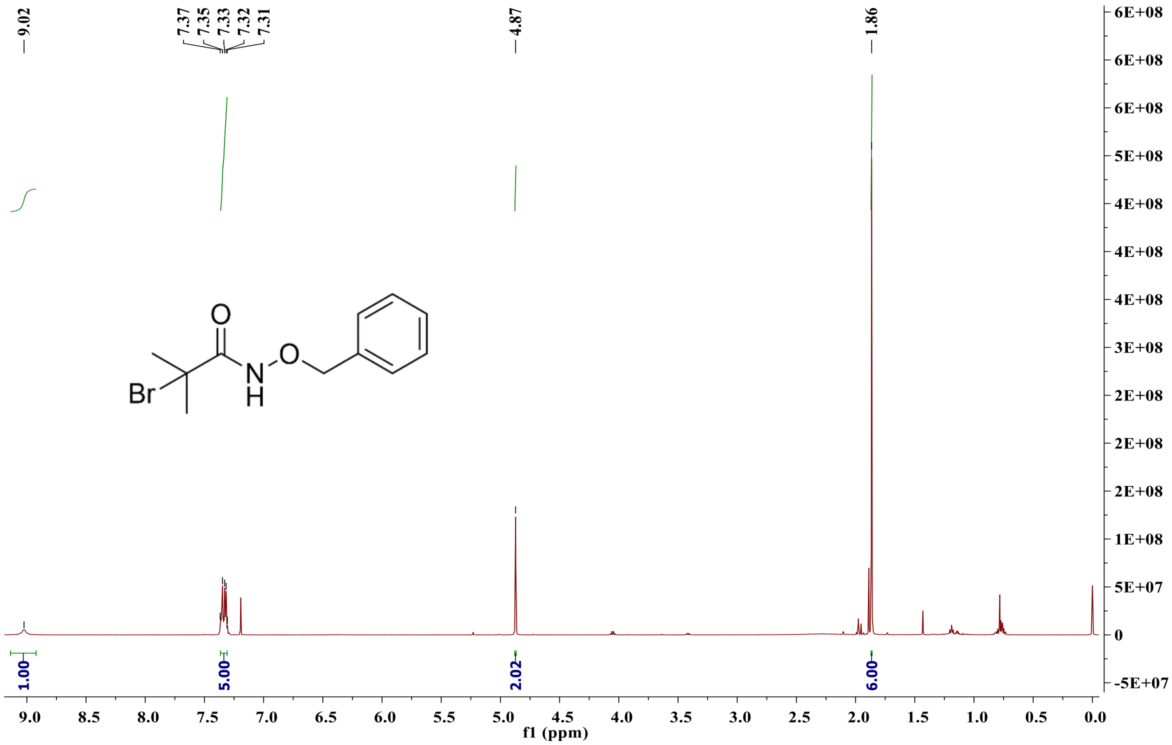

Figure S65. ^{1}H-NMR spectrum of compound **7** ($CDCl_3$)

2. MTT Assay Studies

2.1 Cell lines and culture condition

MCF-7 and MDA-MB-231 cell lines were cultured in RPMI medium, supplemented with 10% heat inactivated fetal bovine serum and 1% penicillin and streptomycin; and all the cells were incubated at 5% CO_2 and 37 °C. All cell lines were kindly provided by Dr. M. Sayitoglu and Dr. O. Hatirnaz-Ng from Acibadem University.

2.2 Cell proliferation assay

MTT assay [5] was used to evaluate the growth inhibition effect of the compounds on breast cancer cell lines (MDA-MB-231 and MCF-7).

Cells were plated at a density of 1x10E5 cells/ml on the 96-wells plates 24 hours before the drug incubation.

Stock solutions (33.3 – 10 – 5 - 1 mM and 300 µM) of all the compounds were prepared in DMSO were diluted with the medium before adding to the cells. The percentage of DMSO of the final solution was kept under 1.2% due to its toxicity. An increasing concentration (0.3-0.5-1-3-5-10-15-20-30-50-70-100-150-200 µM) of each compound were applied to cells with 6 replicates and incubated for 24 hours.

After incubation, 100 µl of supernatant was removed from each well and 10 µl of MTT solution (5 mg/mL) was added to each well and incubated at 37 °C for 4 hours. The 50 µl of supernatant was dis-carded from each well and 100 µl of DMSO was added to dissolve formazan crystals. Plates were shaken for 45 minutes at room temperature. Then the optical density of each well was measured (using Thermo VarioSkan Flush Multimode Reader Quantum) at 570 nm wavelength. The IC_{50} values were calculated by using Graphpad

Prism. To determine the viability rate, it was calculated by the following formula from the spectrophotometric results.

%VR: 100-[(OD(control)-OD(sample)) / *100]/OD(control)

OD: Optical density

2.3 Statistical Analyses for IC50

The IC_{50} values were calculated by using GraphPad Prism. All the data presented as the mean of 6 replicates. Results were analyzed and illustrated with Graph Pad Prism (version 5; GraphPad Software, San Diego, CA, USA). Statistical analysis was performed using dose-response inhibition, log(inhibitor) vs response-variable slope, least squares (ordinary) fit.

Cheminformatics

Absorption, Distribution, Metabolism, /Excretion and Toxicity (ADMET) Prediction

The investigation of the absorption, distribution, metabolism, excretion, and toxicity, the so-called ADMET properties of a compound, is a crucial step in the drug development process. The molecular properties and structural features affecting the drug-likeness of the synthesized molecules were evaluated on the Swissadme website (Table S1). To better understand the effects of newly synthesized compounds, GSK-3β drug candidate molecules (LY2090314, Laduviglusib, CHIR-99021-HCl, Alsterpaullone, CHIR-98014, SB216763 and AR-A014418) and two drugs (Doxorubicin and Paclitaxel) currently used in breast cancer treatment were calculated from the Swiss-Adme website and added to the tables. Except for the molecular weight criteria **4a**, **4b**, **5a** and **5b** were found to be in accordance with the Lipinski rule of 5, estimated solubility properties and the bioavalibality score of them were better than the other compounds. Also, bioavailability radar diagram of the compounds was presented in Figure S66.

Predicting gastrointestinal absorption and brain access of the drug is crucial in the discovery of new drugs. Therefore, The Brain or Intestinal Estimated permeation method (Boiled-Egg), which works by calculating the lipophilicity and polarity of small molecules, has been proposed in Figure S67 and Table S2.[6] According to the calculation on the SwissAdme website, it was estimated that compounds **4a**, **4b**, **5a** and **5b** could cross the blood brain barrier, while compounds **4d** and **4f** would exhibit high solubility, **4e**, **5d** and **5e** would exhibit low solubility in the gastrointestinal tract. Compounds **4c**, **5c** and **5f** were out of range.

Cytochrome P450 (CYP) enzyme family plays an important role in the breakdown of drugs to be eliminated from the body. Inhibition of these enzymes delays the elimination of drugs from the body. Five enzymes of this family (CYP1A2, CYP2C19, CYP2C9, CYP2D6, CYP3A4) play an important role in rendering therapeutic molecules harmless.[7] In Table S3, according to the data obtained from the Swiss-Adme website, the potential of the synthesized compounds to inhibit these enzymes has been shown. According to the calculation, the ability of the synthesized compounds to inhibit the CYP450 enzyme gave similar results to the GSK-3β drug candidates for breast cancer.

P-glycoprotein (P-gp) is a plasma membrane protein that actively removes drugs from the cell and influences drug metabolism and excretion from the body. Thus, it reduces the intracellular concentration of cancer drugs and enables the development of resistance against them.[8] All the new synthesized compounds were found to inhibit P-gp activity, which is a desirable characteristic for an anticancer molecule.

Table S1. Molecular descriptors, drug-likeness, bioavalibity scores of the synthesized derivatives

**Comp.**	**Lipinski**	**Molecular weigth (g/mole)**	**MlogP**	**HBA**	**HBD**	**TPSA (Å)**	**RB**	**natom**	**Violations for Lipinski**	**Water Solubility ESOL (mg/ml)**	**ESOL Class**	**Bioavalibility score**
4a	Yes	570.72	2.43	6	0	67.25	12	42	MW>500	0.00488	Moderately soluble	0.55
4b	Yes	596.81	2.79	6	0	55.27	12	44	MW>500	2.11E-03	Moderately soluble	0.55
4c	No	747.02	6.29	4	0	48.79	16	56	MW>500, MlogP>4.15	1.02E-07	Poorly soluble	0.17
4d	No	929.15	2.37	12	0	110.65	22	68	MW>500, NorO>10	1.99E-06	Poorly soluble	0.17
4e	No	864.99	4.6	10	0	157.31	18	64	MW>500, MlogP>4.15, NorO>10	7.87E-08	Insoluble	0.17
4f	No	690.84	4.98	6	0	98.23	14	52	MW>500, MlogP>4.15	8.94E-07	Poorly soluble	0.17
5a	Yes	626.83	3.14	6	0	67.25	16	46	MW>500	5.85E-04	Poorly soluble	0.55
5b	Yes	652.91	3.48	6	0	55.27	16	48	MW>500	2.51E-04	Poorly soluble	0.55
5c	No	803.13	6.9	4	0	48.79	20	60	MW>500, MlogP>4.15	1.20E-08	Insoluble	0.17
5d	No	985.26	2.97	12	0	110.65	26	72	MW>500, NorO>10	2.27E-07	Poorly soluble	0.17
5e	No	921.10	5.22	10	0	157.31	22	68	MW>500, MlogP>4.15, NorO>10	9.05E-09	Insoluble	0.17
5f	No	742.91	5.45	6	0	103.73	18	56	MW>500, MlogP>4.15	1.77E-06	Poorly soluble	0.17
LY2090314	Yes	512.53	2.56	5	1	91.95	4	38	MW>500	1.10E-02	Moderately soluble	0.55
Laduviglusib HCl (CHIR-99021)	Yes	465.34	1.85	5	3	115.2	7	32	-	1.46E-03	Moderately soluble	0.55
Alsterpaullon e	Yes	293.28	1.27	3	2	90.71	1	22	-	7.32E-02	Soluble	0.55
CHIR-98014	Yes	486.31	1.27	6	3	152.39	8	33	NorO>10	1.67E-03	Moderately soluble	0.55
SB216763	Yes	371.22	3.6	2	1	51.1	2	25	-	4.56E-03	Moderately soluble	0.55
AR-A014418	Yes	308.31	0.12	5	2	137.31	7	21	-	1.51E-01	Soluble	0.55

Doxorubicin	No	543.52	-2.1	12	6	206.07	5	39	MW>500, NorO>10, NHorOH>5	6.72E-02	Soluble	0.17
Paclitaxel	No	853.91	1.7	14	4	221.29	15	62	MW>500, NorO>10	1.85E-04	Poorly soluble	0.17

*The data was obtained from http://www.swissadme.ch/ website (HBA: Hydrogen bond donor, HBD: Hydrogen bond acceptor, TPSA: Topological polar surface area, RB: Rotable bond, natom: number of heavy atom, ESOL: Estimating Aqueous Solubility Directly from Molecular Structure)

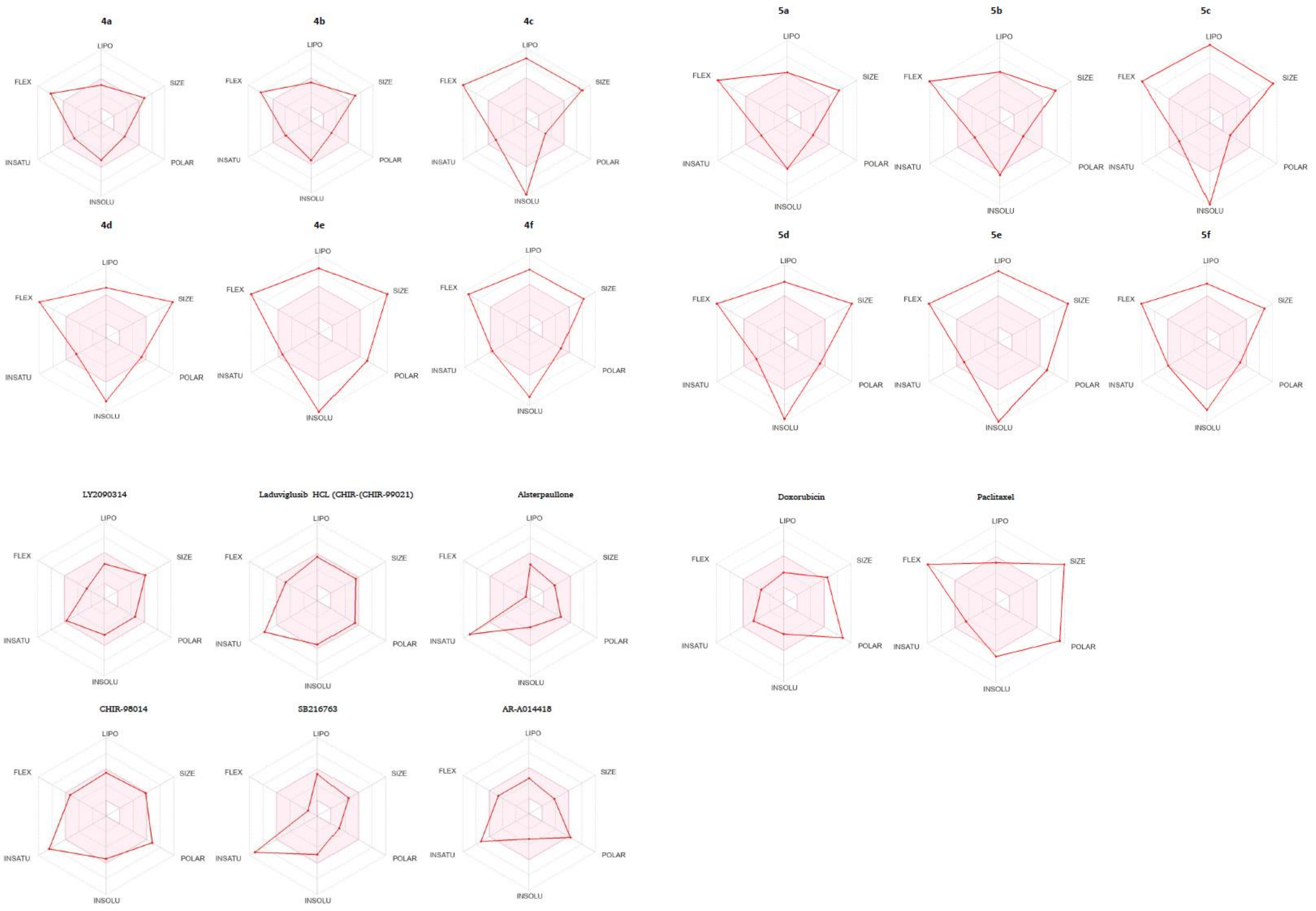

Figure S66. Bioavailability radar diagram of the synthesized compounds, some of the GSK-3B inhibitor drugs and some of the breast cancer drugs

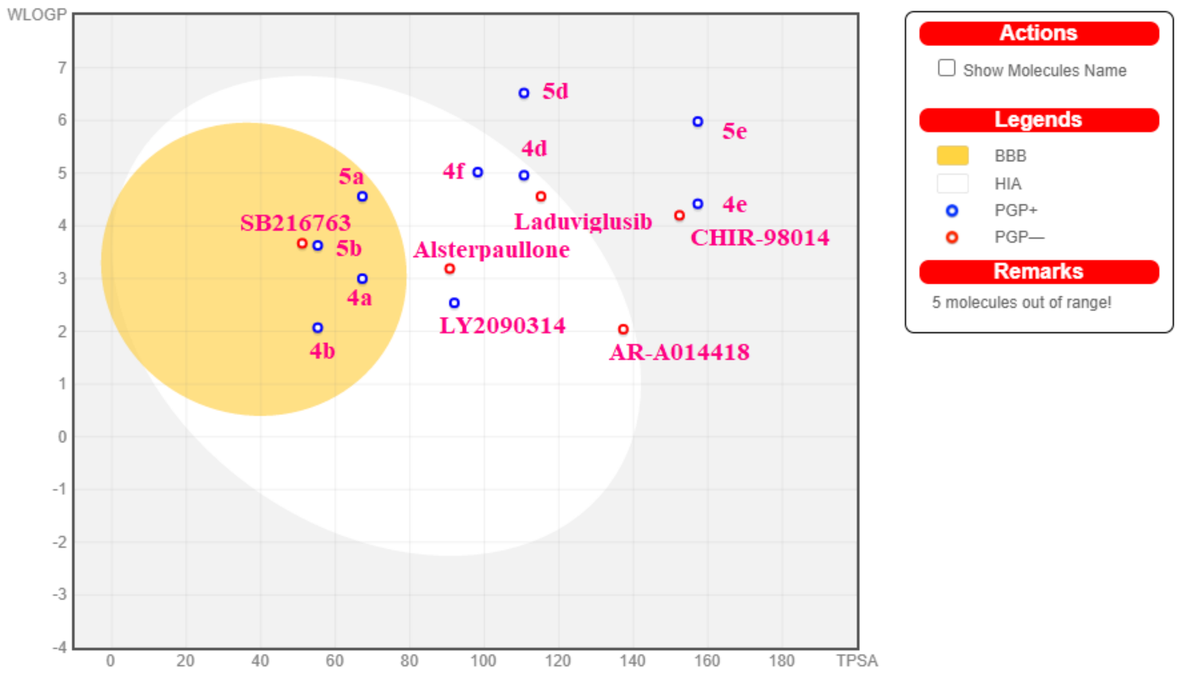

Figure S67. Prediction of permeation of the synthesized compounds by BOILED-Egg (The Brain Or IntestinaL EstimateD) method from http://www.swissadme.ch/ website. (BBB: Blood-Brain Barrier (yellow zone)., HIA: Human Intestinal Absorption (white zone), PGP: Permeability glycoprotein, PGP substrate (PGP+) (blue dot) and PGP non-substrate (PGP-) (red dot)).

Table S2. Phase 1 metabolic prediction of the synthesized compounds.

Comp.	GI absorption	BBB permeant	Pgp substrate	CYP1A2 inhibitor	CYP2C19 inhibitor	CYP2C9 inhibitor	CYP2D6 inhibitor	CYP3A4 inhibitor	log Kp (cm/s)
4a	High	Yes	Yes	Yes	Yes	Yes	Yes	Yes	-7.28
4b	High	Yes	Yes	Yes	Yes	No	Yes	Yes	-7.18
4c	Low	No	Yes	No	Yes	No	No	Yes	-3.99
4d	High	No	Yes	No	No	No	No	No	-7.20
4e	Low	No	Yes	No	No	Yes	No	No	-5.14
4f	High	No	Yes	No	No	Yes	No	Yes	-4.54
5a	High	Yes	Yes	Yes	Yes	No	Yes	Yes	-6.61
5b	High	Yes	Yes	Yes	No	No	Yes	Yes	-6.51
5c	Low	No	Yes	No	No	No	No	Yes	-3.32
5d	Low	No	Yes	No	No	No	No	No	-6.53
5e	Low	No	Yes	No	No	No	No	No	-4.46
5f	Low	No	No	No	No	No	No	No	-5.34
LY2090314	High	No	Yes	No	Yes	Yes	Yes	No	-7.67
Laduviglusib HCl (CHIR-99021)	High	No	No	Yes	Yes	Yes	Yes	Yes	-6.09
Alsterpaullone	High	No	No	Yes	No	No	Yes	Yes	-6.39
CHIR-98014	Low	No	No	Yes	Yes	Yes	No	Yes	-6.31
SB216763	High	Yes	No	Yes	Yes	Yes	No	Yes	-5.80
AR-A014418	High	No	No	Yes	Yes	No	No	No	-6.34
Doxorubicin	Low	No	Yes	No	No	No	No	No	-8.71
Paclitaxel	Low	No	Yes	No	No	No	No	No	-8.91

*The data was obtained from http://www.swissadme.ch/ website; log Kp: Human skin permeability coefficients.

Table S3. Predicted Toxicity Class of the Compounds

Predicted Toxicity Class	
**Class-3**	4c, 5b, CHIR98014, Doxorubicin, Paclitaxel
**Class-4**	4a, 4b, 4d, 4e, 4f, 5a, 5c, 5d, 5e, 5f, LY2090314, Laduviglusib HCl (CHIR-99021), Alsterpaullone, SB216763, AR-A014418

*The data was obtained from https://tox-new.charite.de/protox_II/index.php?site=compound_input (Class 3: toxic if swallowed (50 < LD50 ≤ 300), Class 4: harmful if swallowed (300 < LD50 ≤ 2000))

Table S4. Toxicity Model Report of the Compounds

Toxicity Model Report				
**Comp.**	**Classification**	**Target**	**Prediction**	**Probability**
**4a**	**Organ Toxicity**	Hepatotoxicity	Inactive	0.83
	**Toxicity end points**	Carcinogenicity	Inactive	0.53
		Immunotoxicity	Inactive	0.95
		Mutagenicity	Inactive	0.64
		Cytotoxicity	Inactive	0.64
	**Tox21 Nuclear receptor signalling pathways**	Aromatase	Inactive	0.92
		Estrogen Receptor Alpha (ER)	Inactive	0.87
		Estrogen Receptor Ligand Binding Domain (ER-LBD)	Inactive	0.97
	**Tox21-Stress response pathways**	Phosphoprotein (Tumor Supressor) p53	Inactive	0.86
**4b**	**Organ Toxicity**	Hepatotoxicity	Inactive	0.88
	**Toxicity end points**	Carcinogenicity	Inactive	0.63
		Immunotoxicity	Inactive	0.94
		Mutagenicity	Active	0.61
		Cytotoxicity	Inactive	0.58
	**Tox21 Nuclear receptor signalling pathways**	Aromatase	Inactive	0.96
		Estrogen Receptor Alpha (ER)	Inactive	0.94
		Estrogen Receptor Ligand Binding Domain (ER-LBD)	Inactive	0.98
	**Tox21-Stress response pathways**	Phosphoprotein (Tumor Supressor) p53	Inactive	0.89
**4c**	**Organ Toxicity**	Hepatotoxicity	Inactive	0.90
	**Toxicity end points**	Carcinogenicity	Inactive	0.59
		Immunotoxicity	Inactive	0.94
		Mutagenicity	Inactive	0.59
		Cytotoxicity	Inactive	0.70
	**Tox21 Nuclear receptor signalling pathways**	Aromatase	Inactive	0.96
		Estrogen Receptor Alpha (ER)	Inactive	0.94
		Estrogen Receptor Ligand Binding Domain (ER-LBD)	Inactive	0.98
	**Tox21-Stress response pathways**	Phosphoprotein (Tumor Supressor) p53	Inactive	0.89
**4d**	**Organ Toxicity**	Hepatotoxicity	Inactive	0.79
	**Toxicity end points**	Carcinogenicity	Active	0.53
		Immunotoxicity	Active	0.98
		Mutagenicity	Inactive	0.65
		Cytotoxicity	Inactive	0.55
	**Tox21 Nuclear receptor signalling pathways**	Aromatase	Inactive	0.90
		Estrogen Receptor Alpha (ER)	Inactive	0.91
		Estrogen Receptor Ligand Binding Domain (ER-LBD)	Inactive	0.98
	**Tox21-Stress response pathways**	Phosphoprotein (Tumor Supressor) p53	Inactive	0.86
**4e**	**Organ Toxicity**	Hepatotoxicity	Inactive	0.55
	**Toxicity end points**	Carcinogenicity	Active	0.51
		Immunotoxicity	Inactive	0.98
		Mutagenicity	Active	0.52
		Cytotoxicity	Inactive	0.58
	**Tox21 Nuclear receptor signalling pathways**	Aromatase	Inactive	0.92
		Estrogen Receptor Alpha (ER)	Inactive	0.94
		Estrogen Receptor Ligand Binding Domain (ER-LBD)	Inactive	0.97
	**Tox21-Stress response pathways**	Phosphoprotein (Tumor Supressor) p53	Inactive	0.91

**4f**	**Organ Toxicity**	Hepatotoxicity	Inactive	0.69
	**Toxicity end points**	Carcinogenicity	Active	0.57
		Immunotoxicity	Inactive	0.99
		Mutagenicity	Active	0.58
		Cytotoxicity	Inactive	0.53
	**Tox21 Nuclear receptor signalling pathways**	Aromatase	Inactive	0.94
		Estrogen Receptor Alpha (ER)	Inactive	0.94
		Estrogen Receptor Ligand Binding Domain (ER-LBD)	Inactive	0.97
	**Tox21-Stress response pathways**	Phosphoprotein (Tumor Supressor) p53	Inactive	0.88
**5a**	**Organ Toxicity**	Hepatotoxicity	Inactive	0.83
	**Toxicity end points**	Carcinogenicity	Inactive	0.53
		Immunotoxicity	Inactive	0.92
		Mutagenicity	Inactive	0.64
		Cytotoxicity	Inactive	0.64
	**Tox21 Nuclear receptor signalling pathways**	Aromatase	Inactive	0.92
		Estrogen Receptor Alpha (ER)	Inactive	0.87
		Estrogen Receptor Ligand Binding Domain (ER-LBD)	Inactive	0.97
	**Tox21-Stress response pathways**	Phosphoprotein (Tumor Supressor) p53	Inactive	0.86
**5b**	**Organ Toxicity**	Hepatotoxicity	Inactive	0.88
	**Toxicity end points**	Carcinogenicity	Inactive	0.63
		Immunotoxicity	Inactive	0.89
		Mutagenicity	Active	0.61
		Cytotoxicity	Inactive	0.58
	**Tox21 Nuclear receptor signalling pathways**	Aromatase	Inactive	0.96
		Estrogen Receptor Alpha (ER)	Inactive	0.94
		Estrogen Receptor Ligand Binding Domain (ER-LBD)	Inactive	0.98
	**Tox21-Stress response pathways**	Phosphoprotein (Tumor Supressor) p53	Inactive	0.89
**5c**	**Organ Toxicity**	Hepatotoxicity	Inactive	0.90
	**Toxicity end points**	Carcinogenicity	Inactive	0.59
		Immunotoxicity	Inactive	0.90
		Mutagenicity	Inactive	0.59
		Cytotoxicity	Inactive	0.70
	**Tox21 Nuclear receptor signalling pathways**	Aromatase	Inactive	0.96
		Estrogen Receptor Alpha (ER)	Inactive	0.94
		Estrogen Receptor Ligand Binding Domain (ER-LBD)	Inactive	0.98
	**Tox21-Stress response pathways**	Phosphoprotein (Tumor Supressor) p53	Inactive	0.89
**5d**	**Organ Toxicity**	Hepatotoxicity	Inactive	0.79
	**Toxicity end points**	Carcinogenicity	Active	0.53
		Immunotoxicity	Active	0.99
		Mutagenicity	Inactive	0.65
		Cytotoxicity	Inactive	0.55
	**Tox21 Nuclear receptor signalling pathways**	Aromatase	Inactive	0.90
		Estrogen Receptor Alpha (ER)	Inactive	0.91
		Estrogen Receptor Ligand Binding Domain (ER-LBD)	Inactive	0.98
	**Tox21-Stress response pathways**	Phosphoprotein (Tumor Supressor) p53	Inactive	0.86
**5e**	**Organ Toxicity**	Hepatotoxicity	Inactive	0.57
	**Toxicity end points**	Carcinogenicity	Active	0.51
		Immunotoxicity	Inactive	0.97
		Mutagenicity	Active	0.52

		Cytotoxicity	Inactive	0.58
	**Tox21 Nuclear receptor signalling pathways**	Aromatase	Inactive	0.92
		Estrogen Receptor Alpha (ER)	Inactive	0.94
		Estrogen Receptor Ligand Binding Domain (ER-LBD)	Inactive	0.97
	**Tox21-Stress response pathways**	Phosphoprotein (Tumor Supressor) p53	Inactive	0.90
**5f**	**Organ Toxicity**	Hepatotoxicity	Inactive	0.66
	**Toxicity end points**	Carcinogenicity	Active	0.51
		Immunotoxicity	Inactive	0.97
		Mutagenicity	Active	0.53
		Cytotoxicity	Inactive	0.55
	**Tox21 Nuclear receptor signalling pathways**	Aromatase	Inactive	0.94
		Estrogen Receptor Alpha (ER)	Inactive	0.94
		Estrogen Receptor Ligand Binding Domain (ER-LBD)	Inactive	0.97
	**Tox21-Stress response pathways**	Phosphoprotein (Tumor Supressor) p53	Inactive	0.87
LY2090314	**Organ Toxicity**	Hepatotoxicity	Inactive	0.78
	**Toxicity end points**	Carcinogenicity	Inactive	0.71
		Immunotoxicity	Active	0.71
		Mutagenicity	Inactive	0.53
		Cytotoxicity	Inactive	0.71
	**Tox21 Nuclear receptor signalling pathways**	Aromatase	Inactive	0.96
		Estrogen Receptor Alpha (ER)	Inactive	0.94
		Estrogen Receptor Ligand Binding Domain (ER-LBD)	Inactive	0.97
	**Tox21-Stress response pathways**	Phosphoprotein (Tumor Supressor) p53	Inactive	0.92
Laduviglusib HCl (CHIR-99021)	**Organ Toxicity**	Hepatotoxicity	Inactive	0.70
	**Toxicity end points**	Carcinogenicity	Inactive	0.68
		Immunotoxicity	Inactive	0.69
		Mutagenicity	Inactive	0.75
		Cytotoxicity	Inactive	0.65
	**Tox21 Nuclear receptor signalling pathways**	Aromatase	Inactive	0.82
		Estrogen Receptor Alpha (ER)	Inactive	0.78
		Estrogen Receptor Ligand Binding Domain (ER-LBD)	Inactive	0.95
	**Tox21-Stress response pathways**	Phosphoprotein (Tumor Supressor) p53	Inactive	0.89
Alsterpaullone	**Organ Toxicity**	Hepatotoxicity	Inactive	0.55
	**Toxicity end points**	Carcinogenicity	Active	0.68
		Immunotoxicity	Inactive	0.57
		Mutagenicity	Active	0.78
		Cytotoxicity	Inactive	0.67
	**Tox21 Nuclear receptor signalling pathways**	Aromatase	Inactive	0.92
		Estrogen Receptor Alpha (ER)	Inactive	0.87
		Estrogen Receptor Ligand Binding Domain (ER-LBD)	Inactive	0.95
	**Tox21-Stress response pathways**	Phosphoprotein (Tumor Supressor) p53	Inactive	0.84
CHIR-98014	**Organ Toxicity**	Hepatotoxicity	Inactive	0.58
	**Toxicity end points**	Carcinogenicity	Active	0.57
		Immunotoxicity	Active	0.98
		Mutagenicity	Active	0.79
		Cytotoxicity	Inactive	0.57
	**Tox21 Nuclear receptor signalling pathways**	Aromatase	Inactive	0.73
		Estrogen Receptor Alpha (ER)	Inactive	0.83

		Estrogen Receptor Ligand Binding Domain (ER-LBD)	Inactive	0.84
	**Tox21-Stress response pathways**	Phosphoprotein (Tumor Supressor) p53	Inactive	0.85
SB216763	**Organ Toxicity**	Hepatotoxicity	Inactive	0.51
	**Toxicity end points**	Carcinogenicity	Inactive	0.56
		Immunotoxicity	Inactive	0.83
		Mutagenicity	Inactive	0.58
		Cytotoxicity	Inactive	0.77
	**Tox21 Nuclear receptor signalling pathways**	Aromatase	Inactive	0.91
		Estrogen Receptor Alpha (ER)	Inactive	0.83
		Estrogen Receptor Ligand Binding Domain (ER-LBD)	Inactive	0.94
	**Tox21-Stress response pathways**	Phosphoprotein (Tumor Supressor) p53	Inactive	0.75
AR-A014418	**Organ Toxicity**	Hepatotoxicity	Inactive	0.58
	**Toxicity end points**	Carcinogenicity	Active	0.73
		Immunotoxicity	Inactive	0.94
		Mutagenicity	Active	0.88
		Cytotoxicity	Inactive	0.70
	**Tox21 Nuclear receptor signalling pathways**	Aromatase	Inactive	0.94
		Estrogen Receptor Alpha (ER)	Inactive	0.63
		Estrogen Receptor Ligand Binding Domain (ER-LBD)	Inactive	0.96
	**Tox21-Stress response pathways**	Phosphoprotein (Tumor Supressor) p53	Inactive	0.79
Doxorubicin	**Organ Toxicity**	Hepatotoxicity	Inactive	0.86
	**Toxicity end points**	Carcinogenicity	Inactive	0.90
		Immunotoxicity	Active	0.99
		Mutagenicity	Active	0.98
		Cytotoxicity	Active	0.94
	**Tox21 Nuclear receptor signalling pathways**	Aromatase	Active	0.52
		Estrogen Receptor Alpha (ER)	Inactive	0.73
		Estrogen Receptor Ligand Binding Domain (ER-LBD)	Inactive	0.74
	**Tox21-Stress response pathways**	Phosphoprotein (Tumor Supressor) p53	Active	0.52
Paclitaxel	**Organ Toxicity**	Hepatotoxicity	Inactive	0.63
	**Toxicity end points**	Carcinogenicity	Inactive	0.61
		Immunotoxicity	Active	0.99
		Mutagenicity	Inactive	0.85
		Cytotoxicity	Active	0.72
	**Tox21 Nuclear receptor signalling pathways**	Aromatase	Inactive	0.95
		Estrogen Receptor Alpha (ER)	Inactive	0.87
		Estrogen Receptor Ligand Binding Domain (ER-LBD)	Inactive	0.98
	**Tox21-Stress response pathways**	Phosphoprotein (Tumor Supressor) p53	Active	0.79

*The data was obtained from https://tox-new.charite.de/protox_II/index.php?site=compound_input (Inactive and probability above 0.70 = no risk or low risk (dark green); inactive and probability below 0.70 =medium risk (light green); active and probability below 0.70 = high risk (pink); active and probability above 0.70 = very high risk (red))

Table S5. Similartiy Ensambles Aproach of the Compounds

Compound	Target	Definition	P-value	Max TC
4a	PRKCB	Protein kinase C beta type	2.425e-33	0.48
	PRKCG	Protein kinase C gamma type	6.958e-31	0.46
	CAMK2D	Calcium/calmodulin-dependent protein kinase type II subunit delta	1.603e-30	0.49
	TACR2	Substance-K receptor	3.246e-30	0.37
	PRKCA	Protein kinase C alpha type	2.267e-28	0.48
	PRKCE	Protein kinase C epsilon type	1.289e-24	0.47
	HTR7	5-hydroxytryptamine receptor 7	3.253e-24	0.44
	GSK3B	Glycogen synthase kinase-3 beta	6.739e-24	0.53
	CNR2	Cannabinoid receptor 2	1.327e-23	0.49
	HRH2	Histamine H2 receptor	1.514e-23	0.35
	PRKCH	Protein kinase C eta type	2.243e-20	0.43
	TRPV4	Transient receptor potential cation channel subfamily V member 4	9.315e-20	0.35
	ICMT	Protein-S-isoprenylcysteine O-methyltransferase	1.11e-16	0.38
	AVPR1B	Vasopressin V1b receptor	3.331e-16	0.35
4b	HTR7	5-hydroxytryptamine receptor 7	4.477e-46	0.43
	PRKCB	Protein kinase C beta type	1.974e-40	0.59
	PRKCA	Protein kinase C alpha type	5.963e-39	0.59
	PRKCE	Protein kinase C epsilon type	2.204e-35	0.48
	CAMK2D	Calcium/calmodulin-dependent protein kinase type II subunit delta	8.885e-34	0.50
	PRKCH	Protein kinase C eta type	6.265e-33	0.42
	PRKCG	Protein kinase C gamma type	2.276e-32	0.42
	HRH2	Histamine H2 receptor	2.777e-26	0.36
	HTR1A	5-hydroxytryptamine receptor 1A	2.842e-24	0.42
	GSK3B	Glycogen synthase kinase-3 beta	1.374e-23	0.55
	HTR6	5-hydroxytryptamine receptor 6	4.449e-23	0.39
	CNR2	Cannabinoid receptor 2	3.034e-21	0.40
	DRD3	D(3) dopamine receptor	1.435e-19	0.42
	EBPL	Emopamil-binding protein-like	6.741e-19	0.30
	PRKACB	cAMP-dependent protein kinase catalytic subunit beta	9.923e-18	0.32
	DRD2	D(2) dopamine receptor	1.102e-17	0.42
	ICMT	Protein-S-isoprenylcysteine O-methyltransferase	3.099e-17	0.37
	DRD5	D(1B) dopamine receptor	1.11e-16	0.37
	PRKCD	Protein kinase C delta type	1.11e-16	0.42
4c	CCR3			
	HRH2	C-C chemokine receptor type 3	2.93e-43	0.46
	GRIN2B	Histamine H2 receptor	4.263e-36	0.38
	CAMK2D	Glutamate receptor ionotropic, NMDA 2B	1.995e-34	0.41
	DCUN1D1	Calcium/calmodulin-dependent protein kinase type II subunit delta	7.139e-31	0.46
	PRKCB	DCN1-like protein 1	5.304e-28	0.30
	PRKCH	Protein kinase C beta type	3.103e-27	0.45
	PRKCE	Protein kinase C eta type	9.508e-26	0.38
	HTR4	Protein kinase C epsilon type	2.11e-25	0.44
	PRKCA	5-hydroxytryptamine receptor 4	4.584e-25	0.35
	PRMT6	Protein kinase C alpha type	3.725e-24	0.45
	PRKCG	Protein arginine N-methyltransferase 6	5.212e-22	0.40
	HTR7	Protein kinase C gamma type	5.832e-22	0.38
	SIGMAR1	5-hydroxytryptamine receptor 7	8.358e-22	0.50
	GRIN1	Sigma non-opioid intracellular receptor 1	2.36e-20	0.46
	PRKACB	Glutamate receptor ionotropic, NMDA 1	7.117e-20	0.36
	TMEM97	cAMP-dependent protein kinase catalytic subunit beta	5.339e-18	0.32
	CARM1	Sigma intracellular receptor 2	6.39e-18	0.37
	PDE6D	Histone-arginine methyltransferase CARM1	3.331e-16	0.40
		Retinal rod rhodopsin-sensitive cGMP 3',5'-cyclic phosphodiesterase subur	4.441e-16	0.32

4d	SLC6A13	Sodium- and chloride-dependent GABA transporter 2	2.276e-38	0.30
	HTR7	5-hydroxytryptamine receptor 7	3.388e-34	0.41
	HRH2	Histamine H2 receptor	8.983e-32	0.38
	DRD3	D(3) dopamine receptor	1.511e-28	0.42
	CAMK2D	Calcium/calmodulin-dependent protein kinase type II subunit delta	5.605e-27	0.41
	DRD2	D(2) dopamine receptor	6.066e-24	0.42
	HTR1A	5-hydroxytryptamine receptor 1A	4.852e-22	0.42
	DRD4	D(4) dopamine receptor	7.885e-21	0.36
	PRKCB	Protein kinase C beta type	2.722e-20	0.45
	EBPL	Emopamil-binding protein-like	1.372e-18	0.29
	ADRA1B	Alpha-1B adrenergic receptor	1.11e-16	0.41
	PRKCA	Protein kinase C alpha type	2.22e-16	0.45
	ADRA1A	Alpha-1A adrenergic receptor	5.551e-16	0.41
	PRKCE	Protein kinase C epsilon type	5.551e-16	0.42
	SLC6A11	Sodium- and chloride-dependent GABA transporter 3	5.551e-16	0.30
4e	KCC2D	Calcium/calmodulin-dependent protein kinase type II subunit delta	6.774e-26	0.42
	KPCA	Protein kinase C alpha type	5.695e-14	0.38
	KPCB	Protein kinase C beta type	3.27e-11	0.38
	GSK3B	Glycogen synthase kinase-3 beta	1.508e-10	0.36
	KPCE	Protein kinase C epsilon type	5.085e-10	0.38
4f	CAMK2D	Calcium/calmodulin-dependent protein kinase type II subunit delta	1.978e-27	0.45
	PRKCA	Protein kinase C alpha type	9.209e-13	0.38
	PRKCB	Protein kinase C beta type	2.018e-12	0.38
	PRKCE	Protein kinase C epsilon type	9.223e-12	0.38
	GSK3B	Glycogen synthase kinase-3 beta	3.05e-10	0.35
	CNR2	Cannabinoid receptor 2	4.099e-07	0.37
5a	PRKCB	Protein kinase C beta type	1.844e-33	0.47
	CAMK2D	Calcium/calmodulin-dependent protein kinase type II subunit delta	1.543e-30	0.48
	PRKCG	Protein kinase C gamma type	1.94e-30	0.45
	HTR7	5-hydroxytryptamine receptor 7	1.211e-29	0.46
	TACR2	Substance-K receptor	3.068e-28	0.37
	PRKCA	Protein kinase C alpha type	1.331e-27	0.47
	PRKCE	Protein kinase C epsilon type	3.886e-25	0.46
	ICMT	Protein-S-isoprenylcysteine O-methyltransferase	9.415e-23	0.39
	GSK3B	Glycogen synthase kinase-3 beta	2.213e-22	0.52
	CNR2	Cannabinoid receptor 2	4.016e-22	0.48
	HRH2	Histamine H2 receptor	1.443e-21	0.34
	PRKCH	Protein kinase C eta type	3.625e-21	0.42
	DNM2	Dynamin-2	5.099e-21	0.28
	TRPV4	Transient receptor potential cation channel subfamily V member 4	1.62e-19	0.35
5b	HTR7	5-hydroxytryptamine receptor 7	8.666e-51	0.45
	PRKCB	Protein kinase C beta type	4.882e-40	0.58
	PRKCA	Protein kinase C alpha type	1.183e-38	0.58
	PRKCE	Protein kinase C epsilon type	6.222e-36	0.47
	PRKCH	Protein kinase C eta type	8.435e-35	0.41
	CAMK2D	Calcium/calmodulin-dependent protein kinase type II subunit delta	1.303e-34	0.49
	PRKCG	Protein kinase C gamma type	3.18e-31	0.41
	ICMT	Protein-S-isoprenylcysteine O-methyltransferase	2.405e-28	0.39
	HTR1A	5-hydroxytryptamine receptor 1A	8.894e-27	0.41
	DNM2	Dynamin-2	2.017e-26	0.29
	HRH2	Histamine H2 receptor	4.1e-25	0.36
	GSK3B	Glycogen synthase kinase-3 beta	1.781e-22	0.55
	HTR6	5-hydroxytryptamine receptor 6	4.288e-22	0.38
	CNR2	Cannabinoid receptor 2	6.472e-20	0.39
	EBPL	Emopamil-binding protein-like	1.211e-18	0.30
	DRD2	D(2) dopamine receptor	1.377e-17	0.41

	PRKCD	Protein kinase C delta type	1.494e-17	0.41
	PRKACB	cAMP-dependent protein kinase catalytic subunit beta	1.668e-17	0.32
	DRD3	D(3) dopamine receptor	1.11e-16	0.41
5c	CCR3	C-C chemokine receptor type 3	1.048e-41	0.46
	HRH2	Histamine H2 receptor	7.213e-35	0.37
	GRIN2B	Glutamate receptor ionotropic, NMDA 2B	6.332e-32	0.40
	CAMK2D	Calcium/calmodulin-dependent protein kinase type II subunit delta	6.948e-31	0.45
	HTR7	5-hydroxytryptamine receptor 7	5.024e-26	0.51
	PRKCB	Protein kinase C beta type	7.118e-26	0.44
	PRMT6	Protein arginine N-methyltransferase 6	9.478e-26	0.40
	DCUN1D1	DCN1-like protein 1	4.765e-25	0.29
	PRKCH	Protein kinase C eta type	1.869e-24	0.38
	PRKCE	Protein kinase C epsilon type	1.308e-23	0.44
	HTR4	5-hydroxytryptamine receptor 4	7.642e-23	0.35
	PRKCA	Protein kinase C alpha type	3.131e-22	0.44
	PRKCG	Protein kinase C gamma type	6.076e-21	0.38
	SIGMAR1	Sigma non-opioid intracellular receptor 1	2.122e-20	0.46
	CARM1	Histone-arginine methyltransferase CARM1	2.766e-19	0.40
	GRIN1	Glutamate receptor ionotropic, NMDA 1	1.626e-18	0.36
	TMEM97	Sigma intracellular receptor 2	7.169e-18	0.38
	PRKACB	cAMP-dependent protein kinase catalytic subunit beta	8.829e-18	0.32
	HTR1A	5-hydroxytryptamine receptor 1A	2.22e-16	0.40
5d	SLC6A13	Sodium- and chloride-dependent GABA transporter 2	6.694e-38	0.30
	HTR7	5-hydroxytryptamine receptor 7	1.217e-37	0.40
	HRH2	Histamine H2 receptor	1.449e-30	0.38
	DRD3	D(3) dopamine receptor	8.058e-29	0.42
	CAMK2D	Calcium/calmodulin-dependent protein kinase type II subunit delta	2.827e-26	0.41
	DRD2	D(2) dopamine receptor	2.001e-25	0.42
	HTR1A	5-hydroxytryptamine receptor 1A	1.957e-24	0.42
	DRD4	D(4) dopamine receptor	1.153e-21	0.35
	PRKCB	Protein kinase C beta type	1.775e-18	0.44
	EBPL	Emopamil-binding protein-like	2.301e-18	0.29
	ADRA1B	Alpha-1B adrenergic receptor	6.661e-16	0.40
	SLC6A11	Sodium- and chloride-dependent GABA transporter 3	8.882e-16	0.30
5e	CAMK2D	Calcium/calmodulin-dependent protein kinase type II subunit delta	5.865e-26	0.42
	PRKCA	Protein kinase C alpha type	2.043e-13	0.39
	PRKCB	Protein kinase C beta type	4.112e-11	0.39
	PRKCE	Protein kinase C epsilon type	5.962e-10	0.39
	GSK3B	Glycogen synthase kinase-3 beta	7.921e-10	0.35
	DNM2	Dynamin-2	4.51e-06	0.30
5f	SIGLEC7	Sialic acid-binding Ig-like lectin 7	7.442e-47	0.29
	CAMK2D	Calcium/calmodulin-dependent protein kinase type II subunit delta	1.518e-30	0.48
	PTPN22	Tyrosine-protein phosphatase non-receptor type 22	1.824e-26	0.33
	PRKCA	Protein kinase C alpha type	3.997e-15	0.40
	HDAC8	Histone deacetylase 8	5.218e-15	0.48
	HDAC10	Polyamine deacetylase HDAC10	6.661e-14	0.36
	GSK3B	Glycogen synthase kinase-3 beta	3.048e-13	0.38
LY2090314	TGFBR2	TGF-beta receptor type-2	1.11e-15	0.69
	GSK3B	Glycogen synthase kinase-3 beta	2.622e-08	0.74
	PRKCB	Protein kinase C beta type	1.985e-07	0.74
Laduviglusib (CHIR-99021) HCl	CSNK1G1	Casein kinase I isoform gamma-1	0.0002285	1.00
	GSK3A	Glycogen synthase kinase-3 alpha	0.003073	1.00
	SLK	STE20-like serine/threonine-protein kinase	0.003442	1.00
	GSK3B	Glycogen synthase kinase-3 beta	0.00538	1.00
	TAOK1	Serine/threonine-protein kinase TAO1	0.01204	1.00
	LIMK1	LIM domain kinase 1	0.02709	1.00
	CLK4	Dual specificity protein kinase CLK4	0.132	1.00
	CDK1	Cyclin-dependent kinase 1	0.1912	1.00
	RPS6KB1	Ribosomal protein S6 kinase beta-1	0.6106	1.00
	CDK2	Cyclin-dependent kinase 2	0.8775	1.00
	CCR6	C-C chemokine receptor type 6	7.318e-23	0.28
	CNR1	Cannabinoid receptor 1	6.368e-07	0.35

	CCR6	C-C chemokine receptor type 6	8.833e-06	0.28
Alsterpaullone	GSK3B	Glycogen synthase kinase-3 beta	1.104e-24	1.00
	PASK	PAS domain-containing serine/threonine-protein kinase	9.774e-19	1.00
	CDK1	Cyclin-dependent kinase 1	2.613e-18	1.00
	TSSK2	Testis-specific serine/threonine-protein kinase 2	1.998e-15	1.00
	CDK5R1	Cyclin-dependent kinase 5 activator 1	4.678e-08	1.00
	CDC42BP	Serine/threonine-protein kinase MRCK alpha	2.815e-07	1.00
	CDK19	Cyclin-dependent kinase 19	4.195e-07	1.00
	CDK5	Cyclin-dependent-like kinase 5	3.661e-06	1.00
	MARK2	Serine/threonine-protein kinase MARK2	7.627e-06	1.00
	FLT4	Vascular endothelial growth factor receptor 3	1.136e-05	1.00
	PHKG2	Phosphorylase b kinase gamma catalytic chain, liver/testis isoform	1.363e-05	1.00
	MARK3	MAP/microtubule affinity-regulating kinase 3	4.674e-05	1.00
	CDK9	Cyclin-dependent kinase 9	5.096e-05	1.00
	ACVR1	Activin receptor type-1	9.519e-05	1.00
	STK3	Serine/threonine-protein kinase 3	0.0001157	1.00
	CDK2	Cyclin-dependent kinase 2	0.000136	1.00
	PLK4	Serine/threonine-protein kinase PLK4	0.0002264	1.00
	CSNK1G1	Casein kinase I isoform gamma-1	0.0002285	1.00
	CHEK2	Serine/threonine-protein kinase Chk2	0.0002632	1.00
	BRSK1	Serine/threonine-protein kinase BRSK1	0.0003039	1.00
CHIR-98014	GSK3A	Glycogen synthase kinase-3 alpha	0.0001501	1.00
	GSK3B	Glycogen synthase kinase-3 beta	0.003492	1.00
	CDK1	Cyclin-dependent kinase 1	0.04714	1.00
	PRKCQ	Protein kinase C theta type	1.509e-12	0.35
	SLC22A8	Solute carrier family 22 member 8	7.607e-06	0.28
SB216763	GSK3B	Glycogen synthase kinase-3 beta	1.47e-70	1.00
	CCNA1	Cyclin-A1	1.013e-07	1.00
	CDK2	Cyclin-dependent kinase 2	2.148e-07	1.00
	GSK3A	Glycogen synthase kinase-3 alpha	5.635e-06	1.00
	CCNA2	Cyclin-A2	2.834e-05	1.00
	CYP1A2	Cytochrome P450 1A2	0.002583	1.00
	CYP3A4	Cytochrome P450 3A4	0.01275	1.00
	PAX8	Paired box protein Pax-8	0.1668	1.00
AR-A014418	GSK3B	Glycogen synthase kinase-3 beta	4.238e-09	1.00
	CDK5	Cyclin-dependent-like kinase 5	7.895e-09	1.00
	GSK3A	Glycogen synthase kinase-3 alpha	2.018e-06	1.00
	AURKB	Aurora kinase B	2.646e-06	1.00
	CDK2	Cyclin-dependent kinase 2	0.0006467	1.00
	RPS6KB1	Ribosomal protein S6 kinase beta-1	0.004548	1.00
Doxorubicin	S100A4	Protein S100-A4	6.956e-99	1.00
	DHCR7	7-dehydrocholesterol reductase	3.545e-55	1.00
	YWHAG	14-3-3 protein gamma	4.48e-49	1.00
	TOP2B	DNA topoisomerase 2-beta	4.419e-25	1.00
	EBP	3-beta-hydroxysteroid-Delta(8),Delta(7)-isomerase	3.136e-18	1.00
	CISD1	CDGSH iron-sulfur domain-containing protein 1	4.222e-09	1.00
	TOP2A	DNA topoisomerase 2-alpha	4.062e-07	1.00
	PAX8	Paired box protein Pax-8	1.13e-06	1.00
	FYN	Tyrosine-protein kinase Fyn	4.104e-05	1.00
	MMP2	72 kDa type IV collagenase	0.0152	1.00
	CHRM1	Muscarinic acetylcholine receptor M1	0.6272	1.00
	ERBB2	Receptor tyrosine-protein kinase erbB-2	0.7112	1.00
	AURKA	Aurora kinase A	0.944	1.00
Paclitaxel	TUBB3	Tubulin beta-3 chain	4.629e-39	1.00
	NOD2	Nucleotide-binding oligomerization domain-containing protein 2	0.0004316	1.00
	MERTK	Tyrosine-protein kinase Mer	0.0008578	1.00
	TACR2	Substance-K receptor	0.008848	1.00
	CCKAR	Cholecystokinin receptor type A	0.01418	1.00
	ITGAV	Integrin alpha-V	0.08047	1.00
	ITGB3	Integrin beta-3	0.4299	1.00

	OPRD1	Delta-type opioid receptor	0.9836	1.00

*The data was obtained from https://sea.bkslab.org/

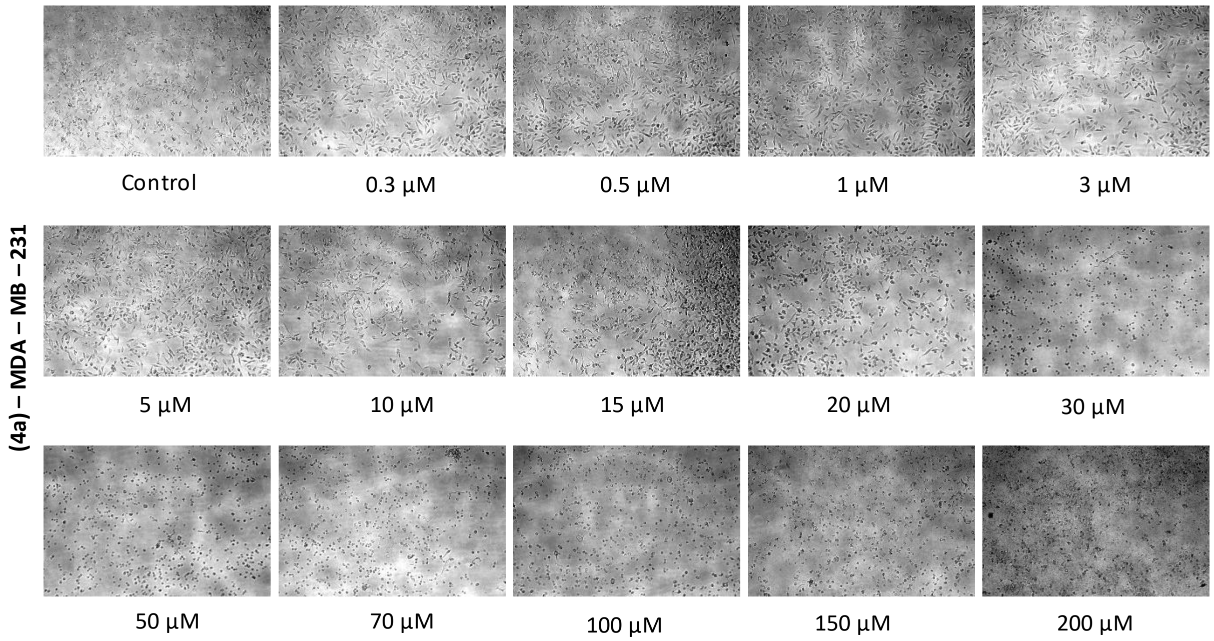

Figure S68. Microscope images of Compound **4a** in MDA-MB-231 cell line prepared at various concentrations

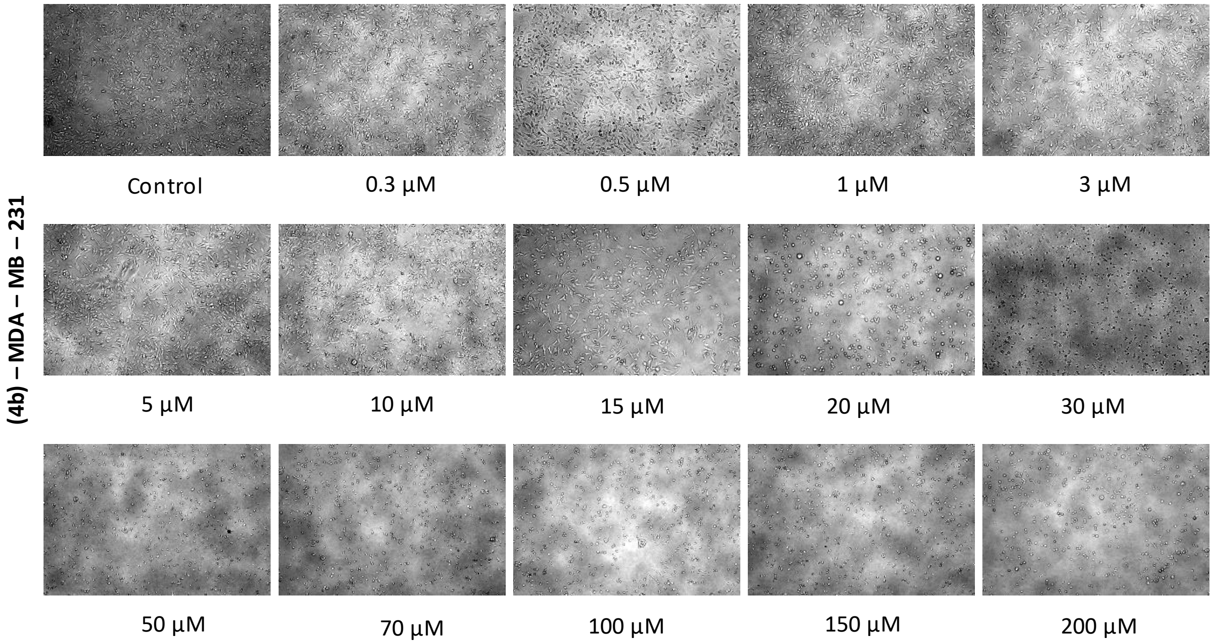

Figure S69. Microscope images of Compound **4b** in MDA-MB-231 cell line prepared at various concentrations

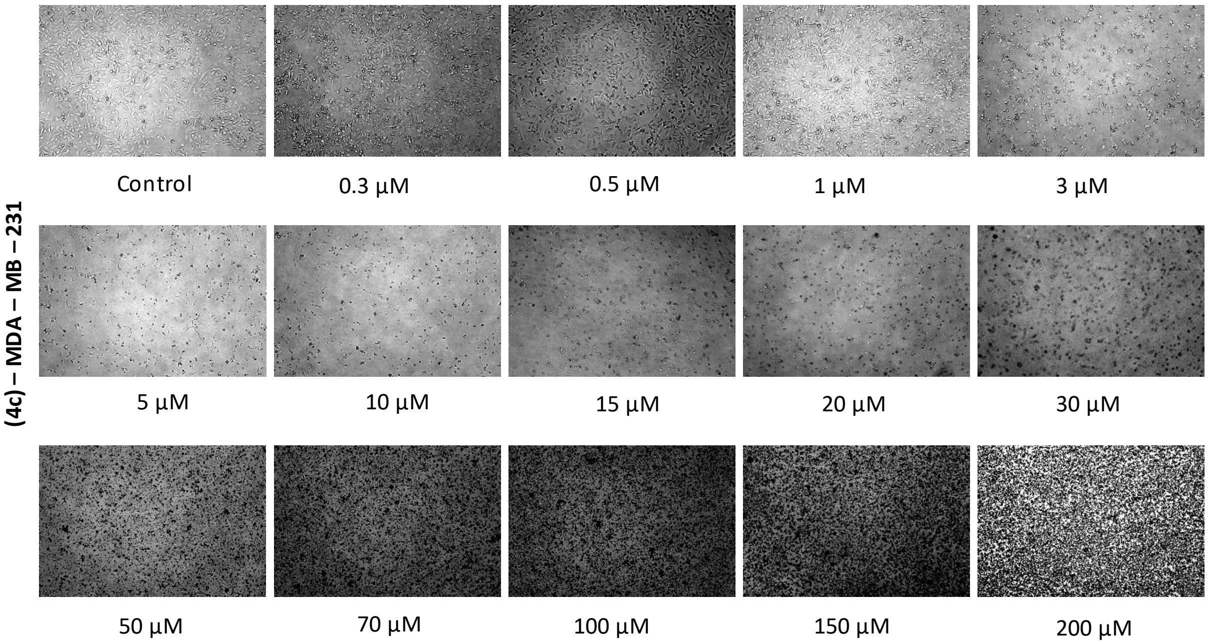

Figure S70. Microscope images of Compound **4c** in MDA-MB-231 cell line prepared at various concentration

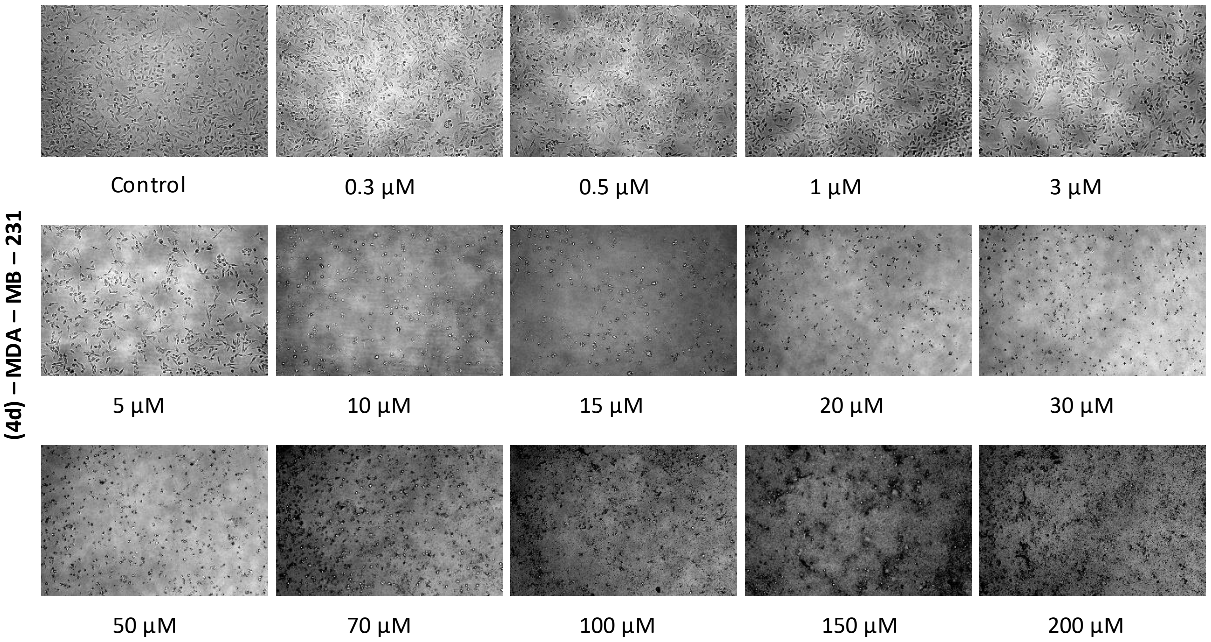

Figure S71. Microscope images of Compound **4d** in MDA-MB-231 cell line prepared at various concentrations

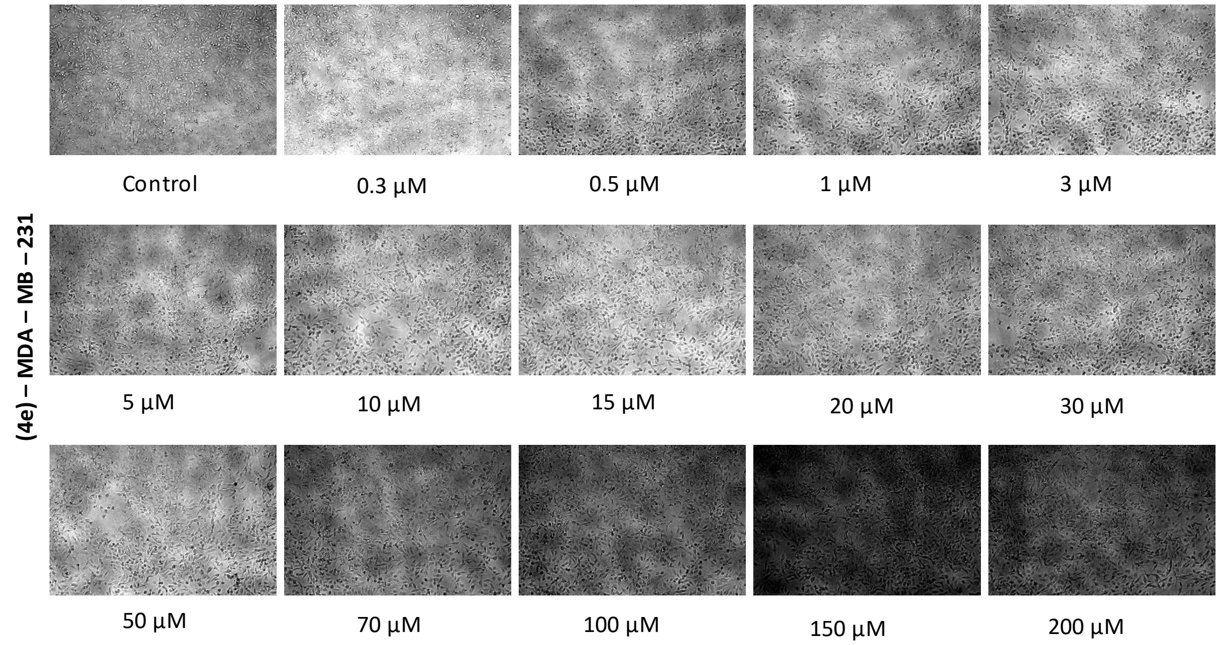

Figure S72. Microscope images of Compound **4e** in MDA-MB-231 cell line prepared at various concentrations

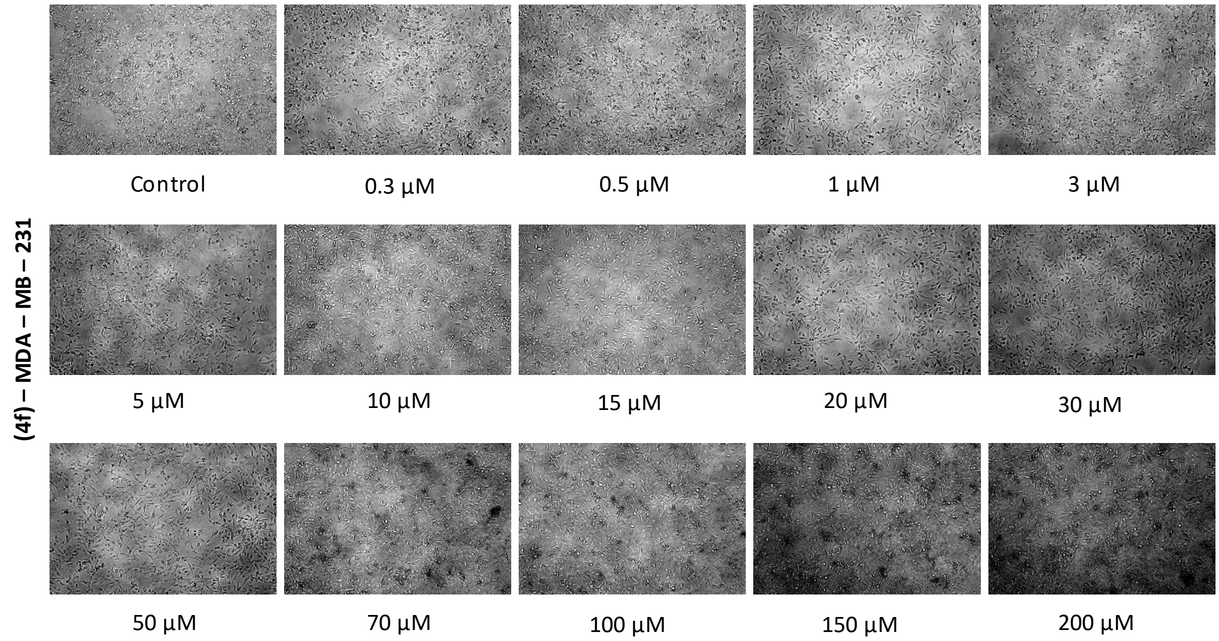

Figure S73. Microscope images of Compound **4f** in MDA-MB-231 cell line prepared at various concentrations

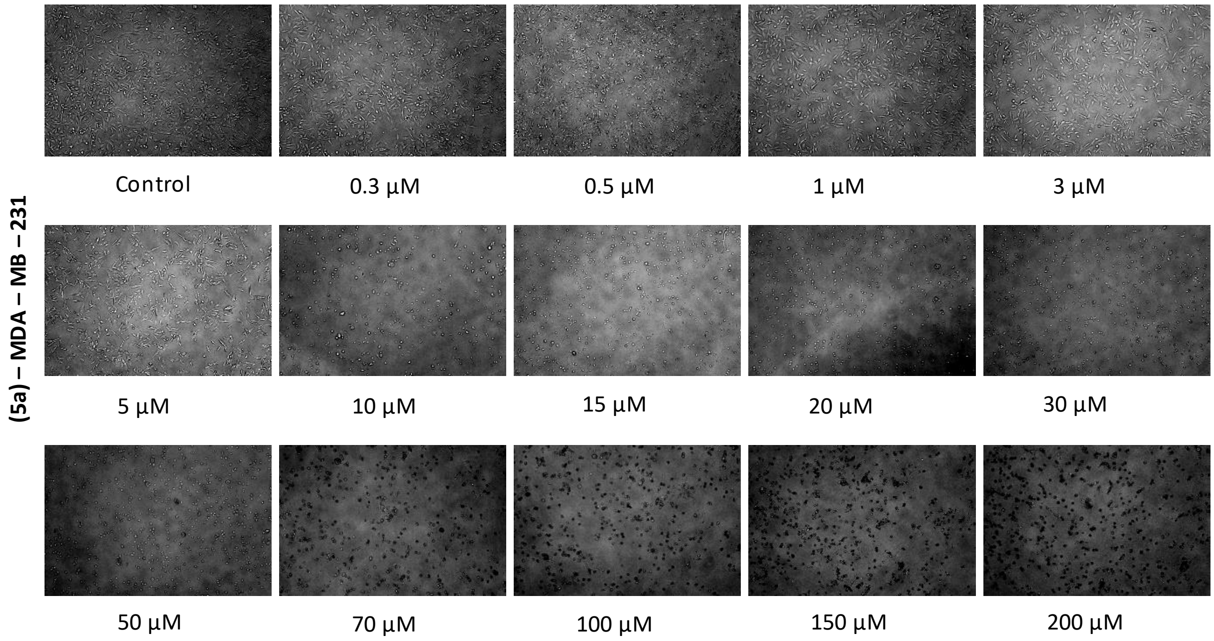

Figure S74. Microscope images of Compound **5a** in MDA-MB-231 cell line prepared at various concentrations

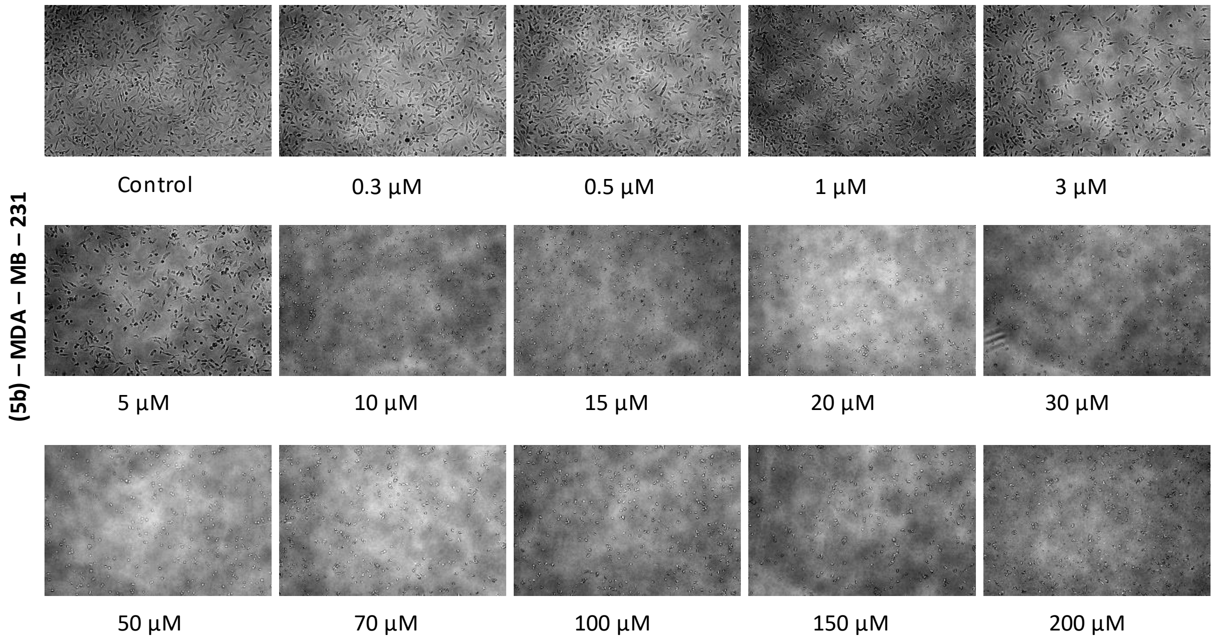

Figure S75. Microscope images of Compound **5b** in MDA-MB-231 cell line prepared at various concentrations

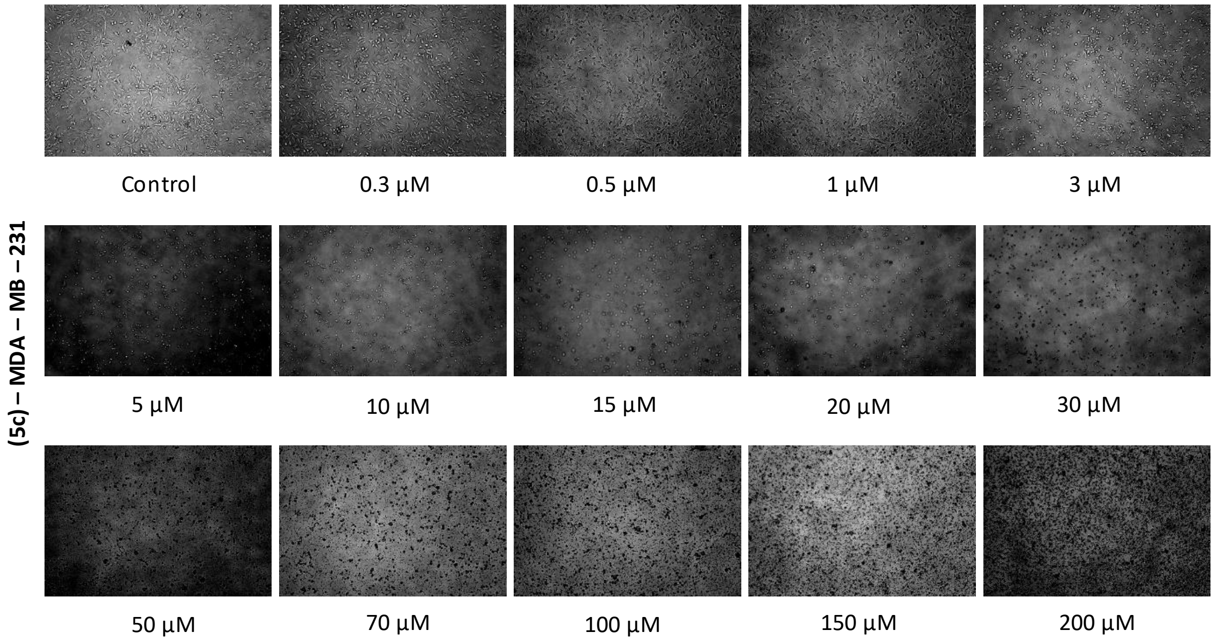

Figure S76. Microscope images of Compound **5c** in MDA-MB-231 cell line prepared at various concentrations

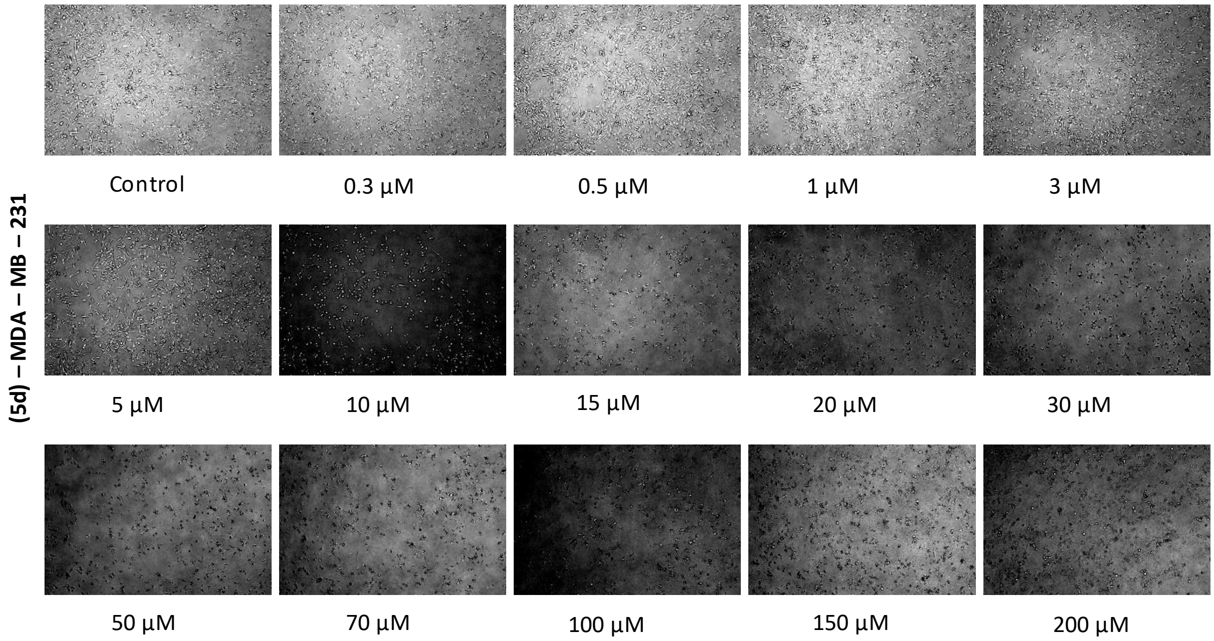

Figure S77. Microscope images of Compound **5d** in MDA-MB-231 cell line prepared at various concentrations

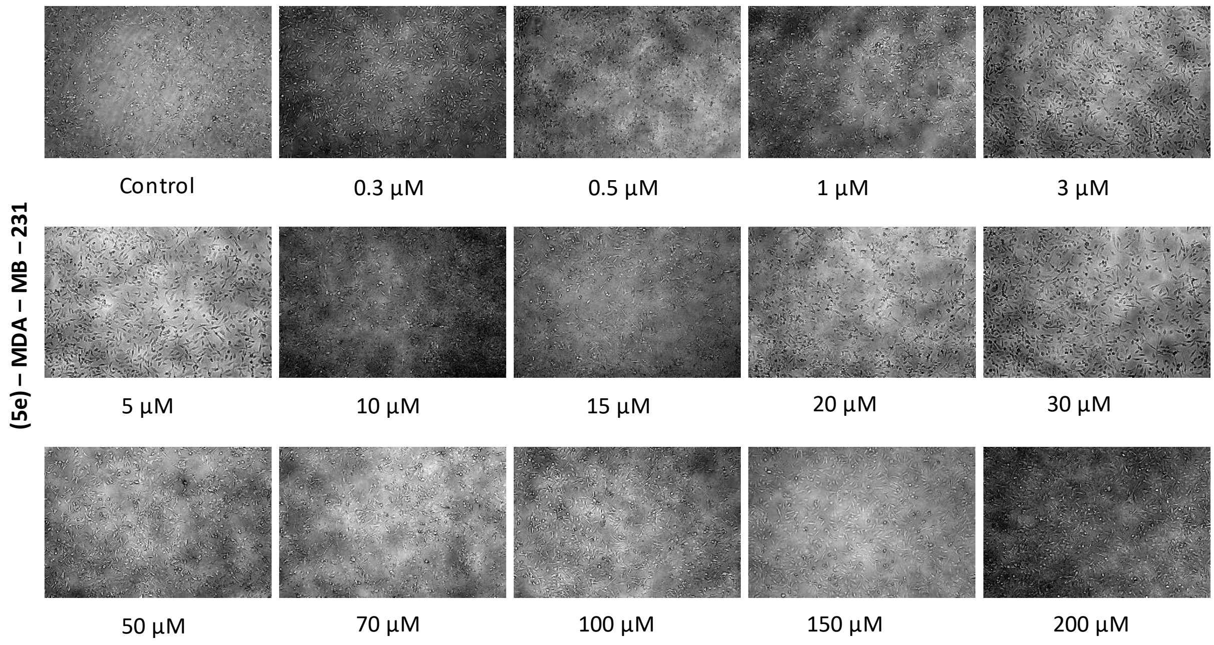

Figure S78. Microscope images of Compound **5e** in MDA-MB-231 cell line prepared at various concentrations

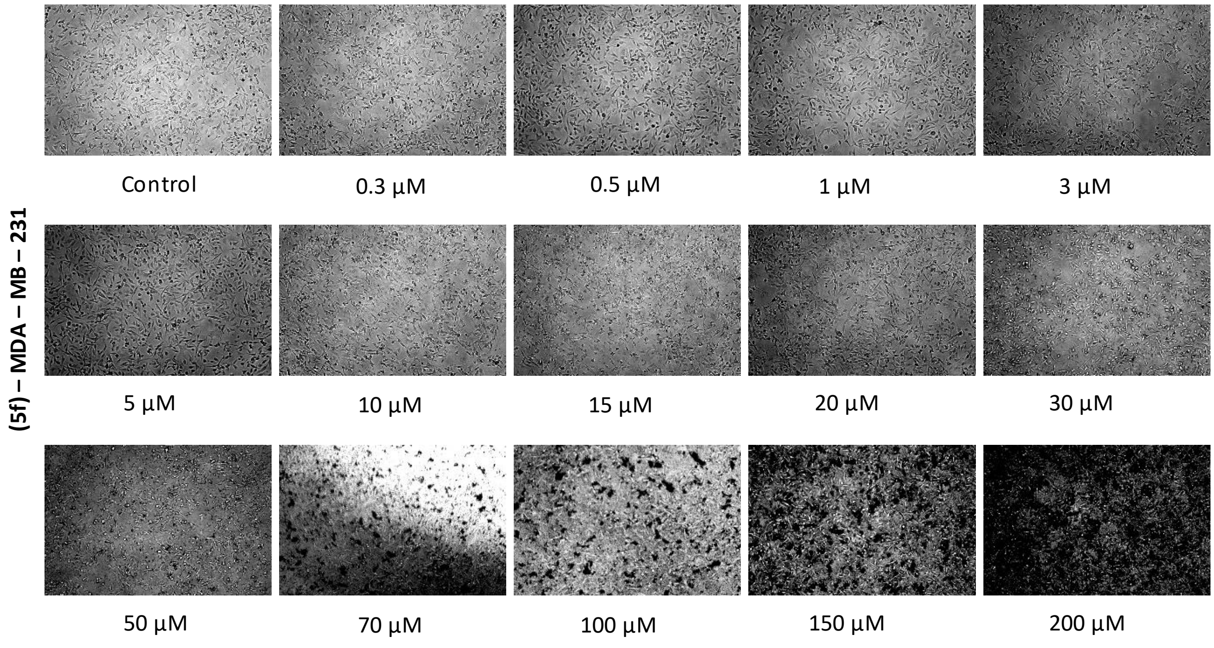

Figure S79. Microscope images of Compound **5f** in MDA-MB-231 cell line prepared at various concentrations

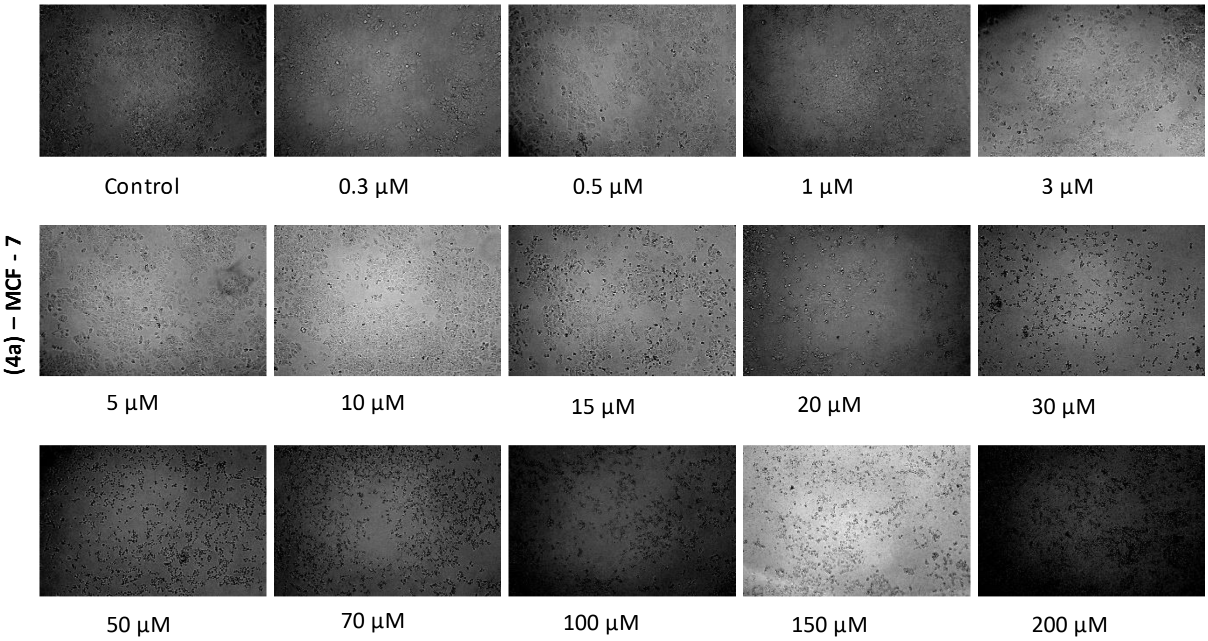

Figure S80. Microscope images of Compound **4a** in MCF-7 cell line prepared at various concentrations

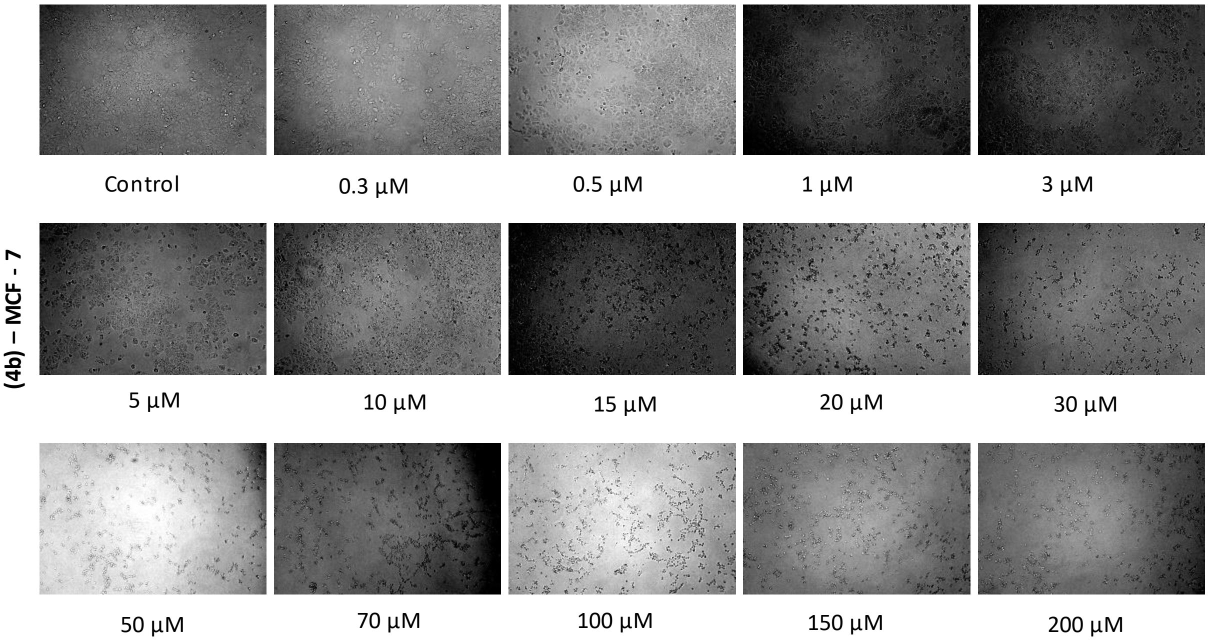

Figure S81. Microscope images of Compound **4b** in MCF-7 cell line prepared at various concentrations

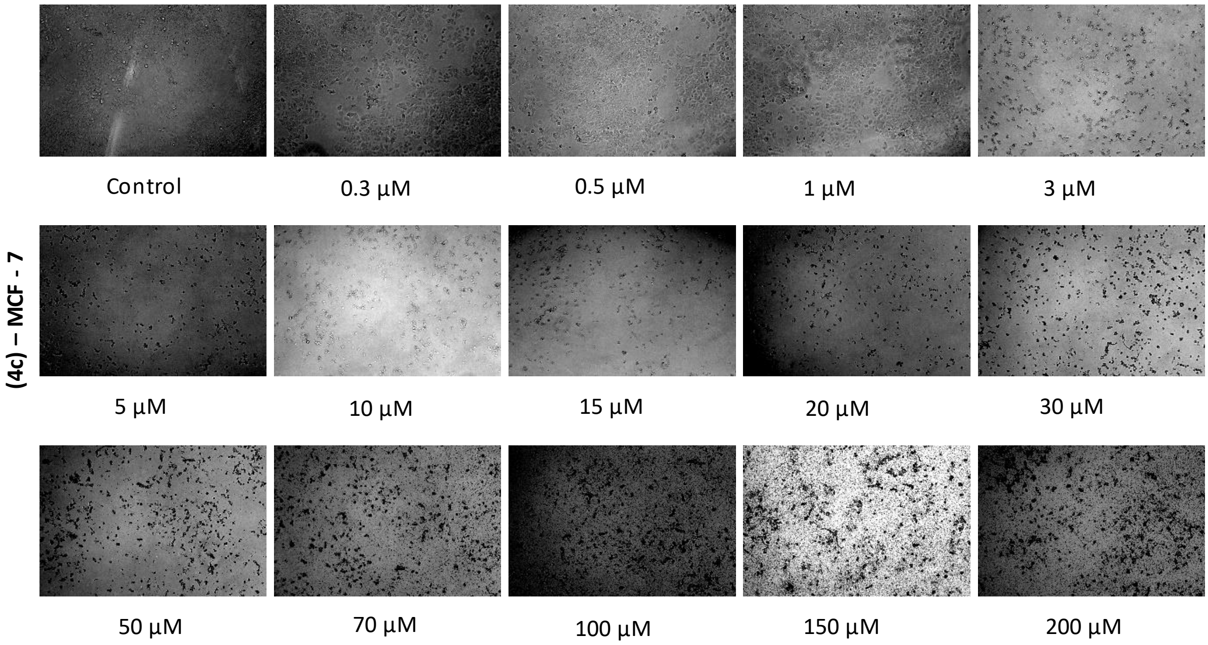

Figure S82. Microscope images of Compound **4c** in MCF-7 cell line prepared at various concentrations

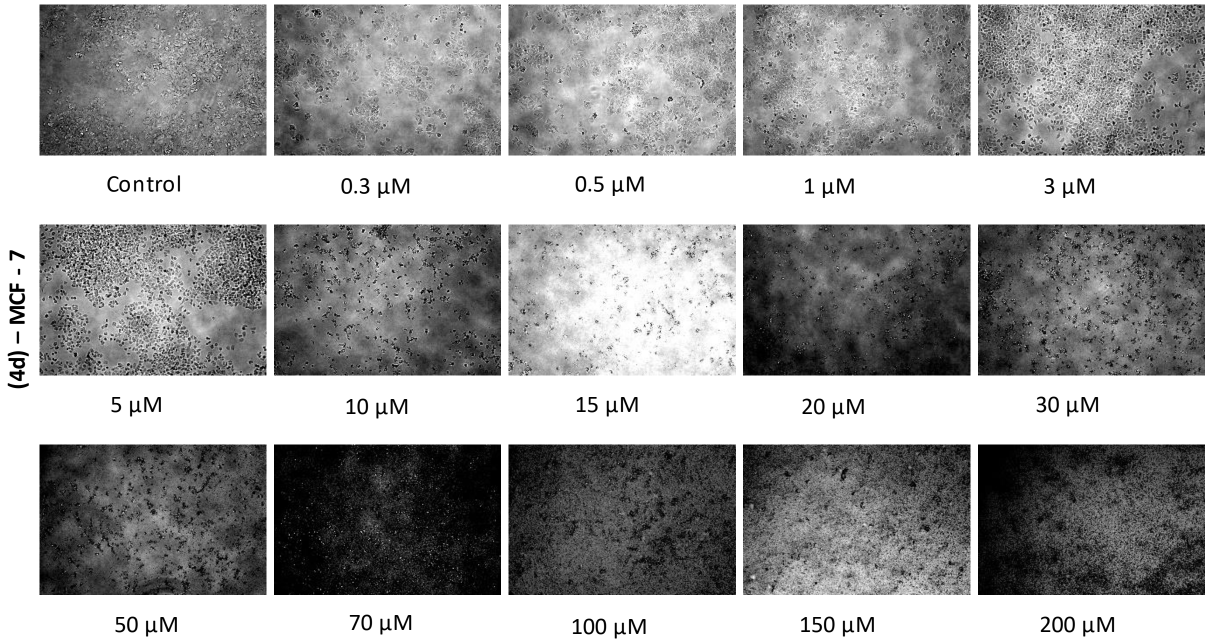

Figure S83. Microscope images of Compound **4d** in MCF-7 cell line prepared at various concentrations

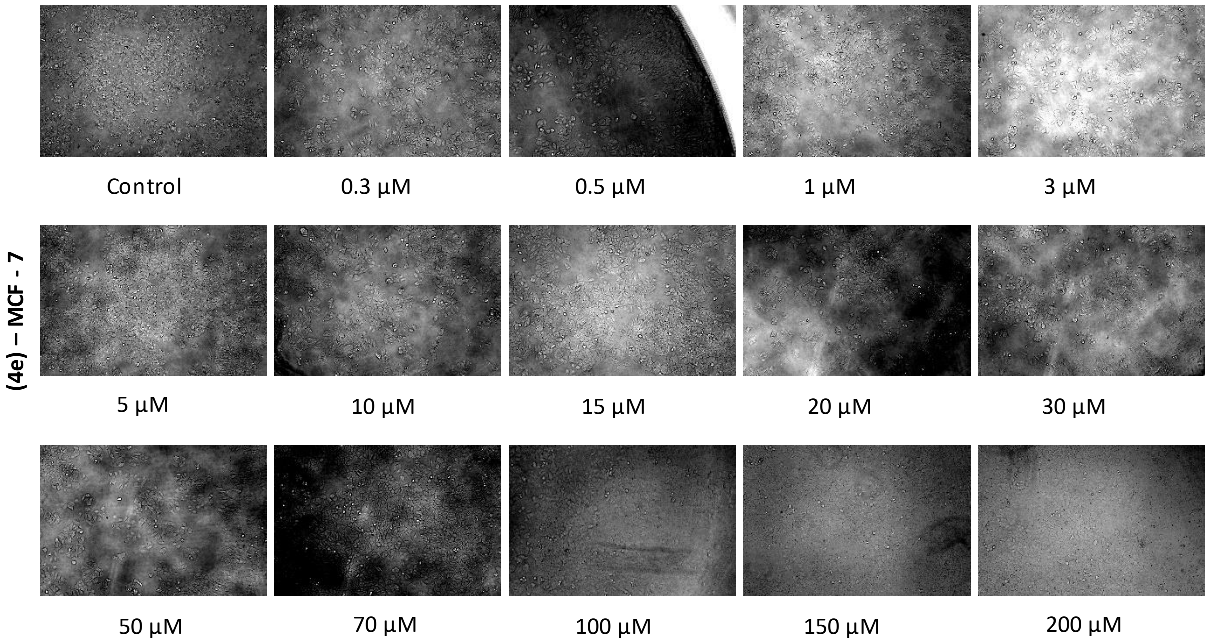

Figure S84. Microscope images of Compound **4e** in MCF-7 cell line prepared at various concentrations

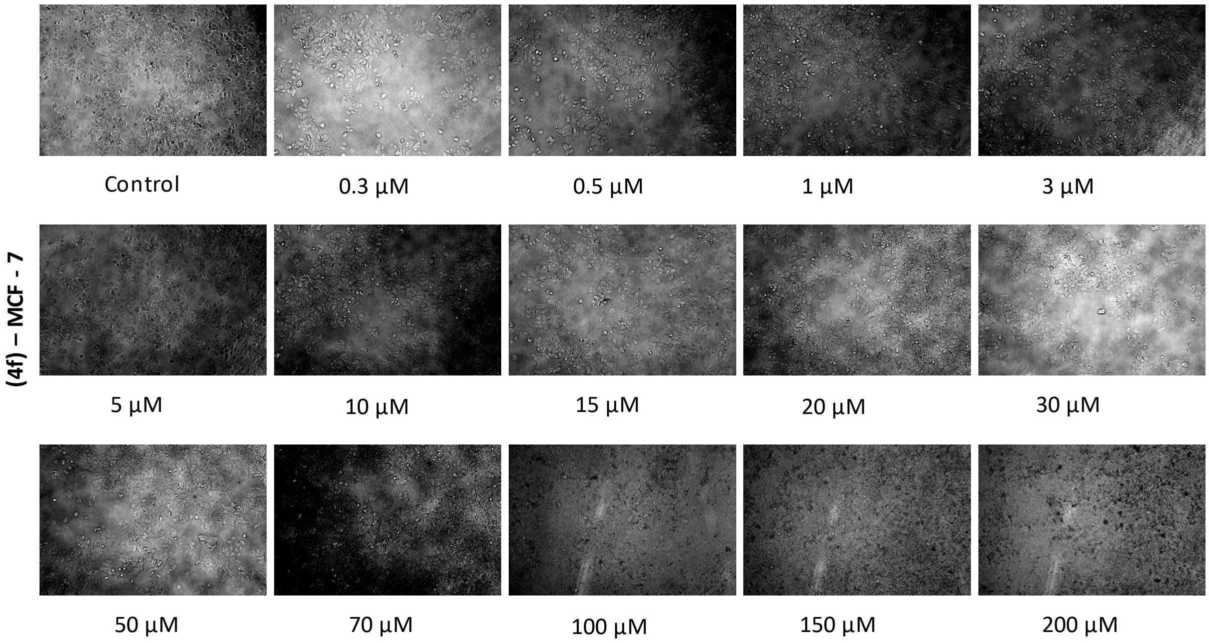

Figure S85. Microscope images of Compound **4f** in MCF-7 cell line prepared at various concentrations

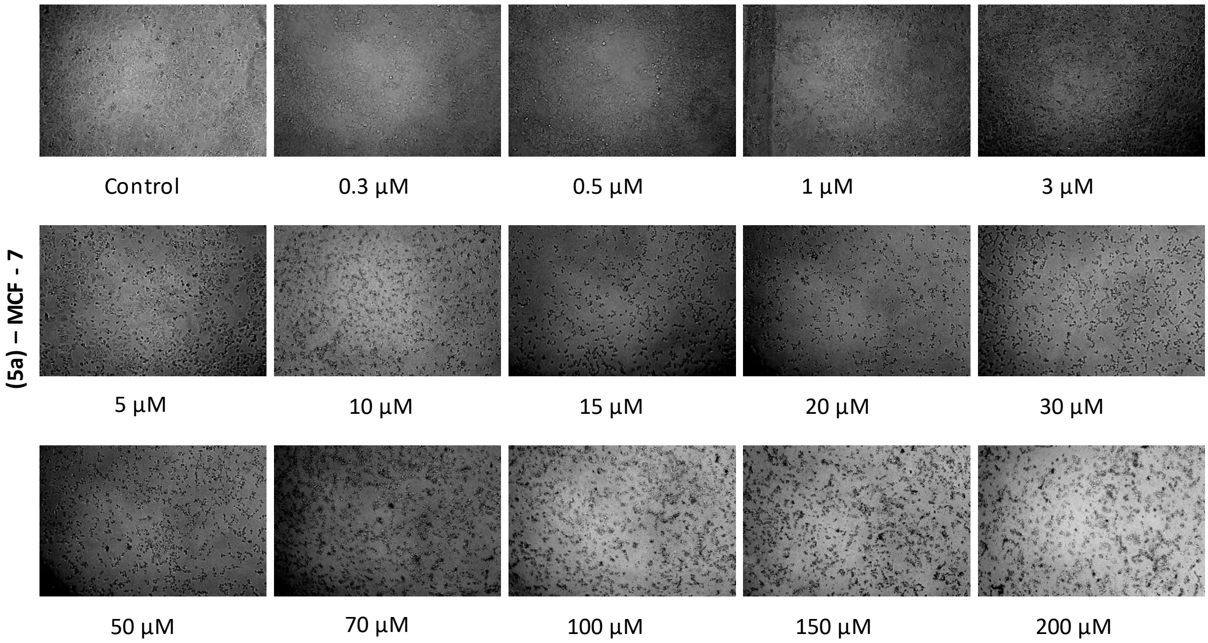

Figure S86. Microscope images of Compound **5a** in MCF-7 cell line prepared at various concentrations

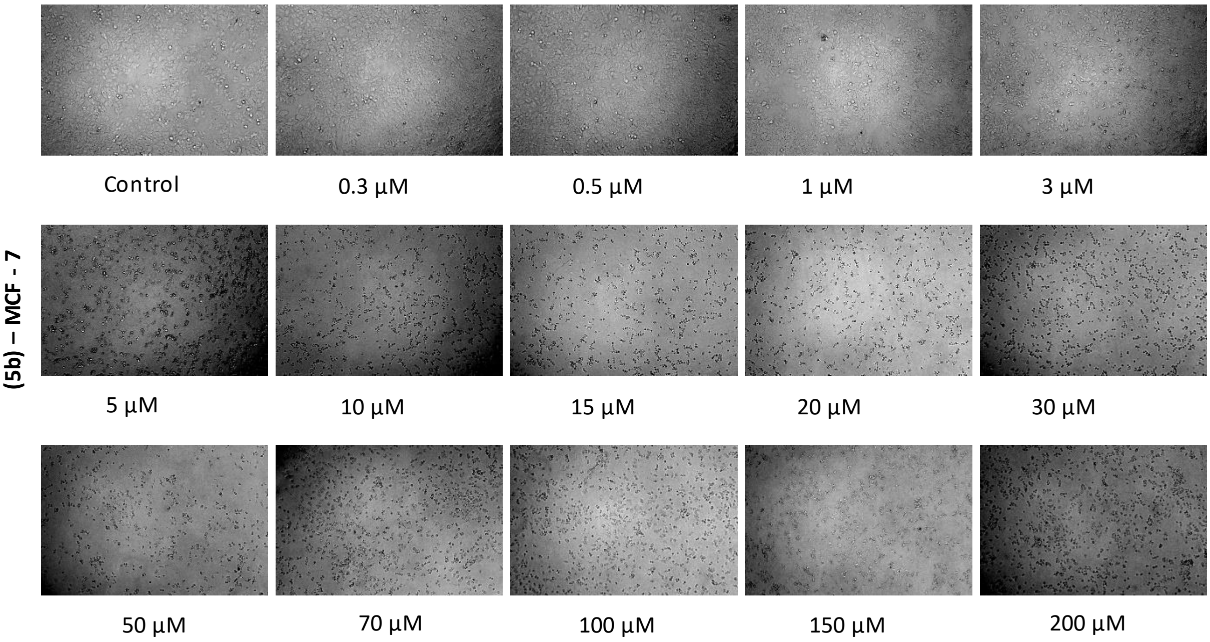

Figure S87. Microscope images of Compound **5b** in MCF-7 cell line prepared at various concentrations

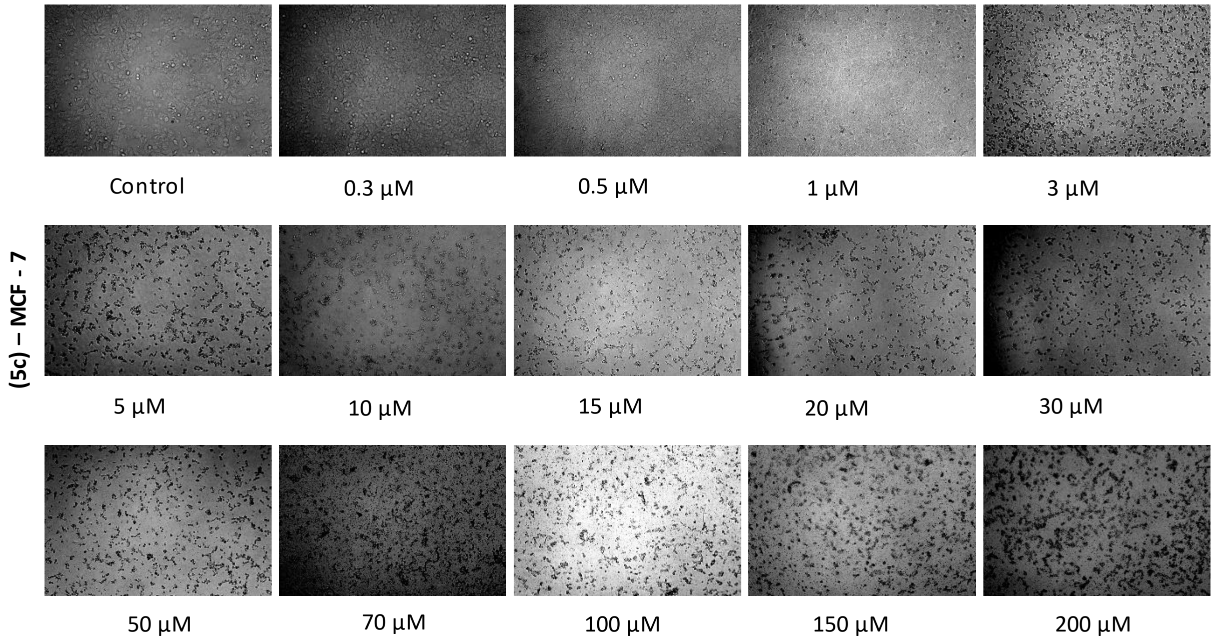

Figure S88. Microscope images of Compound **5c** in MCF-7 cell line prepared at various concentrations

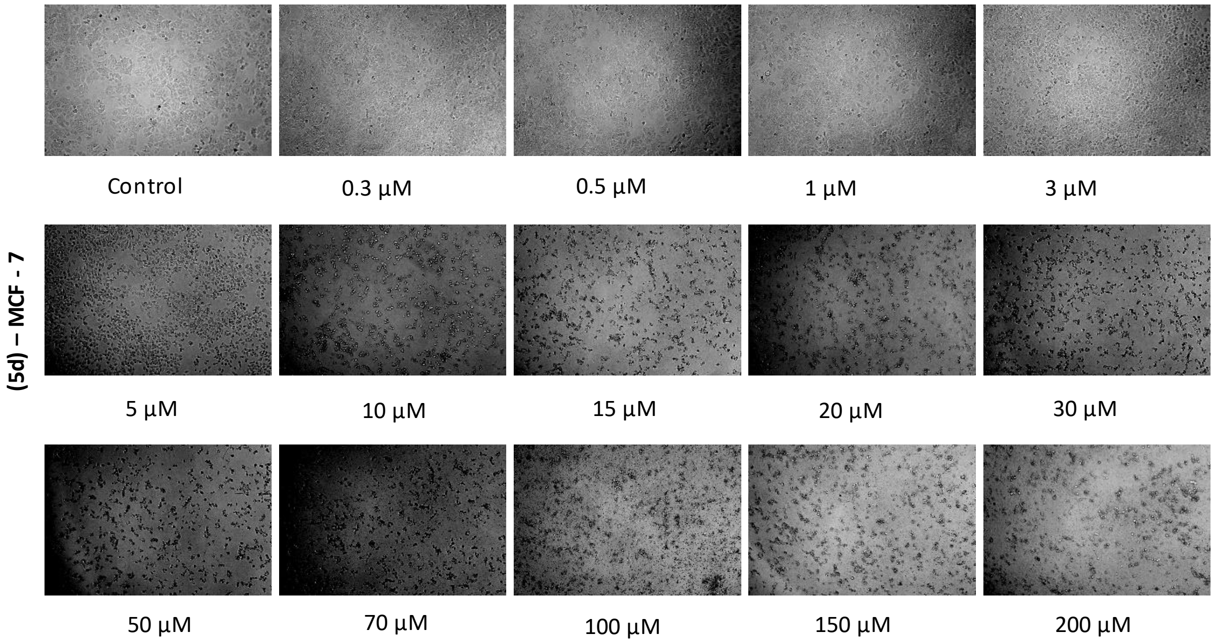

Figure S89. Microscope images of Compound **5d** in MCF-7 cell line prepared at various concentrations

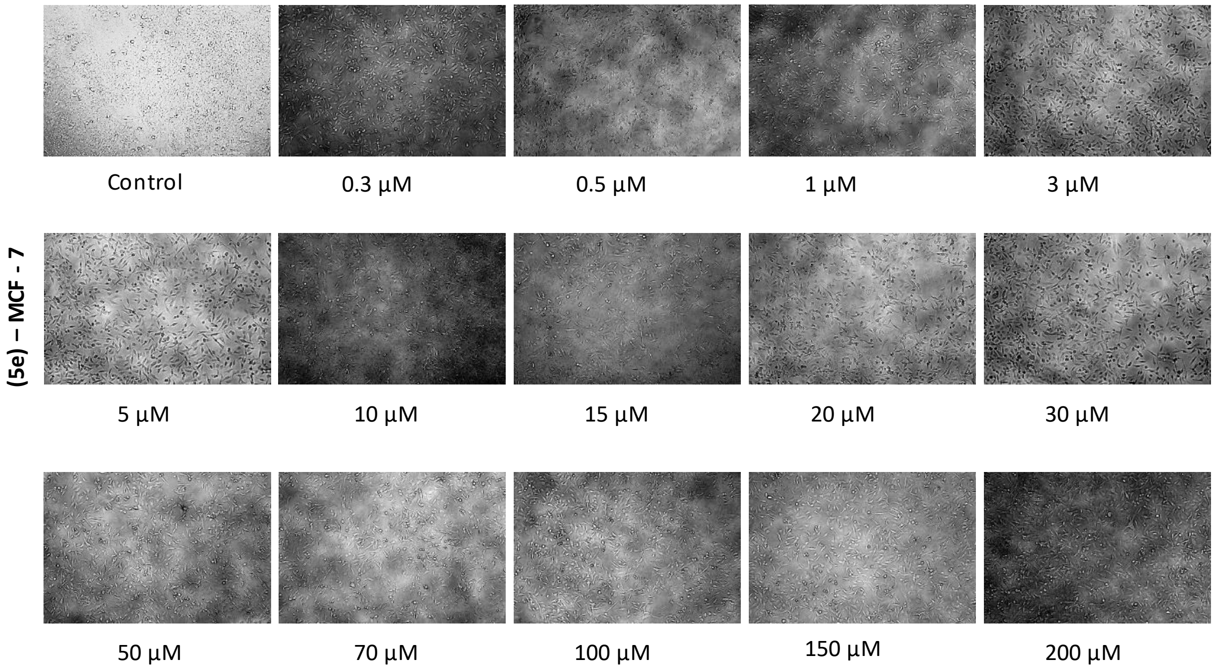

Figure S90. Microscope images of Compound **5e** in MCF-7 cell line prepared at various concentrations

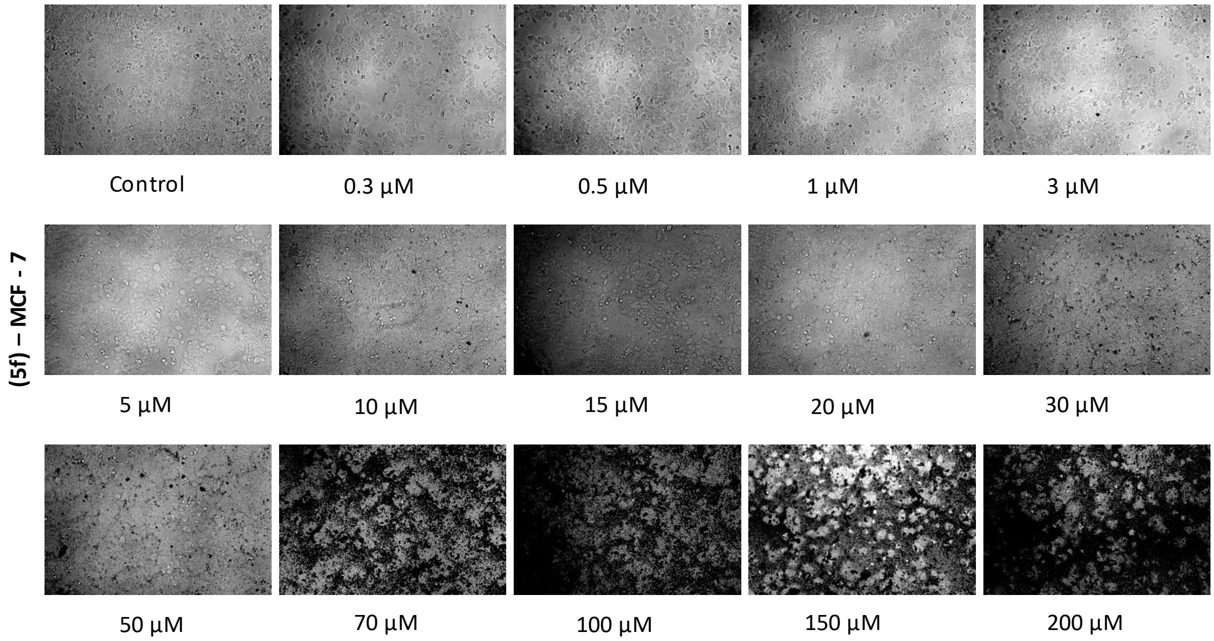

Figure S91. Microscope images of Compound **5f** in MCF-7 cell line prepared at various concentrations

3. Computational Methods

3.1 Molecular docking simulations

Molecular docking calculations were conducted using the Autodock 4.2 program. [9-] 100 independent calculations were performed for each ligand. The dimensions of the grid box (80 x 60 x 60) were selected to cover the catalytic cleft of GSK-3β (PDB ID: 4NMO) using AutoDockTools. [10] A value of 50 million was used for the maximum number of energy evaluation (ga_num_evals). For other parameters the default values were utilized.

3.2 Molecular Dynamic Simulations

Antechamber [11-12] Python Parser Interface (ACPYPE) [13] was utilized to obtain the topologies and parameters of the small molecules. The coordinates for each small molecule were obtained from the lowest energy conformation and then optimized using the semi-empirical Hamiltonian AM1. [14] The AM1-BCC charges were derived based on the optimized structure. [15-16] The bond lengths, angles, and dihedral force constants for the small molecules were adopted from General Amber Force Field (GAFF). [17]

The starting structures for molecular dynamics simulations were obtained from the lowest energy conformations of molecular docking calculations. Using Gromacs package [18], version 2021.2 [19], conventional molecular dynamics simulations were performed in explicit solvent for a duration of 100 ns. The protein-ligand complex was placed in a cubic box of TIP3P water [20], and the system was neutralized by adding counterions when required. Protein residues and ions were modeled using the Amber force field F14SB. [21] Short-range non-bonded interactions were truncated at a cutoff value of 1.2 nm, while long-range electrostatic interactions were treated using the particle mesh Ewald (PME) [22] method. Dispersion correction was applied to both energy and pressure, and periodic boundary conditions were applied in all directions.

The starting structures were minimized using 1000 steps of the conjugate gradient algorithm, with one step of steepest descent carried out every 10 steps. Following minimization, each system underwent equilibration via 100 ps of molecular dynamics simulations in the NVT ensemble, during which the heavy atoms of the protein were restrained harmonically using a force constant of 1000 kJ mol-1nm^{-2}. Temperature was maintained at 300 K using the velocity rescaling algorithm [23], with a coupling time of 0.5 ps. Solute and solvent atoms were coupled separately to temperature baths. To integrate Newton's equations of motion, we employed the leap-frog algorithm with a time step of 2 fs, while all bonds were constrained using the LINCS algorithm. [24] The NPT ensemble was used for data collection, in the absence of any restraints, with the pressure maintained at 1 bar using the Parrinello-Rahman barostat [25] with a time constant of 0.5 ps. Nose-Hoover temperature coupling method [26-27] was employed to regulate the temperature where the reference temperature and time constant set to 300 K and 0.5 ps, respectively.

We excluded the first 5 ns of the trajectories as a relaxation phase and calculated RMSF values using the ligand conformations after aligning only the protein conformations. The values presented in parentheses represent the corresponding standard deviations.

References:

[1] K. Hu, D. Patnaik, T. L. Collier, K. N. Lee, H. Gao, M. R. Swoyer, B. H. Rotstein, H. S. Krishnan, S. H. Liang, J. Wang, Z. Yan, J. M. Hooker, N. Vasdev, S. J. Haggarty, M. Ngai, *ACS Med. Chem. Lett*. **2017**, 8 (3), 287-292.

[2] Z. Guo, Y. Xu, Y. Peng, H. U. Rashid, W. Quan, P. Xie, L. Wu, J. Jiang, L. Wang, X. Liu, *Bioorg Med Chem Lett.*, **2019**, 29 (9), 1133-1137.

[3] S. Y. Zhao, Z. Y. Shao, W.-M. Qin, D.-Q. Zhang, *Chin. J. Org. Chem*. **2008**, 28 (10) 1676–1684.

[4] S. Jeffrey, K. L. Barnes, J. A. Eickhoff, C. R. Carson, J. *Am. Chem. Soc.* **2011**, 133, 7688-7691.

[5] A. H. Cory, T. C. Owen, J. A. Barltrop, J. G. Cory, *Cancer Commun.* **1991**, 3, 207-212.

[6] A. Daina, V. Zoete, *ChemMedChem*. **2016**, 11, 1117-1121.

[7] H. Özkan, Süleyman Demirel University Faculty of Arts and Sciences Journal of Science, **2019**, 14, 384-394.

[8] Khan, M. A. Tantray, H. Hamid, M. S. Alam, K. Sharma, P. Kesharwani, *Bioorg. Chem*. **2022**, 119, 105512.

[9] G. M. Morris, D. S. Goodsell, R. S. Halliday, R. Huey, W. E. Hart, R. K. Belew, A. J. Olson, *J. Comput. Chem*. **1998**, 19, 1639-1662.

[10] G. M. Morris, R. Huey, W. Lindstrom, M. F. Sanner, R. K. Belew, D. S. Goodsell, A. J. Olson, *J. Comput. Chem*. **2009**, 30, 2785-2791.

[11] J. M. Wang, W. Wang, P. A. Kollman, D. A. Case, *J. Am. Chem. Soc.* **2001**, 222, 403.

[12] J. W. Wang, P. A. Kollman, D. A. Case, *J. Mol. Graph. Model.* **2006**, 25, 247-260.

[13] A. W. Sousa da Silva and W. F. Vranken, *BMC Res. Notes*, **2012**, 5, 367.

[14] F. Pettersen, T. D. Goddard, C. C. Huang, G. S. Couch, D. M. Greenblatt, E. C. Meng, T.E. Ferrin, *J. Comput. Chem.* **2004**, 25, 1605-1612.

[15] A. Jakalian, B. L. Bush, D. B. Jack, C. I. Bayly, J. *Comput. Chem.* **2000**, 21, 132-146.

[16] R. C. Walker, M. F. Crowley, D. A. Case, *J. Comput. Chem*. **2008**, 29, 1019-1031.

[17] J. Wang, R. M. Wolf, J. W. Caldwell, P. A. Kollman, D. A. Case, J. Comput. Chem. 2004, 25, 1157-1174.

[18] Van Der Spoel, E. Lindahl, B. Hess, G. Groenhof, A. E. Mark, H. J. C. Berendsen, *J. Comput. Chem*. **2005**, 26, 1701-1718.

[19] M. J. Abraham, T. Murtola, R. Schulz, S. Páll, J. C. Smith, B. Hess, E. Lindahl, *SoftwareX*. **2015**, 1–2, 19–25.

[20] Jorgensen, W. *J. Am. Chem. Soc.* 1981, 103, 335-340.

[21] J. A. Maier, C. Martinez, K. Kasavajhala, L. Wickstrom, K. E. Hauser, C. Simmerling, *JCTC*. **2015**, 11, 3696-3713.

[22] U. Essmann, L. Perera, M. L. Berkowitz, *J. Chem. Phys*. 1995, 103, 8577-8593.

[23] Bussi, D. Donadio, M. Parrinello, *J. Chem. Phys*, **2007**, 126, 014101.

[24] A. Hess, *J. Chem. Theory Comput*. **2008**, 4, 116-122.

[25] M. Parrinello, A. Rahman, *J. Appl. Phys.* **1981**, 52, 7182-7190.

[26] S. Nose, *J. Chem. Phys.* **1984**, 81, 511-519.

[27] W. G. Hoover, *Phys. Rev. A.* **1985**, 31, 1695-1697
